# Evidence-based expert consensus on the management of primary central nervous system lymphoma in China

**DOI:** 10.1186/s13045-022-01356-7

**Published:** 2022-09-29

**Authors:** Tong Chen, Yuanbo Liu, Yang Wang, Qing Chang, Jinsong Wu, Zhiliang Wang, Daoying Geng, Jin-Tai Yu, Yuan Li, Xiao-Qiu Li, Hong Chen, Dongxiao Zhuang, Jianyong Li, Bin Wang, Tao Jiang, Lanting Lyu, Yuqin Song, Xiaoguang Qiu, Wenbin Li, Song Lin, Xinghu Zhang, Dehong Lu, Junqiang Lei, Yaolong Chen, Ying Mao

**Affiliations:** 1 Department of Hematology, Institute of Medicine, Huashan Hospital, Fudan University, Shanghai, 200040 China; 2 Department of Hematology, Beijing Tiantan Hospital, Capital Medical University, Beijing, 100070 China; 3 Department of Radiation Oncology, Huashan Hospital, Fudan University, Shanghai, 201107 China; 4 National Center for Neurological Disorders, Huashan Hospital, Fudan University, Shanghai, 200040 China; 5 Department of Ophthalmology, Eye and ENT Hospital, Fudan University, Shanghai, 200031 China; 6 Department of Neurosurgery, National Center for Neurological Disorders, Huashan Hospital, Fudan University, Shanghai, 200040 China; 7 Institute of Medicine, Huashan Hospital, Fudan University, Shanghai, 200040 China; 8 Department of Ophthalmology, Huashan Hospital, Fudan University, Shanghai, 200040 China; 9 Department of Radiology, Huashan Hospital, Fudan University, Shanghai, 200040 China; 10 Department of Neurology, Huashan Hospital, Fudan University, Shanghai, 200040 China; 11 Department of Pathology, Fudan University Shanghai Cancer Center, Shanghai, 200032 China; 12 Department of Pathology, Huashan Hospital, Fudan University, Shanghai, 200040 China; 13 Department of Hematology, The First Affiliated Hospital of Nanjing Medical University, Jiangsu Province Hospital, Nanjing, 210029 China; 14 Department of Pharmacy, Huashan Hospital, Fudan University, Shanghai, 200040 China; 15 Department of Neurosurgery, Beijing Neurosurgical Institute, Beijing Tiantan Hospital, Capital Medical University, Beijing, 100070 China; 16 School of Public Administration and Policy, Health Technology Assessment and Policy Evaluation Group, Renmin University of China, Beijing, 100872 China; 17 Department of Lymphoma, Peking University Cancer Hospital and Institute, Beijing, 100142 China; 18 Department of Radiation Oncology, Beijing Tiantan Hospital, Capital Medical University, Beijing, 100070 China; 19 Department of Neuro-Oncolgoy, Cancer Center, National Clinical Research Center for Neurological Diseases, Beijing Tiantan Hospital, Capital Medical University, Beijing, 100070 China; 20 Department of Neurology, Neuroimmunology and Neuroinfection Center, Beijing Tiantan Hospital, Capital Medical University, Beijing, 100070 China; 21 Department of Pathology, Xuanwu Hospital, Capital Medical University, Beijing, 100053 China; 22 Department of Radiology, The First Hospital of Lanzhou University, Lanzhou, 730000 China; 23 Research Unit of Evidence-Based Evaluation and Guidelines, Chinese Academy of Medical Sciences (2021RU017), School of Basic Medical Sciences, Lanzhou University, Lanzhou, 730000 China; 24 WHO Collaborating Center for Guideline Implementation and Knowledge Translation, Lanzhou University, Lanzhou, 730000 China; 25 Lanzhou University GRADE Center, Lanzhou, 730000 China

**Keywords:** Consensus, Primary central nervous system lymphoma, Management, China

## Abstract

Primary central nervous system lymphoma (PCNSL) is a type of central nervous system restricted non-Hodgkin lymphoma, whose histopathological diagnosis is majorly large B cell lymphoma. To provide specific, evidence-based recommendations for medical professionals and to promote more standardized, effective and safe treatment for patients with PCNSL, a panel of experts from the Chinese Neurosurgical Society of the Chinese Medical Association and the Society of Hematological Malignancies of the Chinese Anti-Cancer Association jointly developed an evidence-based consensus. After comprehensively searching literature and conducting systematic reviews, two rounds of Delphi were conducted to reach consensus on the recommendations as follows: The histopathological specimens of PCNSL patients should be obtained as safely and comprehensively as possible by multimodal tomography-guided biopsy or minimally invasive surgery. Corticosteroids should be withdrawn from, or not be administered to, patients with suspected PCNSL before biopsy if the patient’s status permits. MRI (enhanced and DWI) should be performed for diagnosing and evaluating PCNSL patients where whole-body PET-CT be used at necessary time points. Mini-mental status examination can be used to assess cognitive function in the clinical management. Newly diagnosed PCNSL patients should be treated with combined high-dose methotrexate-based regimen and can be treated with a rituximab-inclusive regimen at induction therapy. Autologous stem cell transplantation can be used as a consolidation therapy. Refractory or relapsed PCNSL patients can be treated with ibrutinib with or without high-dose chemotherapy as re-induction therapy. Stereotactic radiosurgery can be used for PCNSL patients with a limited recurrent lesion who were refractory to chemotherapy and have previously received whole-brain radiotherapy. Patients with suspected primary vitreoretinal lymphoma (PVRL) should be diagnosed by vitreous biopsy. PVRL or PCNSL patients with concurrent VRL can be treated with combined systemic and local therapy.

**Supplementary Information:**

The online version contains supplementary material available at 10.1186/s13045-022-01356-7.

## Background

Primary central nervous system lymphoma (PCNSL) is one type of extra-nodal non-Hodgkin lymphoma (NHL), where involvement is restricted to the brain, spinal cord, cranial nerves, leptomeninges and vitreo-retina [1, 2]. It is a relatively rare tumor, accounting for about 1% of NHLs and 3–4% of intracranial tumors [3]. Pathologically, more than 95% of PCNSL cases are diffuse large B cell lymphoma (DLBCL), which is listed as a separate entity in the *World Health Organization (WHO) Classification of Tumours of Haematopoietic and Lymphoid tissues 2016* [4] and *WHO Classification of Tumours of the Central Nervous System 2021* [5]. Thus, the recommendations in this consensus focus exclusively on PCNS-DLBCLs. Histologic diagnosis of PCNSL is based on immunohistochemical detection, flow cytometric analysis, cytokines detection, immunoglobulin heavy chain (IgH) gene rearrangement analysis, polymerase chain reaction (PCR) detection and high-throughput genome sequencing. Few drugs are appropriate for use in treating PCNSL given the need for effective concentrations within the CNS, requiring efficient penetration of the blood–brain barrier (BBB) at the molecule weight < 400-600D [6]. Consequently, the standard regimens of treating DLBCLs, such as rituximab (R), cyclophosphamide, doxorubicin, vincristine and prednisone (R-CHOP), do not produce a good response in PCNSL patients. A specialized evidence-based expert consensus for the clinical management of PCNSL is therefore needed.

Several clinical consensus/guidelines are available for the diagnosis and treatment of PCNSL, including those issued by the British Neuro-Oncology Society (BNOS) (published in 2011), the European Association for Neuro-Oncology (EANO) (2015), and the British Society for Haematology (BSH) (2019) [7–9]. In the USA, the National Comprehensive Cancer Network (NCCN) provides routine updates of their guidelines [10]. However, no specialized evidence-base consensus for clinical practice exclusively focusing on PCNSL is currently available in China. Recommendations on PCNSL were included in those on lymphoid malignancies issued by the Chinese Society of Clinical Oncology (CSCO) for the first time in 2020 [11] and were updated in 2021 and 2022.

In addition, there are still unmet needs that would be benefit from consensus recommendations. The guidelines issued by BNOS and EANO may be become outdated, and the CSCO and NCCN recommendations were developed and published as part of ones on lymphomas or central nervous system cancers, some of which provided recommendations without the evidence interpretation. The CSCO guidelines for primary intraocular lymphoma only address diagnostic evaluation and do not include treatment. In view of these gaps, the Chinese Neurosurgical Society of the Chinese Medical Association (CNS-CMA) and the Society of Hematological Malignancies of the Chinese Anti-Cancer Association (SHM-CACA) jointly initiated a multidisciplinary team to develop an evidence-based consensus focusing on PCNSL (hereinafter referred to “the consensus”).

The CNS-CMA and the SHM-CACA initiated and led development of the consensus. There were a number of working groups which contributing to the consensus, each with specific roles and responsibilities: consensus steering committee, conflict of interest management committee, secretariat, expert consensus group, evidence evaluation group, and external review group. Both the CNS-CMA and the SHM-CACA recommended the experts by evaluating their experiences in treating PCNSL at their daily work and previous publication. The expert consensus group encompassed experts from diverse geographic regions in China and disciplines, including neurosurgery, hematology, neurology, ophthalmology, oncology, radiotherapy, imaging, pathology, evidence-based medicine, pharmacology, and health economics. All the members in the expert consensus group completed a disclosure of interest form prior to commencing their participation in the consensus. Four experts not previously involved in development of the consensus were invited to review the draft consensus.

The importance of the interested clinical questions (CQs) was rated by the consensus members via an email questionnaire. After two rounds of investigation, 11 CQs were prioritized and presented to evidence search and evaluation (Additional file 1). A high-quality, up-to-date systematic review formed the basis of all recommendations (see Additional file 2 for detailed search strategy). If such a review could not be identified in the published literature, an existing review was updated or a new review was performed. All the data of meta-analysis or systematic review focusing on interested CQs were listed in supplementary materials (Additional file 3). The GRADE system was also used to determine the strength of recommendations (strong or weak) (Table 1). The consensus of a total of 13 recommendations was formulated after two rounds of Delphi, considering the quality of the evidence, patient preference and values, balance of benefits and harms, resource utilization, equity, and acceptability (Table 2, Additional file 4).

**Table 1:** Quality of evidence and strength of recommendations

Grade	Content
*Quality of evidence*	
High (A)	We are very confident that the actual effect lies close to that of the estimate of the effect
Moderate (B)	We are moderately confident of the effect estimate: The actual effect is likely to be close to the evaluation of the effect, but there is a possibility that it is substantially different
Low (C)	Our confidence in the effect estimate is limited: The true effect may be substantially different from the evaluation of the effect
Very low (D)	We have very little confidence in the effect estimate: The true effect is likely to be substantially different from the estimate of effect
*Strength of recommendations*	
Strong (1)	Clearly shows the benefits of intervention outweigh the harms or more harms than benefits
Weak (2)	Uncertain about the benefits and harms or the benefits and harms are tantamount regardless of the quality of evidence

**Table 2:** Summary of the recommendations

Management	Recommendations
Biopsy and diagnosis	We suggest the histopathological specimens of PCNSL patients should be obtained as safely and comprehensively as possible by multimodal tomography-guided biopsy or minimally invasive surgery (2C)
	We recommend that corticosteroids should be withdrawn from, or not be administered to, patients with suspected PCNSL before biopsy, if the patient’s status so permits (1C)
	We suggest that corticosteroids should be withdrawn from patients with suspected PVRL at least 2 weeks before biopsy, if the patient’s status permits (2D)
Staging and following-up evaluation	We recommend MRI (enhanced and DWI) for the diagnosis and evaluation of PCNSL patients (1B)
	We suggest that whole-body PET-CT be used to evaluate PCNSL patients at certain time points, such as the time of initial diagnosis or at relapse (2B)
	We suggest that MMSE be used to assess cognitive function in the management of PCNSL patients (2B)
Induction therapy	We recommend that newly diagnosed PCNSL patients should be treated with a combined HD-MTX-based regimen, if the patient is fit for chemotherapy (1B). The combined therapeutics can be rituximab (2C), cytarabine (2B), temozolomide (2C) or other drugs which can cross the BBB (2C)
	We suggest that newly diagnosed PCNS-DLBCL patients can be treated with a rituximab-inclusive regimen at induction therapy (2C)
Consolidation therapy	Compared with non-reduced dose WBRT, we suggest that ASCT can be used as consolidation therapy for PCNSL patients who are fit for conditioning chemotherapy (2C)
Refractory/relapsed	We suggest that refractory or relapsed PCNSL patients can be treated with ibrutinib with or without high-dose chemotherapy as re-induction therapy (2C)
	We suggest that stereotactic radiosurgery can be used for PCNSL patients with a limited recurrent lesion who were refractory to chemotherapy and have previously received WBRT (2D)
Intraocular involvement	We suggest that patients with suspected of PVRL should be diagnosed by vitreous biopsy (2D)
	We suggest that PVRL patients or PCNSL patients with concurrent VRL can be treated with combined systemic and local therapy (2C)

## Approach to diagnosis

### Biopsy

Recommendation: We suggest the histopathological specimens of PCNSL patients should be obtained as safely and comprehensively as possible by multimodal tomography-guided biopsy or minimally invasive surgery (2C).

Multiple lesions are common in PCNSL patients, which usually involve the deep tissues of the brain. Resection of these deep lesions may result in postoperative neurological dysfunction. The operation will be even more challenging if there exists a cyst or the edge of tumor is unclear. Therefore, obtaining pathology specimens by resection was not recommended for PCNSL patients [12]. In some studies, it was reported that patients undergoing resection (total or subtotal) had a greater chance of complete remission (CR) 6 months postoperatively. But no significant improvement in OS was observed between the biopsy and resection groups, and the open resection procedure was associated with high complication rates [13, 14]. However, most of these studies were of lower quality and were performed before the wide application of current chemotherapy. As high-dose (HD) methotrexate (MTX)-based chemotherapy results in optimal cytoreduction of PCNSL, the purpose of the operation is to obtain the sufficient specimen for diagnosis rather than to remove the tumor mass, in order to maintain the patients’ neurological function and to better their quality of life (QoL).

By updating the systematic review published by Labak et al. in 2019 [15] and analyzing twenty-two retrospective studies in total, no significant difference in the complication incidence was shown between PCNSL patients undergoing resection and those undergoing biopsy (RR = 1.00, 95% CI 0.65–1.54, *P* = 0.244). Considering the enormous technical changes in resection, chemotherapy, radiotherapy and biopsy in recent years, the studies published before or after 2010 were analyzed separately. There was no significant difference in the complication incidence between resection and biopsy of these two time periods (before 2010, RR = 1.00, 95% CI 0.44–2.30, *P* = 0.102; after 2010, RR = 1.00, 95% CI 0.60–1.65, *P* = 0.403). Eight papers published in recent 5 years were identified in our update of the Labak review: all showed that surgical resection may bring more benefit. However, most of these studies didn’t analyze postoperative neuro-dysfunction, and some of them included the cases before HD-MTX was widely applied as the standard chemotherapeutics (Additional file 3: CQ 1). Thus, for the purpose of obtaining the pathology specimen and making diagnosis, attention should be paid more to patients’ safety rather than cytoreduction for PCNSL, in order to move on to chemotherapy.

In addition, these early studies of open resection were carried out before the widespread use of MR imaging, neuro-navigation and fluorescent guiding operations, all of which improve the safety of current neurosurgical techniques. Considering the low level of evidence in earlier studies, the high incidence of neurological dysfunction with deep lesion resection, as well as the recommendations provided by other guidelines/consensus and current clinical practice experience, we recommend multimodal tomography-guided biopsy or minimally invasive surgery to obtain pathology specimens. For those patients with a superficial and single lesion whose neurologic function can be well maintained postoperatively, total resection rather than subtotal resection can be considered.

### Preoperative corticosteroid treatment

Recommendation: We recommend that corticosteroids should be withdrawn from or not be administered in the patients with suspected PCNSL before biopsy, if the patient’s status so permits (1C). We suggest that corticosteroids should be withdrawn from the patients with suspected primary vitreoretinal lymphoma (PVRL) at least 2 weeks before biopsy, if the patient’s status permits (2D).

Corticosteroid administration is the principal component of PCNSL therapeutic regimens, as it reduces intracranial pressure in patients at risk of brain herniation. PVRL patients previously misdiagnosed with uveitis usually receive corticosteroids to relieve symptoms. However, corticosteroids can induce lymphocytic apoptosis or lysis, resulting in the difficulty for the pathologist to make an accurate diagnosis after steroid treatment [16–19]. The guidelines of BSH (2019) [9], EANO (2015) [8], and NCCN (2012) [20] all recommend withdrawal of corticosteroids before biopsy. A re-evaluation of the tumor lesion by enhanced MRI is required if preoperative steroids are administrated.

There have been no prospective controlled studies focusing on whether corticosteroids should be withdrawn from PCNSL patients before biopsy (Additional file 3: CQ 2). Some retrospective studies showed no significant influence of corticosteroids on the accuracy of pathological diagnosis [21–24]. However, these studies all had limitations which lowered the quality of the evidence. They were therefore not used to support this recommendation. A retrospective study of 25 PCNSL treated preoperatively with steroids (2–30 days, mean 5 days) showed that the diagnostic accuracy of single biopsy occurred in only 13 of 25 patients [16]. Another retrospective study analyzed 76 PCNSL patients, including 32 cases with open surgical biopsy and 44 with stereotactic biopsy. The results showed that 33.3% patients with steroid treatment ≤ 1 week had false negative diagnosis, while 57.1% patients with steroids treatment > 1 week did [17]. Some case reports (2006, *n* = 4 [18]; 1990, *n* = 2 [19]) also showed that corticosteroids would influence the accuracy of a pathology diagnosis.

In a retrospective study analyzing PCNSL patients treated with preoperative steroids for a mean duration of 5 days, only 1/18 patients did not have a diagnostic biopsy, indicating that short-term preoperative treatment with steroids would not interfere with the pathological diagnosis [22]. The uncontrolled size of intracranial lesions in the corticosteroid-treated group might be one reason for reaching that conclusion, indicating the low quality of the evidence. Some studies reached conclusions that were not supported by the evidence. For example, in a study analyzing 135 patients who received steroids before biopsy and the 20 patients didn’t, the nonsteroid group had a higher rate of PCNSL diagnosis than the steroid treated group (5/20 vs. 15/135). However, the authors concluded no difference between the two groups (*P* = 0.99) [21]. Similar results were seen in other studies with only 4–8 patients [23, 24]. We didn’t use these studies in our recommendation due to their low quality.

Considering the essential role of a biopsy and pathology in the diagnosis, we recommend suspending the use of corticosteroids in patients with an intracranial tumor confirmed by enhanced MRI imaging, if the patient’s status permits corticosteroid withdrawal. In patients whose intracranial tumor is no longer identifiable after treatment with corticosteroids, withdrawal of corticosteroids is recommended, with close monitoring with MRI imaging. A biopsy should also be performed with any recurrence or progression of the tumor.

Regarding the questions as to how long the corticosteroids should be discontinued, the optimal duration for corticosteroid withdrawal has not been studied yet. The withdrawal time in the eligible studies, ranging from 1 day to 6 months preoperatively. Two reviews suggested suspending steroid for at least one-week before biopsy [25, 26]. However, two international expert consensus groups (2021 [27] and 2009 [28]) recommend suspending corticosteroids for at least two weeks before diagnostic vitrectomy. To provide more reliable evidence, well-designed prospective studies will be needed in the future.

## Imaging examination

Recommendation: We recommend MRI (enhanced and DWI) for the diagnosis and evaluation of PCNSL patients (1B). We suggest that whole-body PET-CT be used to evaluate PCNSL patients at certain time points, such as the time of initial diagnosis or at relapse (2B).

Imaging examination can noninvasively display the location, size and shape of intracranial lesions, which is helpful for diagnosis, staging and response monitoring for PCNSL. Imaging findings at diagnosis of PCNSL vary with the patient’s immune status. The detailed manifestations of MRI imaging in immunocompetent patients or immunocompromised patients have been compared in some literature studies [29, 30]: Typical presentations of PCNSL are single or multiple intracranial lesions, with the latter more common in immunodeficient patients. The tumor mass frequently arises from the deep white matter in the supratentorial frontotemporal lobe, with invasion into the proximal midline structures such as the basal ganglia and corpus callosum. Some patients also have intraocular lymphoma. The MRI image usually shows lesions distributed in the cranial periventricular structures, with a clear boundary and mild to moderate peritumoral edema. In immunocompetent patients, enhanced scanning often presents with “fist,” “incision,” or “angular” signs, while most immunodeficient patients present with ring-enhancement [31]. However, hemorrhage and calcification associated with immune reaction and radiochemotherapy, respectively, are atypical in PCNSL.

In addition, functional MRI imaging, such as diffusion-weighted imaging (DWI), perfusion-weighted imaging, magnetic resonance spectroscopy (MRS), and susceptibility-weighted imaging (SWI) are helpful to differentiate PCNSL from glioblastoma (GBM), intracranial metastatic tumor and Toxoplasma Gondii infection in patients with immune deficiency [29]. However, rare pathological types of PCNSL, for example intravascular lymphoma, are very difficult to diagnose by imaging. Routine enhanced MRI is recommended during the course of PCNSL treatment and monitoring, including remission, progression, and recurrence.

PET-CT is sensitive for PCNSL lesions and can help to determine the extent of the tumor and any involvement of surrounding tissues. The uptake of FDG is higher in PCNSL than in GBM and metastatic brain tumors [29]. Therefore, PET-CT is recommended as one of the diagnostic and staging methods for PCNSL at time points, such as at the time of initial diagnosis and with suspected recurrence. The complementarity among different imaging examinations can improve the accuracy of diagnosis and evaluation. The cost-effectiveness of the imaging modalities needs to be studied.

No study compared the preference for PCNSL patients between MRI and whole-body PET-CT (Additional file 3: CQ 3). There was a systematic review focusing on the diagnostic efficiency of PET-CT in PCNSL patients [32]. It included 29 eligible studies, showing that the diagnostic sensitivity and specificity of FDG-PET(CT) in PCNSL was 87% (95% CI 83–90%) and 85% (95% CI 81–88%). The positive predictive value, negative predictive value, and diagnostic OR value were 84% (95% CI 81–88%), 87% (95% CI 84–90%), and 29.78 (95% CI 18.34–48.35), respectively. The results indicated that the diagnostic accuracy of pre-treatment PET-CT was high in immunocompetent PCNSL patients. Another diagnostic study (2010) showed that all PCNSL lesion with typical MRI findings exhibited strong FDG uptake in PET-CT. However, for atypical PCNSL patients, analysis of FDG uptake was insufficient to find intracranial lesions. Semiquantitative FDG uptake values (SUVmax) and quantitative FDG influx rate constants (*Ki*) in atypical lesion were significantly lower than those in typical MRI presentations. Only *K*_*3*_ value helped to differentiate PCNSL from inflammation or other tumors (typical vs atypical vs inflammation: 0.106 ± 0.032 min^−1^ vs 0.102 ± 0.030 min^−1^ vs 0.064 ± 0.014 min^−1^, *P* < 0.01) [33]. The combination of PET-CT and MRI will improve the accuracy of differentiation between PCNSL and GBM [34].

In February 2021, the International Primary CNS Lymphoma Collaborative Group (IPCG) published a consensus statement to recommend MRI and PET imaging for PCNSL patients, to improve the diagnosis and therapeutic response assessment [35]. A study comparing the efficiency of PET-CT and MRI during different time points showed that the presentations of PET-CT and MRI were consistent in 6 of 8 patients. During the long-term follow-up after treatment, 1 patient relapsed, whose re-occurrent lesion was identified 9 weeks earlier by PET-CT than by MRI imaging [36].

## Cognition function assessment

Recommendation: We suggest that MMSE be used to assess cognitive function in the management of PCNSL patients (2B).

Both the tumor itself and treatment-related neurotoxicity can affect the cognitive function of PCNSL patients, resulting in decreased QoL and limited social capability [37]. Therefore, the management of PCNSL patients should fully consider the net clinical benefits of the treatment regimen, in which cognitive function assessment is necessary. Assessment of patients’ cognitive function and QoL is recommended with lifelong follow-up. In clinical practice, the definition of cognitive impairment is a test score ≥ 1.5 SD worse than the mean of a given test’s normative distribution, adjusted for age, sex and education [38]. It is recommended to screen and evaluate the basic cognitive function of the PCNSL patient using the mini-mental status examination (MMSE), which is the most used screening test for cognitive function, given its simplicity and ease of performance. Other comprehensive cognition test sets, including the evaluation of memory, attention, executive function and visuospatial ability, are recommended as appropriate [39].

PCNSL patients undergoing clinical trials with cognitive impairment should have a detailed assessment of cognitive function using a neurocognitive test set before, during and after treatment. Generally, a neurocognitive assessment set consists of seven standardized tests, covering multiple areas sensitive to disease and treatment response (e.g., attention/executive function, language memory/naming, visuospatial ability) as well as a QoL questionnaire. Each specific aspect of cognitive function can be addressed with one to two tests, with testing time of 30 to 60 min in total [40].

A systematic review [37] published after 2018 were updated. Forty-two studies were eligible in our systematic review, including 4 randomized controlled trials (RCTs) and 38 cohort studies (*n* = 1990) (Additional file 3: CQ 4). Compared to the baseline status, the cognitive function and health-related quality of life (HRQoL) of PCNSL patients improved after induction chemotherapy. Similar effects were observed in patients treated with immunochemotherapy or intravenous and intrathecal chemotherapy. The effect of radiotherapy on cognitive function was ambiguous based on recent studies. Dose-reduced WBRT did not seem to have a negative effect on cognitive functioning while standard-dose WBRT did.

There was a RCT published in 2019 evaluating the cognitive function of PCNSL patients who were treated with WBRT or ASCT as consolidation therapy [41]. It showed that more than half of the patients who received WBRT had a decline in cognitive function, while the patients who received ASCT had improved executive function during the follow-up. Another clinical trial published in 2021 compared the cognitive functioning endpoint in patients treated with dose-reduced radiotherapy, chemotherapy and rituximab [42]. Comparing baseline status to shortly after radiotherapy, the test score in all the neurocognitive areas had a statistical improvement after 2 years; however, only the motion speed improved clinically and the mean score was still lower than the normal population. It was noteworthy that scores on neurocognitive domain remained stable for 2 years of follow-up.

Considering the recommendations of IPCG and BSH (2018) as well as the availability of neurocognitive tests in China [38], we summarized the cognitive function test set which is appropriate for PCNSL patients in China (Table 3). The recommended screening tools of cognitive function test are MMSE and Montreal Cognitive Assessment (MoCA) [43], and EORTC QoL questionnaire is recommended for evaluating patients’ QoL [44]. A systematic and comprehensive assessment toolset should cover each sub-cognitive area of cognitive function, including language memory (language learning test—Huashan revised version), attention (Stroop test, Digit span), executive ability (trail making test), visuospatial ability (clock-drawing, Rey–Osterrieth complex figure test), etc.

**Table 3:** Recommendations of neurocognitive assessment for PCNSL patients at baseline and follow-up

Cognitive area	Recommended tests [37, 38]	Tests suitable for patients in China	Time (min)
General	MMSE	MMSE	5–10
		MoCA	5–10
Premorbid IQ estimation	Barona index	Wechsler Adult Intelligence Scale	5
Attention/executive	Digits forward and backward span; trail making test (Parts A and B)	Digit span	5
		Trail making test (A, B)	5–10
		Stroop test	5–10
Verbal memory	Hopkins verbal learning test-revised	AVLT-Huashan	10
Word fluency	Not mentioned	BNT	5
		Animal verbal fluency test	1
Motor	Grooved pegboard test	Grooved Pegboard test	15–20
Visuospatial ability	Not mentioned	Clock-drawing test	5
		Rey–Osterrieth complex figure test	15–40
QoL	EORTC-QLQ-C30	EORTC QoL questionnaire	10
	BCM 20	Evaluation of brain function in cancer treatment	10–15

There were two systematic reviews identifying cognitive domains and tests to be assessed for PCNSL. One was conducted by Correa et al. [38], and the other was by van der Meulen et al. [37]. The evidence evaluation group adopted and combined their results as “Recommended tests.” Time: duration needs to be taken for each test. MMSE: mini-mental state examination. MoCA: Montreal Cognitive Assessment

*IQ* Intelligence Quotient, *AVLT* Huashan: Auditory Verbal Learning Tests—Huashan version, *BNT* Boston naming test, *QoL* quality of life

## Induction therapy

### Regimens

Recommendation: We recommended that newly diagnosed PCNSL patients should be treated with a combined HD-MTX-based regimen, if the patient is fit for chemotherapy (1B). The combined therapeutics can be rituximab (2C), cytarabine (2B), TMZ (2C), or other drugs which can cross the BBB (2C).

MTX is the therapeutic backbone in the treatment of PCNSL [45]. The dose of intravenous MTX that penetrating the BBB is generally 1–8 g/m^2^ with a cytotoxic concentration in the CSF of > 3 g/m [2, 46]. Therefore, 3–3.5 g/m^2^ MTX is usually well tolerated and effective in patients with adequate hydration and alkalization when followed by leucovorin rescue [47]. Rapid infusion (2–3 h) can result in better therapeutic concentrations in CSF [48], and an expert group in China recommended an infusion duration of 3–4 hours [49]. Most RCTs conducted induction therapy every 3–4 weeks for 4–6 cycles, but no consensus has been reached on the optimal number of cycles. Specifically, ENOS recommends the intravenous infusion of HD-MTX over 2–3 h, with induction therapy consisting of 2–3 weeks/cycle for at least 4–6 cycles (a good practice point) [8]. However, if patients cannot tolerate HD-MTX-based chemotherapy, radiotherapy can be considered as an alternative treatment [50, 51]. The CSCO guidelines also recommend WBRT as the optimal choice for induction therapy when the patient is unfit for systemic chemotherapy [11].

A variety of combinations have been reported in previous studies, including HD-MTX with rituximab, TMZ, ifosfamide, HD-cytarabine, thiotepa (T), carmustine, teniposide, procarbazine, and vincristine. Regimens including cytarabine or rituximab with MTX showed benefit on remission rates. Compared to dual or triple therapy, four- or five-drug regimens have achieved higher remission rates [52].

A systematic review was conducted with 16 eligible RCTs (11 studies were conducted in China. Additional file 3: CQ 5). The meta-analysis showed that ORR of the dual, triple, four- or five-drug regimens were about 0.7, 0.79 and 0.85, respectively. Compared to the regimens without rituximab, the addition of rituximab to MTX-based chemotherapy had higher ORR (RR = 1.31, 95% CI 1.20–1.43, *P* < 0.001) and improved PFS (HR = 0.84, 95% CI 0.73–0.96, *P* = 0.009), but no significant difference in OS was observed (HR = 0.89, 95% CI 0.78–1.02, *P* = 0.09). Similarly, regimens with HD-cytarabine and HD-MTX achieved higher CR rates (RR = 1.66, 95% CI 1.19–2.32, *P* = 0.003), but no significant difference in PR, OS, and PFS compared with single HD-MTX regimen. However, a contradictory result was seen in the RCT evaluating temozolomide. A study by Omuro et al. showed that in older patients, the median OS of MTX + P + vincristine + cytarabine was significantly higher than that of MTX + TMZ (31 m vs. 14 m, 95% CI 12.2–35.8 vs. 95% CI 8.1–28.4) [53]. Another study showed that ORR of MTX + TMZ was higher than the control group treated with MTX + WBRT (83.3% vs. 54.2%, *χ*^2^ = 4.752, *P* < 0.05) [54].

The toxicity of MTX-containing regimens should be carefully considered, especially for older, fragile patients with a history of kidney disease, and a full evaluation is needed pre-treatment. Adequate hydration, alkalization, leucovorin rescue and monitoring of serum MTX concentration are required before and after HD-MTX infusion. Patients considered for MTX regimes should have a minimum of 2000–3000 mL/m^2^ urine volume per day [55], and HD-MTX should not be used in patients with a creatinine clearance of < 30 ml/min. At present, there is no generally accepted guideline for the MTX dose reduction method according to baseline renal function, and dose reduction schemes have not been agreed upon [56]. Monitoring the serum MTX concentration 24, 48 and 72 h after the administration of HD-MTX will facilitate the early detection of delayed MTX clearance.

Another HD-MTX-associated toxicity is acute liver injury, which usually occurs 1–3 days after administration, and is usually manifested by transient aminotransferase elevation. The following cycle of HD-MTX at adjusted dose should not be administered until both aminotransferases and bilirubin return to normal levels. In particular, the administration of HD-MTX in patients with liver cirrhosis should be carefully implemented with caution [49].

Treatment-related adverse events (AEs) were more common in MTX-based four-drug regimens than with dual/triple regimens. About 60% of patients had grade 3–4 AEs after combined chemotherapy, with infection as the major cause of mortality [52, 57, 58]. Neutropenia, thrombocytopenia and anemia were common with the combined regimen of HD-cytarabine and HD-MTX [57–59], and the severity of hematologic AEs increased to grade 3–4 in 72% of patients with a four-drug regimen [53].

For older PCNSL patients, a Chinese experts consensus (2019) suggested that HD-MTX should not be considered as routine therapy for patients 50–60 years old, and should not be used in patients ≥ 60 years [49]. However, British experts suggested that the feasibility of HD-MTX treatment should be determined by the patients’ PS, and that age alone should not be a factor in the treatment decision [9]. In addition, some studies indicate that older PCNSL patients can benefit from HD-MTX treatment [60]. As PCNSL is a disease that occurs in middle-aged and older adults, a comprehensive geriatric assessment helps to assess the patient’s overall condition and thus facilitates an appropriate individualized treatment plan.

### Rituximab

Recommendation: We suggest that newly diagnosed PCNS-DLBCL patients can be treated with a rituximab-inclusive regimen in induction therapy (2C).

Rituximab, a targeted CD20 antibody, is a standard component for treating mature B cell NHL, with significant improvement in PFS and OS of B cell NHL [61]. The NCCN (2021), CSCO (2021), and BSH (2018) guidelines all include rituximab in combination with HD-MTX as an induction regimen. Rituximab combined with chemotherapy can significantly increase the remission rate and the PFS of newly diagnosed patients without elevating risk of SAEs. However, studies did not show an OS benefit. This supports early application of rituximab in newly diagnosed patients, likely because the destruction of the BBB by lymphoma infiltration enhances the penetrating capability of rituximab [62]. Therefore, for patients with newly diagnosed PCNSL, rituximab (375 mg/m^2^) is recommended as a first-line induction therapy combined with HD-MTX. However, given that rituximab did not show benefit on overall survival, the patient’s economic status should be considered as well.

A systematic review was conducted with 13 studies included (2 RCTs and 11 retrospective cohort studies, *n* = 1222. Additional file 3, CQ 6). The results showed that compared with the regimens without rituximab, the PCNSL patients treated with rituximab (375 mg/m^2^ in 9 studies and 500 mg/m^2^ in 3 studies) had statistically significant higher ORR (RR = 1.22, 95% CI 1.12–1.32, *P* < 0.001), CR rate (RR = 1.34, 95% CI 1.18–1.51, *P* < 0.001) and PFS (HR = 0.73, 95% CI 0.61–0.88, *P* = 0.001). However, there was no significant difference in OS (HR = 0.82, 95% CI 0.67–1.01, *P* = 0.06). Regarding the safety of rituximab in the treatment of PCNSL, no significant difference in AEs was seen in our analysis such as neutropenia, thrombocytopenia, anemia, hepatotoxicity (RR = 1.01, 95% CI 0.69–1.47, *P* = 0.98), and nephrotoxicity (RR = 1.42, 95% CI 0.60–3.39, *P* = 0.42).

## Consolidation therapy

Recommendation: Compared with non-reduced dose WBRT, we suggest that ASCT can be used as consolidation therapy for PCNSL patients who are fit for conditioning chemotherapy (2C).

For the patients achieving CR or CR unconfirmed (CRu), consolidation therapy including myeloablative chemotherapy with ASCT, WBRT, and non-myeloablative regimens are able to improve the treatment response and patients’ survival [63]. Among them, WBRT was the consolidation therapy routinely used in patients with PCNSL [64, 65]. However, the risk of delayed neurotoxicity and decline in neurocognitive function and QoL limit its wide application [65, 66]. Instead, ASCT has been accepted as a novel consolidation therapy for PCNSL patients younger than 70 years with adequate organ function and favorable performance in the last 10 years [67–69]. A thiotepa-based conditioning regimen is recommended, in view of its superior benefit on patients’ neurological function and QoL, and equivalent therapeutic response compared with non-reduced dose (≥ 36 Gy) WBRT. However, attention should be paid to the high incidence of hematological AEs with ASCT and the possibility of treatment-related mortality. The cost and patient’s economic status should also be considered.

A systematic review was conducted with 6 studies (2 RCTs, 1 non-randomized controlled study, 1 cohort study, 2 case series studies, *n* = 535. Additional file 3: CQ 7). The result of meta-analysis showed that as consolidation therapy, ASCT and non-reduced dose WBRT had similar ORR, 2-yearS PFS and 2-years OS. The patients treated with ASCT had significantly higher grade 3/4 AEs of neutropenia (RR = 13.28, 95% CI 5.16–34.15, *P* < 0.001), thrombocytopenia (RR = 26.55, 95% CI 6.81–103.59, *P* < 0.001), anemia (RR = 9.01, 95% CI 2.20–36.88, *P* = 0.002), fever (RR = 14.22, 95% CI 1.94–104.09, *P* = 0.009), gastrointestinal AEs (RR = 21.83, 95% CI 1.32–361.77, *P* = 0.03), and mucositis (RR = 29.42, 95% CI 1.80–480.16, *P* = 0.02). No significant differences were seen in liver, kidney and heart injury, acute neurotoxicity and treatment-related mortality.

IELSG32 trial compared neuropsychological function between 30 patients treated with WBRT and 27 patients with ASCT in 2017 [70]. The analysis was divided into early and late effects relating to shortly after and 2 years after consolidation therapy, respectively. Both consolidation methods showed rapid improvement in neuropsychological function. In particular, the ASCT group had significant improvement in visual construction ability (Rey Complex Figure Copy), attention and executive function (Trail Making Test A, Trail Making Test B, Trail Making Test B-A, phoneme language fluency) immediately after ASCT, and long-term memory (Rey Audit Verbal Learning Test-Delayed Recall) in 2-year follow-up. Significant impairment was seen in attention and executive function of WBRT group by Wisconsin Card Sorting Test. In 2019, the PERCIS study [41] showed that in the PCNSL patients ≤ 60 years, ASCT tended to be the favored choice of consolidation therapy. More than half of the patients treated with ASCT exhibited improved neuropsychological scores while more than half of the patients with WBRT had a lower score of the tests.

Based on the limited number of studies, dose-reduced WBRT seems to be less toxic than non-reduced dose WBRT in terms of neurologic functional impairment [71, 72], and demonstrated a similar response to ASCT [73]. Currently, up to 36 Gy of WBRT with a boost to gross disease to a total of 45 Gy is recommended to the patients who achieve less than CR in systemic induction therapy [1, 74].Therefore, for PCNSL patients who get CR/CRu but cannot tolerate ASCT treatment, dose-reduced WBRT (< 23.4 Gy) can be considered as one of the consolidation therapeutic strategies, given the balance of neurotoxicity and treatment effectiveness [74]. However, there is no study directly comparing dose-reduced WBRT and ASCT on treatment effect and AEs.

As a disease commonly occurred in middle-aged and elderly patients, a certain number of patients with PCNSL are unable to tolerate myeloablative chemotherapy or not accepting the risk of WBRT-related neurotoxicity. Non-myeloablative regimens such as HD-cytarabine, etoposide, or routine HD-MTX treatment can be the alternative choice of consolidation therapy for those patients [75–77]. No clear consensus exists on the optimal choice between myeloablative and non-myeloablative consolidation [10]. Well-designed age-stratified studies on consolidation strategy will help to provide more evidences for clinical decision.

## Treatment for relapsed/refractory patients

There is no standard regimen particularly preferred for relapsed/refractory (R/R) PCNSL. Therapeutic regimen can be chosen depending on the remission duration and prior treatment. For the patients whose remission is longer than 1 year, re-treating with HD-MTX-based therapy can be applied. For those who is refractory to MTX or relapsed < 1 year, systemic therapy of Bruton’s tyrosine kinase (BTK) inhibitor such as ibrutinib, WBRT, high-dose chemotherapy with stem cell rescue can be the alternative choices.

### Bruton’s tyrosine kinase inhibitor (BTKi)

Recommendation: We suggest that refractory or relapsed PCNSL patients can be treated with ibrutinib with or without high-dose chemotherapy as re-induction therapy (2C).

Abnormal B cell receptor signaling transmission is essential to the development and progression of B cell malignancies and Bruton’s tyrosine kinase (BTK) plays a critical role. BTK inhibitors (BTKi) are a novel medicine which has shown great advantages in treating mature B cell malignancies and autoimmune diseases [78]. Clinical guidance is, therefore, needed regarding BTKi in the treatment of CNS lymphomas.

There are three BTK inhibitors currently available in China: ibrutinib, orelabrutinib, and zanubrutinib. The CSF/plasma ratio of ibrutinib was reported as 28.7% after correction for protein binding rate [79]. Orelabrutinib has also shown good CSF distribution in patients with recurrent and refractory PCNSL at conventional therapeutic dose [80]. In published studies, ibrutinib was the most frequently studied BTKi in refractory/relapse PCNSL patients at the dose of 560–840 mg per day [81, 82]. However, the optimal dosage of ibrutinib in CNS lymphoma treatment has not been determined. For approved indications [83], 560 mg/day of ibrutinib is recommended for treating CNS lymphoma.

A systematic review was conducted by including 18 studies (1 cohort study, 7 case reports/case series, 1 non-randomized controlled study, and 9 conference abstracts, *n* = 242. Additional file 3, CQ 8). It showed that ORR of ibrutinib-inclusive regimen was 74% (95% CI 66–83%), suggesting that ibrutinib is effective to treat PCNSL patients. The rates of CR and PR of ibrutinib-based therapy were 47% (95% CI 34–59%) and 26% (95% CI 17–35%), respectively. In detail, the ORR of ibrutinib monotherapy or combined regimens was 66% (95% CI 53–79%) and 86% (95% CI 79–93%), CR rate was 26% (95% CI 16–35%) and 54% (95% CI 40–68%), respectively. The majority of AEs were hematological toxicities, which occurred more with combined ibrutinib and chemotherapy [82, 84]. Only one non-randomized study has been reported for another BTKi, tirabrutinib in the treatment of refractory/relapsed (R/R) PCNSL (*n* = 44) [85]. 64% (28/44) of the patients achieved a response, with CR and PR rates of 34% and 29.5%, respectively, with a median PFS of 2.9 months. We did not include this study in this consensus because tirabrutinib has not been approved in China. As for two other BTKi currently available in China, 23 and 16 CNSL patients treated with orelabrutinib or zanubrutinib, respectively, were reported in a retrospective study, or in case series. The outcomes of these BTKi for PCNSL need to be followed-up [86–88].

Another systematic review evaluated the effect of ibrutinib in DLBCL, with 13 studies being included (1 RCT, 8 single-arm clinical trials, 2 retrospective studies, and 2 ongoing clinical trials, *n *= 1445) [89]. It showed that the median PFS and OS of the ibrutinib-treated patients were 4.54 months and 12.7 months, and the rates of overall response, CR, and PR were 57.9%, 35.0%, and 20.1%, respectively. These data showed that ibrutinib can improve the treatment response of the non-GCB DLBCLs and R/R CNS lymphomas, which support our conclusion regarding its activity in this disease.

It is important to note that BTKi have AEs including infection, hemorrhage, diarrhea, atrial fibrillation and skin rash, which result from inhibition of B cell function and off-targeted effects by binding to unrelated targets. Remission duration may be short if only a BTKi was used in induction therapy. Since orelabrutinib [90] and zanubrutinib [91, 92] have lessened the off-target effects, it is expected that further evidence on the application of BTKi in PCNSL patients will be available in future.

### Stereotactic radiosurgery

Recommendation:We suggest that stereotactic radiosurgery can be used for PCNSL patients with a limited recurrent lesion who were refractory to chemotherapy and have previously received WBRT (2D).

WBRT used to be a standard treatment for PCNSL before HD-MTX was widely accepted as the chemotherapy backbone. However, the benefit of salvage WBRT has been reevaluated in terms of the cost of neurocognitive damage. For patients with recurrent or chemotherapy refractory lesions who previously received WBRT, the tolerable dose of repeat radiotherapy to the whole brain is limited and may cause severe AEs. Stereotactic radiosurgery (SRS) is a type of radiotherapy with a focused dose for relatively limited lesions, thus reducing the radiation intensity on surrounding normal tissues and alleviating the adverse reactions of repeat radiotherapy. Several clinical studies with small a sample size showed that SRS, including fractioned stereotactic radiotherapy and single SRS, can achieve better clinical efficacy and less toxicity in refractory, localized PCNSL. However, most of these studies were case series or case reports, lacking a controlled comparison. Attention should be paid to how localized lesions are defined: In the published literature, these are generally defined as no more than two lesions with a total volume of less than 4 cm [3, 93–97].

A systematic review was conducted by including 5 studies (3 case series and 2 case reports, *n* = 70. Additional file 3, CQ 9) [93–97], showing that for localized recurrent or refractory PCNSL, 78.6–88.9% of the patients achieved response (CR+PR) after SRS, while 11.1% remained stable or progress. The median PFS was 3–32.1 months, and the median OS was 7.7–15 months. No obvious radiation-related toxicity was observed. Among those patients whose median tumor volumes were 3.5–6.7 cm^3^, those with tumors > 4 cm^3^ had shorter OS.

## Primary vitreoretinal lymphoma (PVRL)

### Diagnostic approach

Recommendation: We suggest that patients with suspected PVRL should be diagnosed by vitreous biopsy (2D).

The diagnosis of PVRL requires the definitive identification of malignant lymphoid cells in the eye [98]. Methods for obtaining intraocular specimens include anterior chamber paracentesis, diagnostic vitreous biopsy, retinal biopsy, chorioretinal biopsy, and eyeball enucleation. The most common approach is anterior chamber paracentesis and vitreous biopsy [99]. Anterior chamber paracentesis costs less than vitreous biopsy and is easier to perform. However, some PVRL patients exhibit no parenchymal lesions and lymphoma cells easily degenerate [100], while vitrectomy enables multiple vitreous specimens for cytopathological detection which helps to ensure an accurate diagnosis [101]. All the histologic techniques can increase the detection efficiency to assist in PVRL diagnosis. In addition, vitrectomy has a certain therapeutic effect on PVRL [102]. There has been no well-performed diagnostic study comparing these two procedures. Thus, no evidence indicated that diagnostic vitreous biopsy is superior to aqueous humor puncture at present.

We conducted a meta-analysis including 5 studies in total (1 cohort study and 4 case series studies. Additional file 3: CQ 10). In 65 paired aqueous vs vitreous samples from patients diagnosed as VRL, 8 aqueous samples were found VRL positive (0–66.7%) and 9 vitreous samples were found VRL positive (1.9–66.7%) [103, 104]. In the unpaired samples of VRL, 13 of 33 aqueous samples were VRL positive (20.0–66.7%) and 27 of 38 vitreous samples were VRL positive (44.4–80.0%) [104–106]. In another study of 167 suspected uveitis patients, 47 of 51 vitreous samples were VRL positive by cytological and flow cytometric analysis and 40 of 45 aqueous samples were tested VRL positive by IL-10 detection (≥ 50 pg/ml was regarded as positive with sensitivity of 89% and specificity of 93%) [107]. In the above studies, some aqueous samples were not cytologically confirmed due to inadequate sample size, resulting in the uncertainty of VRL diagnosis, because cytokine detection was just an indirect indicator of lymphoma infiltration. The diagnostic efficiency of aqueous puncture needs further verification.

### Treatment

Recommendation: We suggest that PVRL patients or PCNSL patients with concurrent VRL can be treated with combined systemic and local therapy (2C).

Traditional therapy for PVRL patients or PCNSL with intraocular involvement included systemic chemotherapy, WBRT and ASCT [98]. In recent years, local treatment including intraocular chemotherapy and ocular radiotherapy has been more widely used in practice [108–110]. Either systemic or local treatment can alleviate the symptoms and intraocular tumors [109, 111–114]. However, outcomes including PFS, failure-free survival (FFS), OS, CNS/intraocular recurrence vary across studies. Some studies showed that a combination of systemic and local treatment could prolong PFS and FFS but does not affect OS [115–118]. Therefore, in patients with PVRL and involvement of both eyes, and patients with PCNSL and concurrent VRL, systemic treatment should be combined with local treatment. The affected eye(s) should be examined regularly to monitor the ocular toxicities caused by chemotherapy or radiotherapy, and local treatment should be suspended in case of severe ocular toxicity. For patients with unilateral PVRL, unilateral local treatment can be considered. However, as steroid treatment is often used prior to biopsy to relieve symptoms in VRL, it is difficult to exclude false negative pathology results. Caution needs to be taken to make the diagnosis of unilateral PVRL if patients have symptoms in both eyes (statement of good practice).

A systematic review was conducted with 7 studies (1 single-arm clinical trial, 6 cohort studies, *n* = 503. Additional file 3: CQ 11). A cohort study analyzed the outcome of systemic therapy, local therapy and combined systemic and local therapy in the patients with PVRL or concurrent VRL (*n* = 69) [115]. It showed that the combined approach was associated with better FFS (*P *= 0.002) and CNS recurrence-free survival (CNS-RFS, *P *= 0.003). In addition, ASCT showed a survival benefit with better OS in this study, indicating that intensive systemic treatment may improve the prognosis of the patients with PVRL or PCNSL and concurrent VRL. Another retrospective study (*n* = 221) indicated that adjuvant local treatment with systemic strategy is an independent predictor of PFS (HR = 1.69, 95% CI 1.12–2.54, *P* = 0.01) but not affects OS. Multivariate analysis showed that the patient’s age was an independent predictor of OS (HR = 1.04, 95% CI 1.02–1.05, *P* < 0.0005) [118].

Regarding the benefit of local treatment versus systemic treatment, a retrospective cohort study (*n *= 22) showed that the median PFS was 5.5 months and 12 months, respectively, suggesting that systemic treatment helped to prolong the PFS [119]. Combined systemic and local treatment was also superior to local strategy on patients’ PFS or CNS lymphoma-free survival [116, 117].

However, a couple of retrospective studies didn’t show the association between patients’ outcome and treatment type [120, 121]. Interestingly, both studies were based on multicenter collaboration. One was a report from International Primary Central Nervous System Lymphoma Collaborative Group. The patients were diagnosed not only with large B cells but also T cells or not specified, and WBRT and systemic therapy were grouped together in comparison with local treatment [120]. The other was a 17-center European study on PVR-DLBCLs [121]. In this study, the systemic treatment included CHOP-exclusive chemotherapy, ASCT and WBRT. Thus, inaccurate diagnosis and inconsistent therapy strategy may result in an uncertain conclusion.

## Flowchart of diagnosis and treatment

See Figs. 1 and 2.

**Fig. 1:**
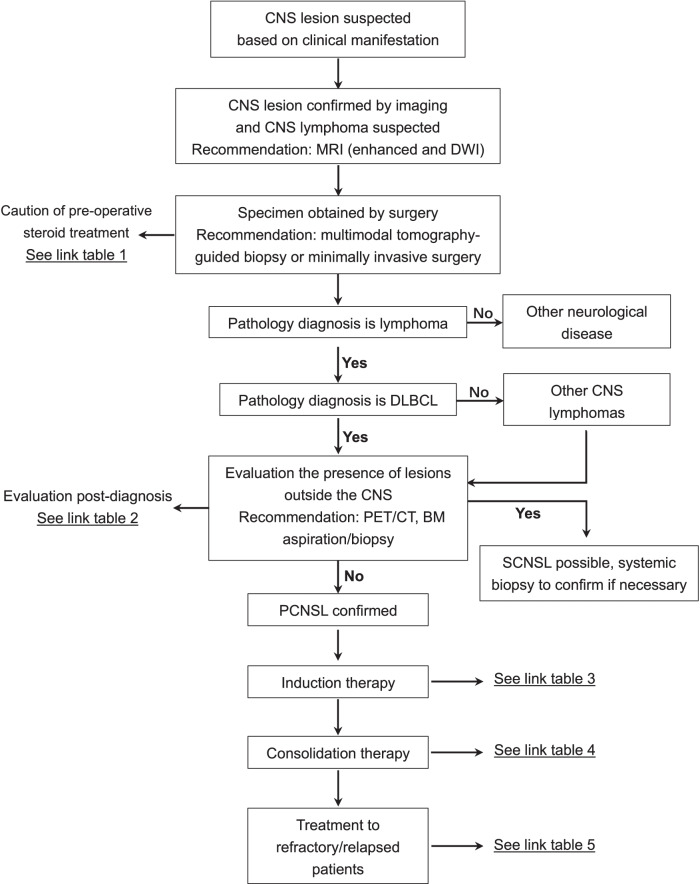
Flowchart of diagnosis and treatment. Common procedure of diagnosis, evaluation, and therapeutic regimens for newly diagnosed and relapsed/refractory PCNSL patients

**Fig. 2:**
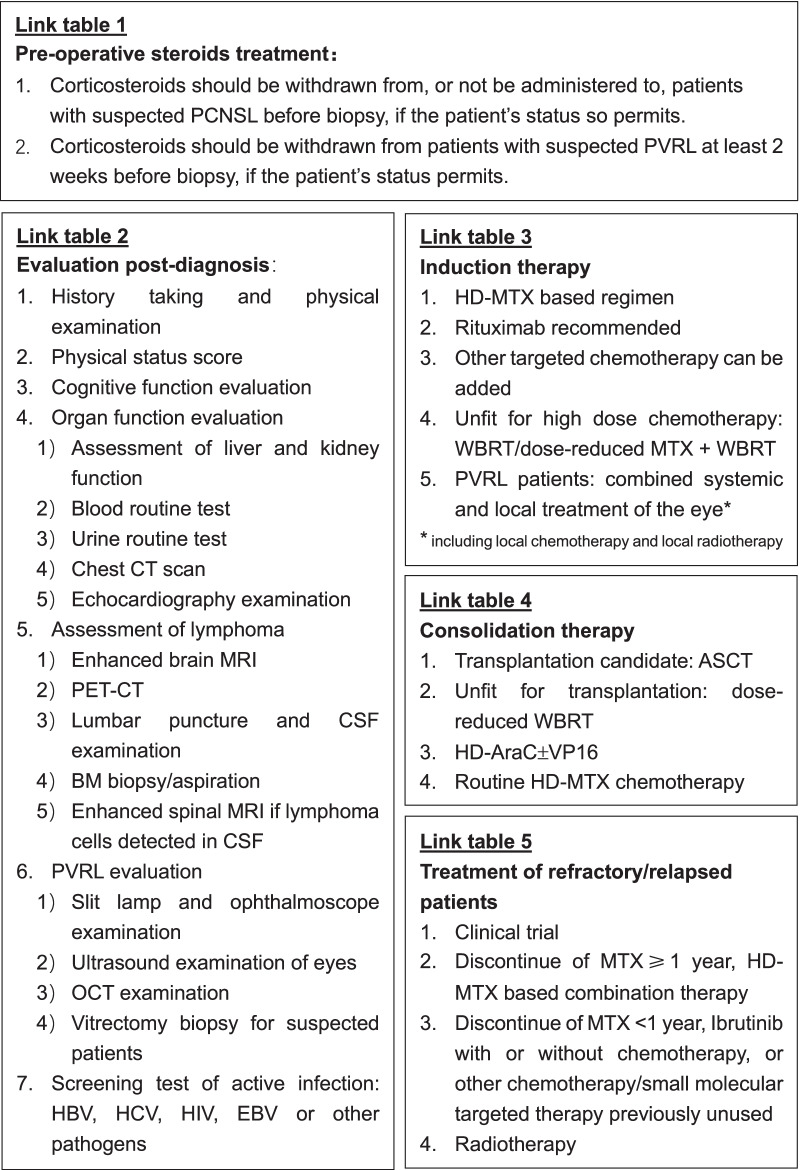
Link tables for the management of PCNSL. Additional information of preoperative steroids treatment, evaluation post-diagnosis, induction therapy, consolidation therapy, and treatment for relapsed/refractory patients

## Suggestion for future research

### Application of immune cell therapy

Chimeric antigen receptor (CAR)-T cells as a “living drug” are able to penetrate BBB [122] and have been successfully used in treating R/R DLBCL [123, 124]. The application of CAR-T cells in PCNSL remained debating because infusion of CAR-T cells may cause severe neurotoxicity, which is resulted from intracranial monocyte/macrophage-mediated cytokine release and off-tumor targeted BBB damage [125, 126].

The occurrence of CAR-T cell-related encephalopathy syndrome (CRES), which is also termed as immune cell-associated neurotoxicity syndrome (ICANS) can be biphasic, where the first phase is concurrently with cytokine release syndrome (CRS) within 5 days of cell infusion and the second is beyond. In some patients, a delayed CNS dysfunction can be observed during 4–5 weeks after CAR-T transfusion [127]. Patients undergoing CRES may exhibit various CNS symptoms such as headache, delirium, anxiety, tremor, aphasia, decreased level of consciousness and cerebral edema [128]. The grading tools including CARTOX-10, modified CAR-T related encephalopathy syndrome (mCRES) and American Society for Transplantation and Cellular Therapy (ASTCT) scales were reported to be superior to the National Cancer Institute Common Terminology Criteria for Adverse Events (CTCAE) in evaluating the severity of CRES [127, 129].

Recently, some studies indicated that CD19CAR-T therapy had effective outcome in refractory CNSL patients with the controllable ICANS [130–133]. A prospective phase I/II study was conducted to treat R/R PCNSL patients with tisagenlecleucel (tisa-cel) at the FDA-approved dose of (0.6–6.0) × 10^8^ CAR-T cells. With a medium time of 12.2-month follow-up, 7/12 patients were observed response of 6 CRs and 1 partial remission (PR). No tocilizumab was required even though seven patients experienced grade 1 CRS at the medium onset of 4 days after infusion. Grade 1–3 ICANS were observed in 3, 2, and 1 patients, respectively, all of whom received at least one dose of dexamethasone [131]. A retrospective study reported a cohort treating R/R PCNSL patients with CAR-T cells, of which 7 patients received tisa-cel and 2 received axicabtagene ciloleucel (axi-cel). Five of nine patients (2 axi-cel, 3 tisa-cel) were observed CR and 1/9 patient (tisa-cel) was PR at best response. Medium onsets of CRS and ICANS after infusion were 5 and 8 days, respectively. ICANS ≥ grade 3 occurred in 2/9 patients (1 axi-cel, 1 tisa-cel) [130]. In another retrospective cohort enrolling patients with CD19^+^ R/R NHL and chronic lymphocytic leukemia, a CAR construct containing a CD28 costimulatory domain and coexpressing truncated epidermal growth factor receptor was used to generate CD19CAR-T cells. Five patients were diagnosed as PCNSL. Among them, 5/5 patients occurred Grade > 1 CRS or CRES, 2 of whom received tocilizumab or dexamethasone treatment [133]. No treatment-related mortality was reported in the above studies [130, 131, 133].

With a view of balancing therapeutic benefit and side effect, CD19CAR-T cells have been showed as a promising tool to treat R/R PCNSL. The optimal infusion timing, dose, and prophylactic measures to ICNAS need to be determined in future.

### Application of molecular diagnostic techniques

The typing system including morphology, immunology, cytogenetics, and molecular biology (MICM) has been widely applied in a variety of hematopoietic malignancies for the diagnosis, prognosis, and response evaluation. However, in the field of PCNSL, the application of MICM in both diagnosis and prognosis lags far behind other diseases. Research is needed to guide the optimal use of novel techniques such as gene mapping, PET-MRI, and circulating tumor DNA (ctDNA) detection of cerebrospinal fluid (CSF) in the diagnosis, prognosis, and response evaluation.

### Application of small molecular targeted medicine

In recent years, the application of a variety of small molecular targeted medicine has significantly improved the survival and prognosis of lymphoma patients, especially those with in B cell lymphoma. However, only a few studies focused on PCNSL, in particular newly diagnosed patients. Well-designed RCTs examining targeted therapies combined with chemotherapy are needed.

### Dose-reduced WBRT versus ASCT in the consolidation therapy

Standard-dose WBRT has been replaced by dose-reduced WBRT due to radiotherapy-related side effects on the central nervous system. In this expert consensus, we demonstrated that standard-dose WBRT is inferior to ASCT in consolidation, in terms of neurological function. However, no studies have compared the curative potential and the safety between dose-reduced WBRT and ASCT as a consolidation therapy. Clinical research is needed to address this issue.

### Maintenance therapy

PCNSL is a special subtype of DLBCL, and therefore, the treatment principles for systemic DLBCL should not be adopted in their entirety. Currently, maintenance therapy is not recommended for DLBCL patients who obtain a CR after 4–6 cycles of an R-CHOP induction regimen. However, the prognosis of PCNSL is far worse than that of systemic DLBCL. The question as to whether and which maintenance therapies have benefit for PCNSL patients remains to be addressed.

### Real-world evidence of PCNSL

Due to our population base, the absolute number of PCNSL cases in China is much larger than other countries [76, 134]. However, few clinical studies have been performed in China. Real-world multicenter studies on the incidence, diagnosis, treatment, and response evaluation from a large cohort of PCNSL patients in China will help to inform future clinical practice recommendations.

## Conclusions

This consensus is intended for use by hematologists, neurosurgeons, oncologists, neurologists, ophthalmologists, radiologists, pathologists, diagnostic imaging physicians, clinical pharmacists, and other professionals involved in the diagnosis, treatment, and management of PCNSL. The target audience of the consensus is patients with PCNSL. As PCNSL is a rare extra-nodal NHL restricted to the CNS, the consensus will fill the gaps in clinical practice and help to build the collaborative philosophy in multicenter clinical researches. The cutting-edge progress will be updated periodically.

## Supplementary Information

**Additional file 1**. Selection of clinical questions.

**Additional file 2**. Search strategies in each database.

**Additional file 3**. Supplementary data for focused clinical questions.

**Additional file 4**. Delphi results.

## Abbreviations

AE: Adverse effect
ASCT: Autologous hematopoietic stem cell transplantation
BBB: Blood–brain barrier
BTKi: Bruton’s tyrosine kinase inhibitor
CAR-T: Chimeric antigen receptor T cells
CHOP: Cyclophosphamide, doxorubicin, vincristine, prednisone
CQ: Clinical question
CR: Complete remission
DLBCL: Diffuse large B cell lymphoma
HD-MTX: High-dose methotrexate
MMSE: Mini-mental status examinations
NCCN: National comprehensive cancer network
ORR: Overall response rate
OS: Overall survival
PCNSL: Primary central nervous system lymphoma
PFS: Progression-free survival
PR: Partial remission
PVRL: Primary vitreoretinal lymphoma
RCT: Randomized control trial
SRS: Stereotactic radiosurgery
WBRT: Whole-brain radiotherapy

## Declarations

### Availability of data and materials

All information in this consensus can be found in the additional files and references.
