# Supplementary material for: Evidence-based expert consensus on the management of primary central nervous system lymphoma in China
Source: J Hematol Oncol. 2022 Sep 29;15:136. doi: 10.1186/s13045-022-01356-7 (PMC9524012; doi:10.1186/s13045-022-01356-7)
Supplement: Supplementary file 1 — Additional file 1. Selection of clinical questions. [file 13045_2022_1356_MOESM1_ESM.pdf]

Additional file 1. Selection of Clinical Questions

The secretariat initially identified 25 Clinical Questions (CQs) based on a systematic search of published guidelines, systematic reviews on PCNSL, and virtual interviews with several hematologists, neurosurgeons, and ophthalmologists. The CQs were related to diagnosis (n=5), treatment (n=15), prognosis (n=2), other (n=3). The expert consensus group members were invited to rate the importance of the proposed CQs via an email questionnaire on 9 February 2021, where the importance of each CQ was evaluated from not important (1) - very important (7). The questionnaire was returned by 17 February 2021. The average scores of each CQ at first round questionnaire were listed in Supplementary Table S1.1.

The CQs being rated >4 were collected in second round questionnaire for discussion. Among them, there were overlaps of clinical practice relevant between CQ#1 and CQ#3, CQ#15 and CQ#16, CQ#17 and CQ#18, CQ#19 and CQ#20. The overlapped CQs were combined as one CQ and reordered following the practice procedure of diagnosis-staging and evaluation-treatment (Supplementary Table S1.2). A total 11 CQs were finally developed, and the training course of systematic review and evidence evaluation were processed afterwards.

Supplementary Table S1.1. Clinical questions of first round questionnaire

1st round CQ number	1st round clinical questions	Survey score
1	Which is the preferred approach to obtain the pathology specimen in patients with PCNSL, stereotactic brain biopsy or resection?	5.91
3	Should biopsy be performed if PCNSL is suspected by enhanced MRI examination?	5.66
21	Which is the preferred imaging examination for PCNSL patients, MRI or whole-body PET-CT?	5.51
7	Which regimen is preferred to be combined with HD-MTX backbone in induction therapy?	5.48
15	Which is the preferred consolidation therapy for patients with PCNSL at consolidation therapy, whole-brain radiotherapy (WBRT) or autologous hematopoietic stem cell transplantation (ASCT)?	5.27
18	What is the preferred treatment to the first relapsed or multiple relapsed PCNSL patients?	5.25
8	Should rituximab be used to treat newly diagnosed PCNS-DLBCL patients in induction therapy?	5.10
19	Which is the preferred approach to treat patients with PIOL, radiotherapy or chemotherapy?	4.95
2	Should corticosteroids be withdrawn from patients with suspected PCNSL/PVRL before biopsy?	4.9
5	Which is the preferred approach to make the diagnosis of a suspected PVRL, vitreous biopsy or aqueous humor/vitreous puncture?	4.88
17	Should stereotactic radiosurgery be used to treat localized recurrent PCNSL patients who were refractory to chemotherapy and previously received WBRT?	4.84
24	Should cognitive function assessment be used for PCNSL patients?	4.72
20	What is the preferred dose and frequency of intraocular MTX injection in treating PIOL patients?	4.57
16	Which is the preferred approach to treat PCNSL patients with WBRT, reduced dose WBRT or non-reduced dose WBRT?	4.35
11	Should BTK inhibitors be used to treat patients with PCNSL?	4.23
14	Should intrathecal injection of MTX be used with HD-MTX backbone?	3.94
25	Should rehabilitation training be needed for PCNSL patients?	3.88
12	Should lenalidomide be used to treat patients with PCNSL?	3.84
4	Should CSF cytokines such as IL-10/IL-6, BAFF, APRIL be used as a diagnostic parameter of PCNSL?	3.82
23	Should neuropsychological assessment be used for PCNSL patients?	3.68
9	Should temozolomide be used to treat newly diagnosed PCNSL patients?	3.41
22	Should CSF cytokines such as IL-10/IL-6, BAFF, APRIL be used as an evaluation tool for PCNSL patients?	3.34

10	Should corticosteroids be included in the therapeutic regimen for PCNSL patients?	3.32
13	Should CAR-T cells be used to treat PCNSL patients?	2.95
6	Should operative resection be used to treat PCNSL patients?	2.93

Supplementary Table S1.2. Clinical questions of second round discussion

2nd round CQ number	2nd round clinical questions
1	Which is the preferred approach to obtain the pathology specimen in patients with PCNSL, stereotactic brain biopsy or resection?
2	Should corticosteroids be withdrawn from patients with suspected PCNSL/PVRL before biopsy?
3	Which is the preferred imaging examination for PCNSL patients, MRI or whole-body PET-CT?
4	Should cognitive function assessment be used for PCNSL patients?
5	Which regimen is preferred to be combined with HD-MTX backbone in induction therapy?
6	Should rituximab be used to treat newly diagnosed PCNS-DLBCL patients in induction therapy?
7	Which is the preferred consolidation therapy for patients with PCNSL at consolidation therapy, whole-brain radiotherapy (WBRT) or autologous hematopoietic stem cell transplantation (ASCT)?
8	Should BTK inhibitors be used to treat patients with PCNSL?
9	Should stereotactic radiosurgery be used to treat localized recurrent PCNSL patients who were refractory to chemotherapy and previously received WBRT?
10	Which is the preferred approach to make the diagnosis of a suspected PVRL, vitreous biopsy or aqueous humor/vitreous puncture?
11	Which is the preferred approach to treat PVRL patients and PCNSL patients with concurrent VRL, systemic therapy, local therapy, or combined systemic and local therapy?
