# Supplementary material for: Evidence-based expert consensus on the management of primary central nervous system lymphoma in China
Source: J Hematol Oncol. 2022 Sep 29;15:136. doi: 10.1186/s13045-022-01356-7 (PMC9524012; doi:10.1186/s13045-022-01356-7)
Supplement: Supplementary file 2 — Additional file 2. Search strategies in each database. [file 13045_2022_1356_MOESM2_ESM.pdf]

Additional file 2. Search strategies in each database

Databases searched:

- Databases in English：PubMed, EMBASE, Cochrane Library, WOS, Epistemonikos
- Databases in Chinese：CNKI, Wanfang, CBM

Time of researching：
from inception of the database through April 23, 2021

Detailed Search strategy in each database:

- **PubMed**

#1 "primary central nervous system lymphoma"[Title/Abstract]
#2 "PCNSL"[Title/Abstract]
#3 "primary CNS lymphoma"[Title/Abstract]
#4 "diffuse large b cell lymphoma"[Title/Abstract]
#5 "Lymphoma, Large B-Cell, Diffuse"[Mesh]
#6 "DLBCL"[Title/Abstract]
#7 "central nervous system"[Title/Abstract]
#8 "CNS"[Title/Abstract]
#9 #4 OR #5 OR #6
#10#7 OR #8
#11#9 AND #10
#12OR/#1-#3
#13#11 OR #12

- **EMBASE**

#1 ‘primary central nervous system lymphoma’/exp
#2 ‘diffuse large b cell lymphoma’/exp
#3 ‘central nervous system’/exp
#4 ‘primary central nervous system lymphoma’:ab,kw,ti
#5 ‘pcnsl’:ab,kw,ti
#6 ‘primary CNS lymphoma’:ab,kw,ti
#7 ‘diffuse large b cell lymphoma’:ab,kw,ti
#8 ‘dlbcl’:ab,kw,ti
#9 ‘central nervous system’:ab,kw,ti
#10‘cns’:ab,kw,ti
#11#2 OR #7 OR #8
#12#3 OR #9 OR #10
#13#11 AND #12
#14#1 OR #4 OR #5 OR#6 OR #13
#15#14 AND [medline]/lim
#16#14 NOT #15

- **Cochrane Library**

#1 MeSH descriptor: [Lymphoma, Large B-Cell, Diffuse] explode all trees
#2 MeSH descriptor: [Central Nervous System] explode all trees
#3 'diffuse large b cell lymphoma':ti,ab,kw
#4 'dlbcl':ti,ab,kw
#5 'central nervous system':ti,ab,kw
#6 'cns':ti,ab,kw
#7 #1 OR #3 OR #4
#8 #2 OR #5 OR #6
#9 #7 AND #8
#10 'primary central nervous system lymphoma':ti,ab,kw
#11 'pcnsl':ti,ab,kw
#12 'primary CNS lymphoma':ti,ab,kw
#13 #10 OR #11 OR #12
#14 #9 OR #13

- **Web of Science**

#1 Topic: (“CNS”)
#2 Topic: (“central nervous system”)
#3 Topic: (“DLBCL”)
#4 Topic: TS=(“diffuse large b cell lymphoma”)
#5 Topic: (“primary central nervous system lymphoma”)
#6 Topic: (“PCNSL”)
#7 Topic: (“primary CNS lymphoma”)
#8 #1 OR #2
#9 #3 OR #4
#10 #8 AND #9
#11 #5 OR #6 OR #7
#12 #10 OR #11

- **Epistemonikos**

#1 title/abstract:("DLBCL")
#2 title/abstract:("diffuse large b cell lymphoma")
#3 title/abstract:("CNS")
#4 title/abstract:("central nervous system")
#5 #1 OR #2
#6 #3 OR #4
#7 #5 AND #6
#8 title/abstract:("primary central nervous system lymphoma")
#9 title/abstract:("PCNSL")
#10 title/abstract:("primary CNS lymphoma")

#11 OR/#8-#10
#12 #7 OR #11

- **CNKI (database in Chinese)**

#1 “原发性中枢神经系统淋巴瘤”[主题] (Chinese characters means: primary central nervous system lymphoma)
#2 “原发中枢神经系统淋巴瘤”[主题] (Chinese characters means: primary central nervous system lymphoma)
#3 “PCNSL”[主题]
#4 “中枢神经系统弥漫大 B 细胞淋巴瘤”[主题] (Chinese characters means: central nervous system diffuse large B cell lymphoma)
#5 OR/#1-#4

- **Wanfang (database in Chinese)**

#1 “原发性中枢神经系统淋巴瘤”[主题] (Chinese characters means: primary central nervous system lymphoma)
#2 “原发中枢神经系统淋巴瘤”[主题] (Chinese characters means: primary central nervous system lymphoma)
#3 “PCNSL”[主题]
#4 “中枢神经系统弥漫大 B 细胞淋巴瘤”[主题] (Chinese characters means: central nervous system diffuse large B cell lymphoma)
#5 OR/#1-#4

- **CBM (database in Chinese)**

#1 "原发性中枢神经系统淋巴瘤"[常用字段:智能] (Chinese characters means: primary central nervous system lymphoma)
#2 "原发中枢神经系统淋巴瘤"[常用字段:智能] (Chinese characters means: primary central nervous system lymphoma)
#3 "PCNSL"[常用字段:智能]
#4 "中枢神经系统弥漫大 B 细胞淋巴瘤"[常用字段:智能] (Chinese characters means: central nervous system diffuse large B cell lymphoma)
#5 OR/#1-#4
