# Supplementary material for: Evidence-based expert consensus on the management of primary central nervous system lymphoma in China
Source: J Hematol Oncol. 2022 Sep 29;15:136. doi: 10.1186/s13045-022-01356-7 (PMC9524012; doi:10.1186/s13045-022-01356-7)
Supplement: Supplementary file 3 — Additional file 3. Supplementary data for focused clinical questions. [file 13045_2022_1356_MOESM3_ESM.pdf]

Additional file 3. Supplementary data for focused Clinical Questions

CQ 1. Which is the preferred approach to obtain the pathology specimen in patients with PCNSL, stereotactic brain biopsy or resection?

- Population: Patients with PCNSL who need to obtain the pathology specimen
- Intervention: Stereotactic brain biopsy
- Comparison: Resection

1.1. Inclusion and exclusion criteria

- inclusion criteria: we included published RCT, cohort studies and case control studies, which compared stereotactic brain biopsy with resection in patients with PCNSL who need to obtain the pathology specimen. Studies published in English and Chinese are included.
- exclusion criteria: we excluded studies where we failed to access full text, or studies without sufficient data.

1.2. Characteristic information of included studies

Supplementary Table S2.1.1. characteristic information of included studies (recent five years)

Year	Country	Journal	Study type	Age	Sample size	Group 1	Group 2	Outcomes	Conclusion
2021[1]	Tongji Hospital, China	BMC Neurology	retrospective study	Median 53.3 ± 14.3y	70 patients	Resection 28 patients	Biopsy 42 patients	Complication：Resection 10.7% vs biopsy 7.1%；OS：Resection mean 23.4m vs biopsy 11.2m；PFS：Resection mean 8.6m vs biopsy 4.6m	Compared to stereotactic biopsy, surgical resection may play a role in significantly improving OS and PFS in a subset of patients. Type of surgery and tumor location are prognostic factors for PCNSL.
2021[2]	Tiantan Hospital, China	Chinese Journal of Surgery	retrospective study	<60y count 50%	2125 patients	Resection 115 patients	Biopsy 2010 patients	Median Survival Time：Biopsy：2m（95%CI 1.76-2.24）；STR：2m（95%CI 1.4-2.6）；	Surgical resection may improve the prognosis of some patients with PCNSL. Chemothera

								GTR：19m（95 % 0-39）	py May Prolong Tumor-Specific Survival in Patients with Complete or Selected Tumor Resection.
2021[3]	Israel	J Neurosurg	retrospective database study	⩾18y	113 patients	Resection 36 patients	Biopsy 77 patients	Patients with superficial tumors who underwent resection had significantly longer survival than those who underwent biopsy (median survival 34.3 months vs. 8.9 months, P= 0.014). Patients younger than 70 years with superficial tumors who underwent	Compared to undergoing diagnostic biopsy only, a specific subgroup of patients with a single PCNSL lesion may have a survival benefit from resection.

								resection had a significantly longer survival with a median survival of 35.0 months compared to 8.9 months for the same group of patients who underwent needle biopsy (P = 0.007).	
2020[4]	Wenzhou Medical University, China	Frontiers in Oncology	retrospective database study	60-80y count 50%	3543 patients	Resection 851 patients	Biopsy or nonsurgery 2692 patients	1 year OS：Resection 59.2% vs Biopsy or nonsurgery 46.8%；3-year OS：Resection 44.7% vs Biopsy or nonsurgery 32.5% 5-year OS：Resection 36.0% vs Biopsy or	Total excision is superior to subtotal excision. Studies support the favorable impact of surgery on the clinical outcomes of patients with PCNSL. Although further randomized

								nonsurgery 26.3% Resection vs nonsurgery HR=0.81	controlled trials are needed, the currently available evidence should be considered in the clinical management of this disease.
2020[5]	The First Affiliated Hospital of Nanchang University, China	World Neurosurgery	retrospective study	2-72y	89 patients（intracranial）	Craniotomy 71 patients，GTR：57 patients STR：14 patients	Biopsy 18 patients,multiple or isolated lesions invading deep structures	Midian PFS: Resection 22±1.454m vs Biopsy 14±2.863m, P<0.05；Midian OS：Resection 33±2.998m vs Biopsy 26±2.308m, P>0.05	For intracranial PCNSL, surgical resection improves PFS but not OS. invasion of deep structures is the only independent risk factor for intracranial PCNSL.
2019[6]	US，Northwestern University,	Neurosurgery	Case-control study	IS：67y vs 63y	132 PCNSL	Craniotomy 60 patients	Biopsy 72 patients	Median Survival Time：Craniotomy	In 3 retrospective datasets, craniotomy

						46.0 m (95% CI [35.7, 133.4]) vs Biopsy 24.7 m(95% CI [13.8, 54.9]) ,HR 0.68;	was associated with improved survival compared with PCNSL biopsy.
		NCDB ：65y vs 65y	8936 patients ，NHL with CNS	Craniotomy 3423 patients	Biopsy 5513 patients	Median Survival Time ：Craniotomy 19.5m （95%CI,16.8-22.0） vs Biopsy 11.0m （95%CI,10.1-12.3 ），HR=0.83；	
		SEER：62y vs 63y vs 65y	4636 patients NHL with CNS	Craniotomy STR：216 patients GTR ：1070 patients	Biopsy 3350 patients	Median Survival Time ：Craniotomy 29m for GTR (95% CI [24, 34]), 24m for STR (95% CI [13, 40]) vs Biopsy 10m (95% CI [10, 12])	

2018[7]	Oslo University Hospital	Neurosurgical Review	retrospective database study	Median 65.3 y [range 18.9–80.7]	79 patients	Craniotomy 32 patients	Biopsy patients	47	Median OS：Craniotomy 28.6m（0.7-157.5）vs Biopsy 11.7m（0.2-136.5）Median PFS：Craniotomy 12.6m（0-157.7）vs Biopsy 7.7m（0-117）	In patients with PCNSL, resective surgery plays no role in significantly improving OS or PFS, so we do not advocate total resection as a treatment. However, cytoreductive surgery may be useful in patients with potential brain herniation.
2018[8]	Argentina	Arq Neuropsiquiatr	retrospective study	Median 59 y (range: 25–84 y)	47 patients	Resection 18 patients	Biopsy patients	29	Median Survival Time：Resection 31m（4-194）vs Biopsy 14.5（2-218）,P=0.016	Patients who had their tumors surgically removed had a median survival of 16.5 months longer than

									those who underwent biopsy alone.
2017[9]	Columbia University Irving Medical Center	Journal of Neuro-Oncology	retrospective study	Median 65 (range 21–88)	129 patients	Resection 58 patients	Biopsy 71 patients	Complication rate：Resection 17.2% vs Biopsy 28.2%，P>0.05；	Surgical resection of PCNSL is safe in selected patients, with complication rates comparable to those of other intracranial tumors. No conclusions can be drawn about the clinical benefit of resection.

NHL: non-Hodgkin's lymphoma; PCNSL: primary central nervous system lymphoma; CNS: central nervous system; OS: overall survival; PFS: progression-free survival; SEER: Surveil- lance, Epidemiology, and End Results Program; NCDB: National Cancer Database-Participant User File; GTR: gross total resection; STR: subtotal resection.

1.3. Risk of bias

Supplementary Table S2.1.2. Risk of bias of included studies using Newcastle-Ottawa Scale

Study ID	Selection of exposure				Comparability			Outcome		Total score
	Representativeness of the exposed cohort	Selection of the non-exposed cohort	Ascertainment of exposure to implants	Demonstration that outcome of interest was not present at start of study	Study controls the most important factor	Study controls for any additional factor	Assessment of outcome	Was follow up long enough for outcomes to occur	Adequacy of follow up of cohorts	
Wu et al, 2021 [1]	Yes	Yes	Yes	No	Yes	No	Yes	Yes	Yes	7
Yang et al, 2021[2]	Yes	Yes	Yes	No	No	No	No	Yes	Yes	5
Schellekes et al, 2021[3]	Yes	Yes	Yes	Yes	Yes	Yes	Yes	Yes	Yes	9
Deng et al, 2020[4]	Yes	Yes	No	No	No	Yes	No	Yes	Yes	5
Ouyang et al, 2020[5]	Yes	Yes	Yes	No	No	No	No	Yes	No	4
Rae et al, 2019[6]	Yes	Yes	Yes	No	Yes	No	Yes	Yes	Yes	7
Jahr et al, 2018[7]	Yes	Yes	Yes	Yes	Yes	Yes	Yes	Yes	Yes	9
Villalonga et al, 2018[8]	Yes	Yes	Yes	No	No	No	No	Yes	Yes	5
Cloney et al, 2017[9]	Yes	Yes	Yes	No	Yes	No	Yes	Yes	Yes	7

1.4. Meta-analysis results

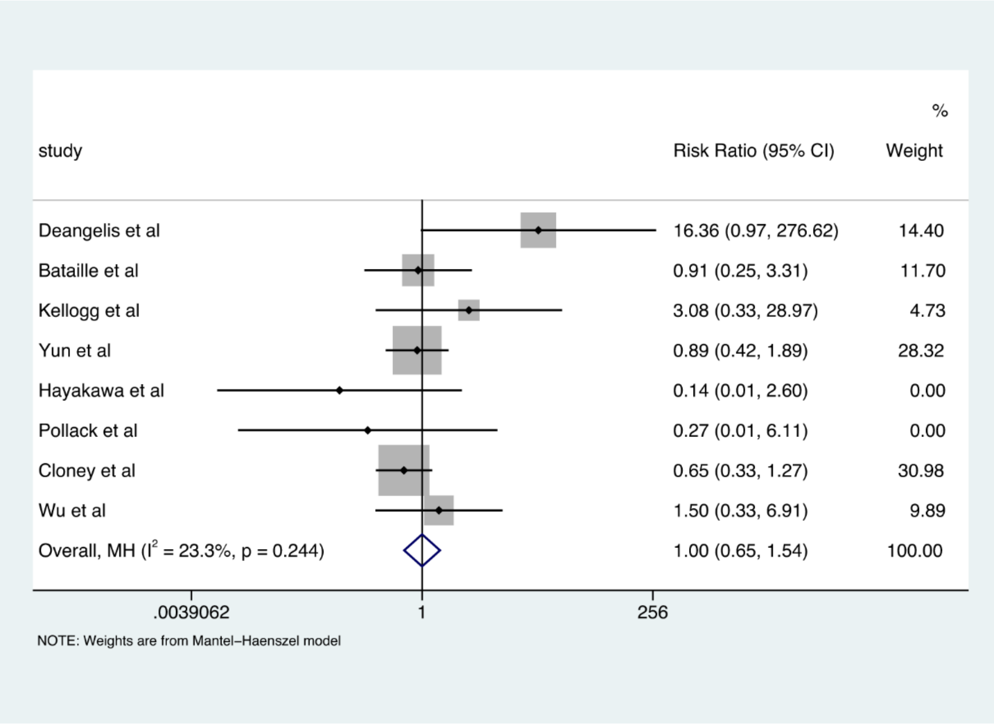

Supplementary Figure S2.1.1. Pooled results of complication incidence in patients with PCNSL who received the stereotactic brain biopsy or resection

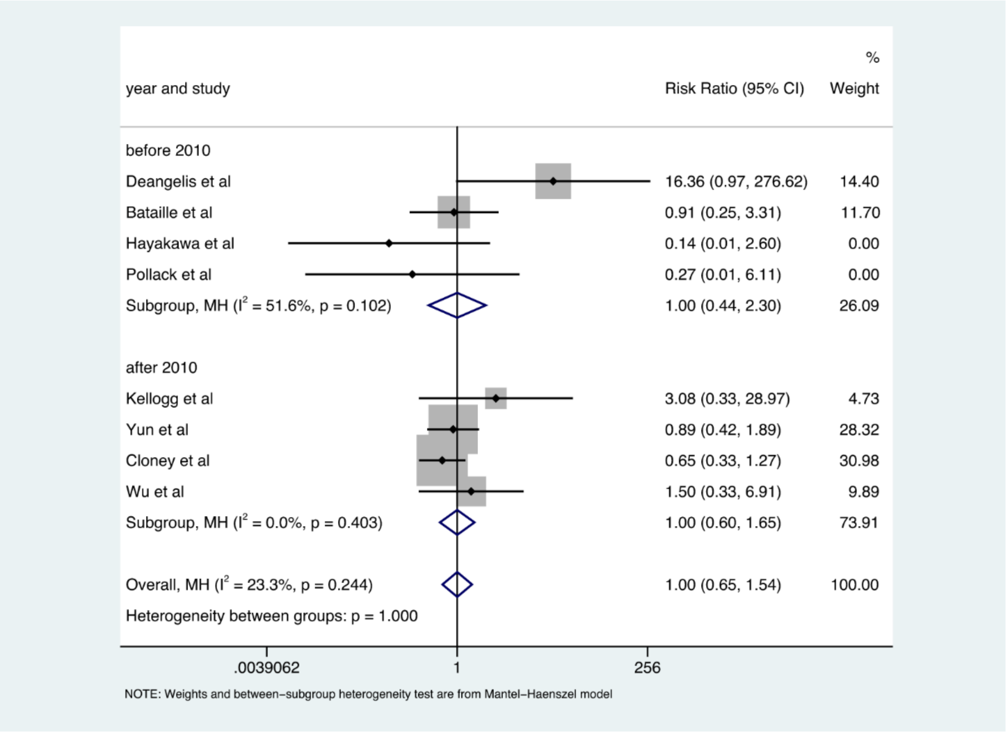

Supplementary Figure S2.1.2. Pooled results of complication incidence in patients with PCNSL who received the stereotactic brain biopsy or resection (studies published before 2010 on top, studies published after 2010 at bottom)

1.5. Summary of finding (SoF) table

Patient or population: PCNSL
Setting:
Intervention: resection
Comparison: stereotactic brain biopsy

Outcomes	Anticipated absolute effects* (95% CI)		Relative effect (95% CI)	№ of participants (studies)	Certainty of the evidence (GRADE)	Comments
	Risk with [comparison]	Risk with [intervention]				
complication incidence	121 per 1,000	121 per 1,000 (79 to 187)	RR 1.00 (0.65 to 1.54)	781 (8 observational studies)	⊕◯◯◯ VERY LOW [a]	
complication incidence before 2010	42 per 1,000	42 per 1,000 (19 to 97)	RR 1.00 (0.44 to 2.30)	406 (4 observational studies)	⊕◯◯◯ VERY LOW [b,c]	
complication incidence after 2010	189 per 1,000	189 per 1,000 (123 to 291)	RR 1.00 (0.65 to 1.54)	375 (4 observational studies)	⊕◯◯◯ VERY LOW [b]	

***The risk in the intervention group** (and its 95% confidence interval) is based on the assumed risk in the comparison group and the relative effect of the intervention (and its 95% CI).
CI: Confidence interval; RR: Risk ratio

GRADE Working Group grades of evidence
High certainty: We are very confident that the true effect lies close to that of the estimate of the effect
Moderate certainty: We are moderately confident in the effect estimate: The true effect is likely to be close to the estimate of the effect, but there is a possibility that it is substantially different
Low certainty: Our confidence in the effect estimate is limited: The true effect may be substantially different from the estimate of the effect
Very low certainty: We have very little confidence in the effect estimate: The true effect is likely to be substantially different from the estimate of effect

Explanations
a. The risk of bias was high in eight included studies; b. The risk of bias was high in four included studies; c. I^2=51.6%

1.6. References

[1] Wu S, Wang J, Liu W, et al. The role of surgical resection in primary central nervous system lymphoma: a single-center retrospective analysis of 70 patients. BMC Neurol. 2021;21(1):190. doi:10.1186/s12883-021-02227-3
[2] Yang C, Ren X, Jiang H, et al. Different treatment regimens for primary

central nervous system lymphoma: based on SEER database. Chin J Surg, 2021,59(01):52-58.

[3] Schellekes N, Barbotti A, Abramov Y, et al. Resection of primary central nervous system lymphoma: impact of patient selection on overall survival. J Neurosurg. 2021;1-10. doi:10.3171/2020.9.JNS201980

[4] Deng X, Xu X, Lin D, et al. Real-World Impact of Surgical Excision on Overall Survival in Primary Central Nervous System Lymphoma. Front Oncol. 2020;10:131. Published 2020 Feb 26. doi:10.3389/fonc.2020.00131

[5] Ouyang T, Wang L, Zhang N, et al. Clinical Characteristics, Surgical Outcomes, and Prognostic Factors of Intracranial Primary Central Nervous System Lymphoma. World Neurosurg. 2020;139:e508-e516. doi:10.1016/j.wneu.2020.04.049

[6] Rae AI, Mehta A, Cloney M, et al. Craniotomy and Survival for Primary Central Nervous System Lymphoma. Neurosurgery. 2019;84(4):935-944. doi:10.1093/neuros/nyy096

[7] Jahr G, Da Broi M, Holte H Jr, Beiske K, Meling TR. The role of surgery in intracranial PCNSL. Neurosurg Rev. 2018;41(4):1037-1044. doi:10.1007/s10143-018-0946-0

[8] Villalonga JF, Alessandro L, Farez MF, et al. The role of surgery in primary central nervous system lymphomas. Arq Neuropsiquiatr. 2018;76(3):139-144. doi:10.1590/0004-282x20180002

[9] Cloney MB, Sonabend AM, Yun J, et al. The safety of resection for primary central nervous system lymphoma: a single institution retrospective analysis. J Neurooncol. 2017;132(1):189-197. doi:10.1007/s11060-016-2358-8

CQ 2. Should corticosteroids be withdrawn from patients with suspected PCNSL/PVRL before biopsy?

- Population: suspected PCNSL/PVRL patients
- Intervention: withdrawn corticosteroids
- Comparison: not withdrawn corticosteroids

2.1. Inclusion and exclusion criteria

- inclusion criteria: we included published studies, which compared the diagnosis true positive rate or false negative rate for suspected PCNSL/PVRL patients between withdrawn corticosteroids and not withdrawn corticosteroids.
- exclusion criteria: we excluded studies where we failed to access full text, or studies without sufficient data.

2.2. Characteristic information of included studies

Supplementary Table S2.2.1. characteristic information of included studies

Study ID	Publication year	Country	N	Study type	corticosteroids dose	Duration	PCNSL/PVRL[1] Case group			Control groups		
							n	not withdrawn	withdrawn	n	not withdrawn	withdrawn
Bullis CL et al[1]	2020	America	54	Case series	Cumulative Dexamethasone 4mg-120mg	1-27d	54	18	36	-	-	-
Binnahil M et al[2]	2016	Canada	155	Case Control	mean dose of 4 mg every 6 hours	2-45d	20	15	5	135	120	15
Önder E et al[3]	2015	Turkey	25	Case series	4 mg dexamethasone with 6 hours intervals	2-30d	25	22	-	-	-	-
Manoj N et al[4]	2014	India	76	Case series	-	-	72	26	46	-	-	-
Zhao H et al[5]	2011	China	73	Case series	340mg(10-6000mg)	5.5d(1`60d)	73	39	34			
Porter AB et al[6]	2008	America	109	Case Control	25mg-6325mg	1-90d	13	8	5	94	60	34
Choi YL et al[7]	2006	South Korea	4	Case Report	-	2~18d	4	4	0		-	--
Geppert M et	1990	Germany	2	Case Repor	8 or 20 mg dexamethaso	2w	2	2	0		-	--

al[8]	t	ne<br>Daily

[1] PCNSL: primary central nervous system lymphoma；PVRL: primary vitreoretinal lymphoma

“-“: not applicable

2.3. Risk of bias

Supplementary Table S2.2.2. Risk of bias of included case control studies assessed by the Newcastle-Ottawa Scale

Study ID	Selection				Comparability		Exposure			Total score
	Is the case definition adequate?	Representativeness of the cases	Selection of Controls	Demonstration that outcome of interest was not present at start of study	Study controls the most important factor	Study controls for any additional factor	Ascertainment of exposure	Same method of ascertainment for cases and controls	Non-Response rate	
Binnahil M et al[2]	Yes	No	Yes	Yes	No	No	Yes	Yes	Yes	6
Porter AB et al[6]	Yes	No	Yes	Yes	No	No	Yes	Yes	Yes	6

Supplementary Table S2.2.3. Risk of bias of included case series assessed by the Institute of Health Economics checklist

Study ID	Study objective	Study population					Intervention and co-intervention	
	Is the hypothesis, aim, objective of the study clearly stated?	Are the characteristics of the participants included in the study described?	Were the cases collected in more than one centre?	Are the eligibility criteria for entry into the study clearly stated?	Were participants recruited consecutively?	Did participants enter the study at a similar point in the disease?	Was the intervention of interest clearly described?	Were additional interventions (co-interventions) reported in the study?

Bullis CL et al[1]	Yes	Yes	No	Yes	Unclear	No	Yes	No
Önder E et al[3]	Yes	Yes	No	Yes	Unclear	No	Yes	No
Manoj N et al[4]	Yes	Yes	No	Yes	Unclear	No	No	No
Zhao H et al[5]	Yes	Yes	No	Yes	Unclear	No	Yes	No

Continued Supplementary Table S2.2.3

Study ID	Outcome measure			Statistical analysis	Results and conclusions	
	Are the outcome measures established a priori?	Were the relevant outcomes measured with appropriate objective and/or subjective methods?	Were the relevant outcomes measured before and after the intervention?	Were the statistical tests used to assess the relevant outcomes appropriate?	Was the length of follow-up reported?	Was the loss to follow-up reported?
Bullis CL et al[1]	Yes	Yes	No	No	No	Yes
Önder E et al[3]	Yes	Yes	No	No	No	Yes
Manoj N et al[4]	Yes	Yes	No	No	No	Yes
Zhao H et al[5]	Yes	Yes	No	No	No	Yes

Continued Supplementary Table 2.2.3

Study ID	Results and conclusions			Competing interest and source of support	New items		Total score
	Does the study	Are the adverse	Are the conclusio	Are both competing interests and sources	Was the study	Were the relevant	

	provide estimates of the random variability in the data analysis of relevant outcomes?	events related with the intervention reported?	ns of the study supported by results?	of support for the study reported?	conducted prospectively?	outcomes assessed blinded to intervention status?	
Bullis CL et al[1]	No	No	Yes	Yes	No	No	9
Önder E et al[3]	No	No	Yes	Yes	No	No	9
Manoj N et al[4]	No	No	Yes	Yes	No	No	8
Zhao H et al[5]	No	No	Yes	No	No	No	8

Supplementary Table S2.2.4. JBI Critical Appraisal Checklist for Case Reports

Study ID	patient’s demographic characteristics	patient’s history	current clinical condition	diagnostic tests	intervention(s)	post-intervention clinical condition	adverse events	takeaway lessons	Total score
Choi YL et al[7]	Yes	Yes	Yes	Yes	Yes	Yes	No	Yes	7
Geppert M et al[8]	Yes	Yes	Yes	Yes	Yes	Yes	No	Yes	7

2.4 Reference

[1] Bullis CL, Maldonado-Perez A, Bowden SG, et al. Diagnostic impact of preoperative corticosteroids in primary central nervous system lymphoma. J Clin Neurosci. 2020;72:287-291.

[2] Binnahil M, Au K, Lu JQ, Wheatley BM, Sankar T. The Influence of Corticosteroids on Diagnostic Accuracy of Biopsy for Primary Central Nervous System Lymphoma. Can J Neurol Sci. 2016;43(5):721-725.

[3] Önder E, Arıkök AT, Önder S, et al. Corticosteroid pre-treated primary CNS lymphoma: a detailed analysis of stereotactic biopsy findings and consideration of interobserver variability. Int J Clin Exp Pathol. 2015;8(7):7798-7808.

[4] Manoj N, Arivazhagan A, Mahadevan A, et al. Central nervous system lymphoma: patterns of incidence in Indian population and effect of steroids on stereotactic biopsy yield. Neurol India. 2014;62(1):19-25.

[5] Zhao, H., Tian, Z., Liu, R., et al., Effect of corticosteroid administration before biopsy on histopathological diagnosis of primary central nervous system lymphoma. Chin J Lab Diagn. 2011;15(5):828-829.

[6] Porter AB, Giannini C, Kaufmann T, et al. Primary central nervous system lymphoma can be histologically diagnosed after previous corticosteroid use: a pilot study to determine whether corticosteroids prevent the diagnosis of primary central nervous system lymphoma. Ann Neurol. 2008;63(5):662-667.

[7] Choi YL, Suh YL, Kim D, Ko YH, Sung CO, Lee JI. Malignant lymphoma of the central nervous system: difficult histologic diagnosis after glucocorticoid therapy prior to biopsy. Clin Neuropathol. 2006;25(1):29-36.

[8] Geppert M, Ostertag CB, Seitz G, Kiessling M. Glucocorticoid therapy obscures the diagnosis of cerebral lymphoma. Acta Neuropathol. 1990;80(6):629-634.

CQ 3. Which is the preferred imaging examination for PCNSL patients, MRI or whole-body PET-CT?

- Population: Patients with PCNSL
- Intervention: Imaging examination by MRI
- Comparison: Imaging examination by whole-body PET-CT

3.1. Inclusion and exclusion criteria

- inclusion criteria: we included published studies, which compared the sensitivity and specificity for a PCNSL patient MRI and whole-body.
- exclusion criteria: we excluded studies where we failed to access full text, or studies without sufficient data.

3.2. Characteristic information of included studies

We didn’t identify any study met the inclusion criteria.

CQ 4. Should cognitive function assessment be used for PCNSL patients?

- Population: Patients with PCNSL
- Intervention: cognitive function assessment
- Comparison: no treatment

4.1. Inclusion and exclusion criteria

- inclusion criteria: we only included published RCTs, the benefits of cognitive function evaluation for patients were studied. Studies published in English and Chinese are included.
- exclusion criteria: we excluded studies where we failed to access full text, or studies without sufficient data.

4.2. Characteristic information of included studies

Supplementary Table 2.4.1 characteristic information of included studies

Study ID	Years	Country	Research type	Disease Stage	N	Intervention	Comparation
Meulen [1]	2018	Netherlands	Systematic review	newly diagnosed	/	cognitive functioning and HRQOL	/
Correa [2]	2007	America	Systematic review	/	/	cognitive functioning	/
Houillier [3]	2019	French	RCT	/	140	WBRT	ASCT
Meulen [4]	2021	Netherlands	RCT	/	199	standard chemotherapy+ rituximab	standard chemotherapy
Aaronson [5]	1993	England	Cross-sectional study	/	305	/	/

/: none

4.3. Risk of bias

Supplementary Table 2.4.2. Risk of bias of included systematic reviews assessed by the AMSTAR

Study ID	Was an 'a priori' design provided?	Was there duplicate study selection and data extraction?	Was a comprehensive literature search performed?	Was the status of publication (i.e. grey literature) used as an inclusion criterion?	Was a list of studies (included and excluded) provided?	Were the characteristics of the included studies provided?	Was the scientific quality of the included studies assessed and documented?	Was the scientific quality of the included studies used appropriately in formulating conclusions?	Were the methods used to combine the findings of studies appropriate?	Was the likelihood of publication bias assessed?	Was the likelihood of publication bias assessed?
Meulen [1]	No	Yes	Yes	Yes	No	Yes	Yes	Yes	Yes	No	Yes
Correa [2]	No	Yes	Yes	No	No	Yes	Yes	Yes	Yes	No	Yes
Aaronson [5]	No	Yes	Yes	Yes	No	Yes	Yes	Yes	Yes	No	Yes

Supplementary Table S2.4.3. Risk of bias of included RCTs assessed by the Cochrane Risk of Bias tool

Study ID	Random sequence generation (selection bias)	Allocation concealment (selection bias)	Blinding of participants and researchers	Blinding of outcome assessment (detection bias)	Incomplete outcome data (attrition bias)	Selective reporting (reporting bias)	Other bias

			(performance bias)				
Meulen 2021 [4]	Low ROB	Low ROB	Unclear ROB	Unclear ROB	Low ROB	Low ROB	Low ROB
Meulen [4]	Low ROB	Low ROB	Unclear ROB	Unclear ROB	Low ROB	Low ROB	Low ROB

Note: ROB: risk of bias.

Supplementary Table S2.4.4. Risk of bias of included Cross-sectional study assessed by the JBI Critical Appraisal Tools

**Study ID**	**Were the criteria for inclusion in the sample clearly defined?**	**Were the study subjects and the setting described in detail?**	**Was the exposure measured in a valid and reliable way?**	**Were objective, standard criteria used for measurement of the condition?**	**Were confounding factors identified?**	**Were strategies to deal with confounding factors stated?**	**Were the outcomes measured in a valid and reliable way?**	**Was appropriate statistical analysis used?**	**Adequacy of follow up of cohorts**
Aaronson [5]	Yes	Yes	Yes	Yes	Yes	No	Yes	Yes	Yes

4.4. Reference

[1] van der Meulen, M., Dirven, L., Habets, E.J.J., et al., Cognitive functioning and health-related quality of life in patients with newly diagnosed primary CNS lymphoma: a systematic review. The Lancet Oncology, 2018. 19(8): p. e407-e418 DOI: 10.1016/s1470-2045(18)30356-5.

[2] Correa, D.D., Maron, L., Harder, H., et al., Cognitive functions in primary central nervous system lymphoma: literature review and assessment guidelines. Ann Oncol, 2007. 18(7): p. 1145-51 DOI: 10.1093/annonc/mdl464.

[3] Houillier, C., Taillandier, L., Dureau, S., et al., Radiotherapy or Autologous Stem-Cell Transplantation for Primary CNS Lymphoma in Patients 60 Years of Age and Younger: Results of the Intergroup ANOCEF-GOELAMS Randomized Phase II PRECIS Study. J Clin Oncol, 2019. 37(10): p. 823-833 DOI: 10.1200/JCO.18.00306.

[4] van der Meulen, M., Dirven, L., Habets, E.J.J., et al., Neurocognitive functioning and radiologic changes in primary CNS lymphoma patients: results from the HOVON 105/ALLG NHL 24 randomized controlled trial. Neuro Oncol, 2021. 23(8): p. 1315-1326 DOI: 10.1093/neuonc/noab021.

[5] Aaronson, N.K., Ahmedzai, S., Bergman, B., et al., The European Organization for Research and Treatment of Cancer QLQ-C30: a quality-of-life instrument for use in international clinical trials in oncology. J Natl Cancer Inst, 1993. 85(5): p. 365-76 DOI: 10.1093/jnci/85.5.365.

CQ 5. What needs to be recommended as the combined regimen with HD-MTX backbone in induction therapy?

- Population: Patients with PCNSL
- Intervention: Combined regimen with HD-MTX
- Comparison: Other combined regimens with or without HD-MTX

5.1. Inclusion and exclusion criteria

- inclusion criteria: we only included published RCTs, which compared combined regimen with HD-MTX vs other combined regimens. Studies published in English and Chinese are included.
- exclusion criteria: we excluded studies where we failed to access full text, or studies without sufficient data.

5.2. Characteristic information of included studies

Supplementary Table S2.5.1 characteristic information of included studies

Study ID	Publication year	Country	N	intervention 1			intervention 2			Intervention 3		
				n	Intervention name	Treatment (dose, duration)	n	Intervention name	Treatment (dose, duration)	n	Intervention name	Treatment (dose, duration)
Bromberg et al[1]	2019	Netherlands, Australia, New Zealand	200	99	R-MBVP[1]	-d1+d15: MTX: 3g/m^2; d2+d3: teniposide: 100mg/m^2; d4: carmustine: 100mg/m^2; d1-5: prednisone: 60mg/m^2; c1: d0+d7+d14+d21/c2: d0+d14: rituximab: 375mg/m^2 -28d/cycle, 2 cycles	100	MBVP	-d1+d15: MTX: 3g/m^2; d2+d3: teniposide: 100mg/m^2; d4: carmustine: 100mg/m^2; d1-5: prednisone: 60mg/m^2 -28d/cycle, 2 cycles	-	-	-
Ferreri et al[2]	2009	Argentina, Greece, Italy, Peru, Portugal,	79	39	MTX[2] + cytarabine	-d1: MTX: 3.5g/m^2; d2-3: cytarabine: 2g/m^2, twice a day -3w/course, 4	40	MTX	-d1: MTX:3.5g/m^2 -3w/course, 4 courses	-	-	-

		and Switzerland				courses						
Omuro et al[3]	2015	France	95	47	MTX + procarbazine + vincristine + cytarabine	-d1+d15: MTX: 3.5g/m^2; d1-d7: procarbazine: 100 mg/m^2/d; d1+d15: vincristine: 1.4mg/m^2; d1-d5: Methylprednisolone; d8-13+d16-27: filgrastim: 5μg/kg/d. After the end of cycle 3: cytarabine: 3g/m^2: two consecutive days -28d/cycle, 3 cycles	48	MTX + temozolomide	-d1+d15: MTX: 3.5g/m^2; d1-d5: temozolomide: 150mg/m^2 Methylprednisolone: 60mg/d; Filgrastim: 5μg/kg/d -28d/cycle, 3 cycles	-	-	-
Thiel et al[4]	2010	Germany	318	154	MTX + ifosfamide + WRBT[3]	-d1: MTX:4g/m^2; d3-5: MTX+ifosfamide: 1.5g/m^2; WBRT: 45Gy in total，1.5Gy/d, 30 days	164	MTX + ifosfamide	-d1: MTX: 4g/m^2; d3-5: MTX + ifosfamide: 1.5g/m^2 -14d/cycle, 6 cycles	-	-	-

						-14d/cycle, 6 cycles						
Ferreri et al[5]	2016	Italy	21 9	75	MTX + cytarabine	-d1: MTX: 3.5g/m^2; d2-3: cytarabine: 2g/m^2, twice a day -3w/cycle, 4 cycles	69	MTX + Rituximab + cytarabine	-d1: MTX:3.5g/m^2 d2-3: cytarabine: 2g/m^2, twice a day; d-5-+0: Rituximab: 375mg/m^2 -3w/cycle, 4 cycles	7 5	MTX + cytarabine + Rituximab + thiotepa	d1: MTX：3.5g/m2; d2-3: cytarabine: 2g/m^2, twice a day; d-5-+0: Rituximab: 375mg/m^2; d4: thiotepa: 30mg/m^2
He et al[6]	2016	China	28	14	MTX + Rituximab	-Rituximab: 375mg/m^2; MTX：3g/m^2 -4w/cycle, 4 cycles	14	MTX + WBRT[3]	-MTX:3g/m^2; WBRT -4w/cycle. 4 cycles	-	-	-
Li et al[7]	2019	China	58	28	MTX + Rituximab + cytarabine	-d1: Rituximab: 375mg/m^2; d2:MTX：3.0g/m^2; d3: cytarabine: 0.5-1.0g/m^2 -21d/cycle	29	MTX + WBRT + 3-dimensional conformal radiation therapy	-WBRT:1.8~2.0Gy a time, 5 times/w, 20 times, 40~45Gy in total; 3-dimensional conformal radiation therapy 8~16Gy in	-	-	-

									total; MTX: d1:3.0g/m^2 -28d/cycle			
Luo et al[8]	2016	China	58	29	MTX + Rituximab	-Rituximab: 375mg/m^2; MTX：3g/m^2 -4w/cycle, 2-6 cycles	29	MTX + WBRT	MTX: 3g/m^2; WBRT: 1.8-2.0Gy a time, 5 times/w -4w/cycle, 2-6 cycles	-	-	-
Sun et al[9]	2017	China	52	26	MTX + Rituximab + cytarabine	-d1: Rituximab: 375mg/m^2; d2：MTX：3g/m^2; d3: cytarabine: 0.5-1.0g/m^2 -21d	26	MTX + WBRT + 3-dimensional conformal radiation therapy	WBRT: 40-45Gy in total, 1.8-2.0Gy a time, 5 times/w, 20 times; 3-dimensional conformal radiation therapy: 8-16Gy in total, 4Gy a time; MTX: 3.0g/m^2 -28d/cycle	-	-	-
Song et al[10]	2018	China	91	31	MTX + Rituximab + cytarabine+ Dexamethasone	-d1: Rituximab: 375mg/m^2; d2:HD-MTX：3.5g/m^2; d3: cytarabine: 0.5-1.0g/m^2; d2-4: dexamethasone: 10mg -4-6 courses	30	MTX + Rituximab	Rituximab: 375mg/m^2/w; MTX:3.5g/m^2/w -4w/course, 4 courses	30	MTX + WBRT	MTX: 3.5mg/m^2

Shan et al[11]	2019	China	120	60	MTX + Rituximab	-MTX：3g/m^2; Rituximab: 375mg/m^2<br>-4w/course, 4-6 courses	60	MTX + WBRT	MTX: 3g/m^2; WBRT: 1.8-2.0Gy a time, 5 times/w<br>-4w/course, 4-6 courses	-	-	-
Wang et al[12]	2016	China	60	30	MTX + Rituximab	-MTX：3g/m^2; Rituximab: 375mg/m^2<br>-4 cycles	30	MTX + WBRT	MTX:3g/m^2; WBRT<br>-1m/cycle, 4 cycles	-	-	-
Zhang et al[13]	2018	China	54	27	MTX + Rituximab + WBRT	-MTX: 3g/m^2; WBRT: 2.0Gy a time, 5 times/w, <36Gy in total; Rituximab: 375mg/m^2<br>-1m/cycle, 4 cycles	27	MTX + WBRT	-MTX:3g/m^2; WBRT:2.0Gy a time, 5 times/w, <36Gy in total<br>-1m/cycle, 4 cycles	-	-	-
Wu et al[14]	2018	China	49	24	Fotemustine + teniposide + dexamethasone	-d1: Fotemustine: 100mg/m^2，1h; d2-4: teniposide: 60mg/m^2, >0.5h; d1-5: dexamethasone: 40mg，1h<br>-21d/cycle, 4 cycles	25	HD-MA[4]	-d1: MTX:3.5g/m^2; d2-3: cytarabine: 1.0g/m^2<br>-21d/cycle, 4 cycles	-	-	-
Huang et al[15]	2017	China	48	24	MTX + temozolomide	-d1:MTX：3g/m^2; d2-6: temozolomide	24	MTX + WBRT	-d1: MTX:3.0g/m^2; WBRT: 36Gy	-	-	-

						: 150mg/m^2<br>-21d/cycle, 6-8 cycles			in total, 2Gy a time, 5 times/w<br>-21d/cycle, 6-8 cycles			
Yi et al[16]	2014	China	42	21	MTX + WBRT	-MTX: 1g/m^2;<br>WBRT: 2Gy a time, 5 times/w<br>-3w/cycle, 8 cycles	21	MTX + WBRT	-MTX：3g/m^2;<br>WBRT:2Gy a time, 5 times/w<br>-3w/cycle, 8 cycles	-	-	-

[1]R-MBVP: methotrexate, carmustine, teniposide, and prednisone (MBVP) plus rituximab

[2]MTX: methotrexate

[3]WBRT: whole brain radiation therapy

[4]HD-MA: high-dose methotrexate plus cytarabine

“-“: not applicable

5.3. Risk of bias

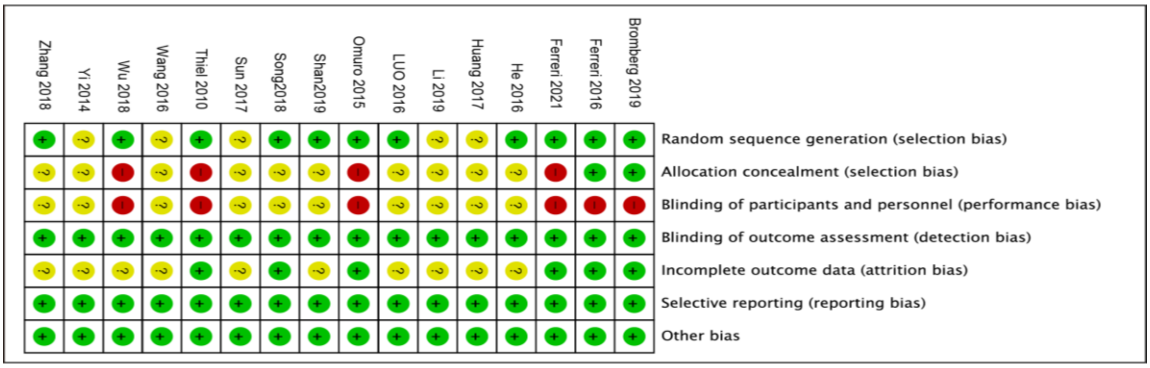

Supplementary Figure S2.5.1. Risk of bias of included studies assessed by the Cochrane Risk of Bias tool

5.4. Meta-analysis results

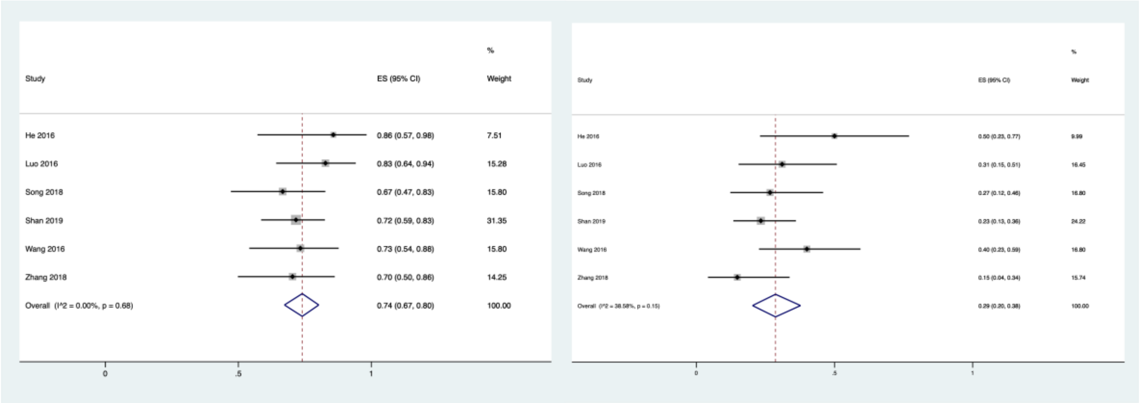

Supplementary Figure S2.5.2. Pooled results of MTX + rituximab (ORR on the left, CR on the right)

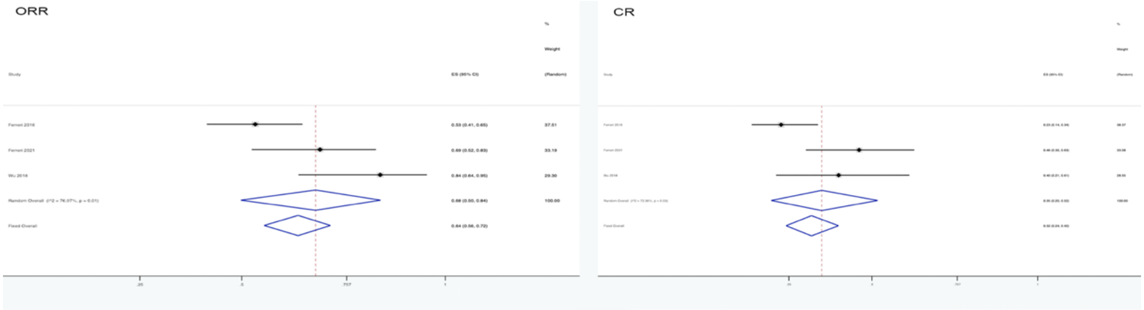

Supplementary Figure S2.5.3. Pooled results of MTX + cytarabine (ORR on the left, CR on the right)

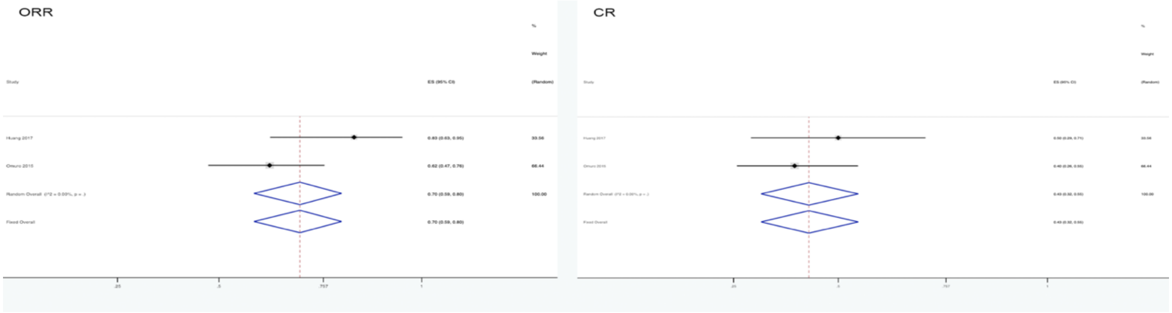

Supplementary Figure S2.5.4. Pooled results of MTX + temozolomide (ORR on the left, CR on the right)

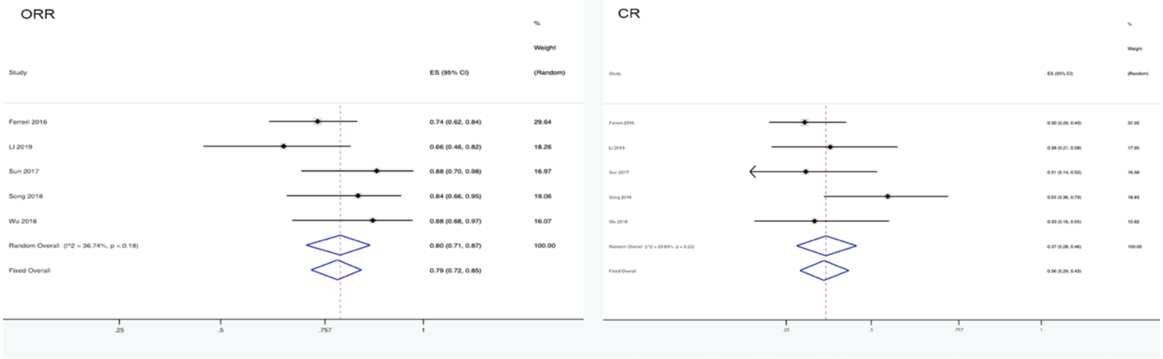

Supplementary Figure S2.5.5. Pooled results of triple therapy with HD-MTX (ORR on the left, CR on the right)

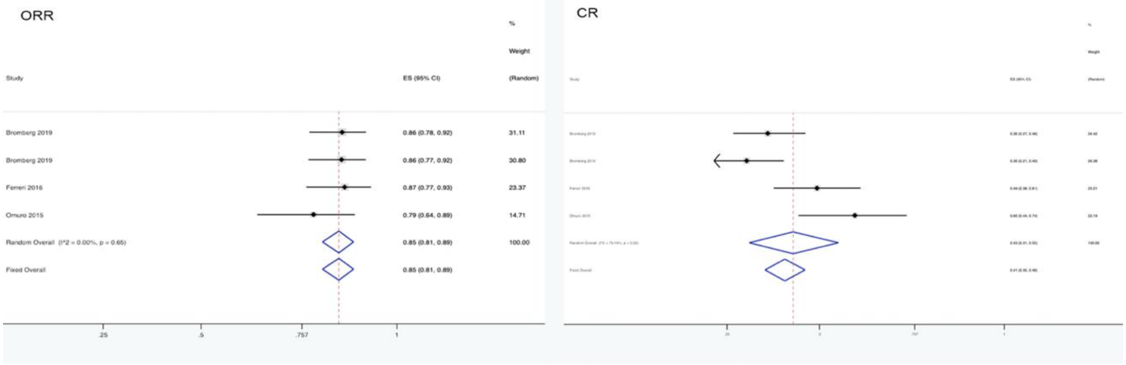

Supplementary Figure S2.5.6. Pooled results of four regimen with HD-MTX (ORR on the left, CR on the right)

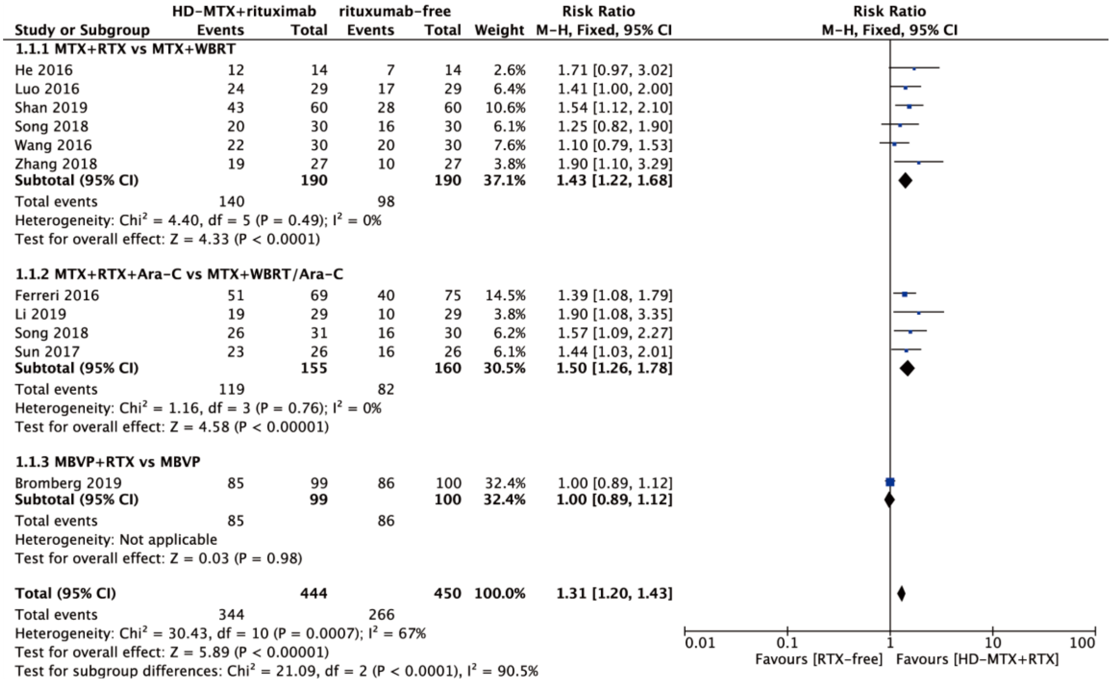

Supplementary Figure S2.5.7. Pooled ORR of HD-MTX based regimen with or without rituximab

Study or Subgroup	log[Hazard Ratio]	SE	Weight	Hazard Ratio IV, Fixed, 95% CI
Bromberg 2019	-0.113	0.0859	61.9%	0.89 [0.75, 1.06]
Ferreri 2016	-0.283	0.1095	38.1%	0.75 [0.61, 0.93]
**Total (95% CI)**			**100.0%**	**0.84 [0.73, 0.96]**

Heterogeneity: Chi² = 1.49, df = 1 (P = 0.22); I² = 33%
Test for overall effect: Z = 2.63 (P = 0.009)

Hazard Ratio
IV, Fixed, 95% CI

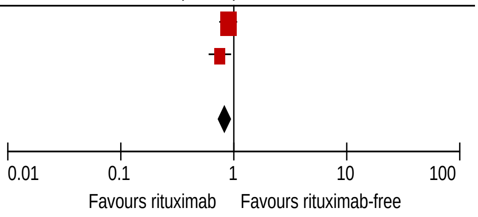

Supplementary Figure S2.5.8. Pooled PFS of HD-MTX based combined regimen with or without rituximab

Study or Subgroup	log[Hazard Ratio]	SE	Weight	Hazard Ratio IV, Fixed, 95% CI
Bromberg 2019	-0.0315	0.0988	49.7%	0.97 [0.80, 1.18]
Ferreri 2016	-0.2006	0.0983	50.3%	0.82 [0.67, 0.99]
**Total (95% CI)**			**100.0%**	**0.89 [0.78, 1.02]**

Heterogeneity: Chi² = 1.47, df = 1 (P = 0.23); I² = 32%
Test for overall effect: Z = 1.67 (P = 0.09)

Hazard Ratio
IV, Fixed, 95% CI

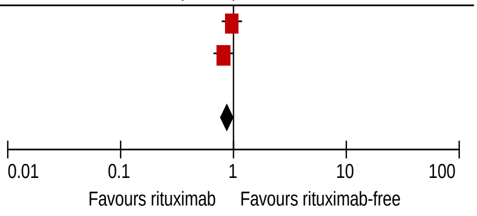

Supplementary Figure S2.5.9. Pooled OS of HD-MTX based combined regimen with or without rituximab

Study or Subgroup	HD-MTX+cytarabine Events	HD-MTX+cytarabine Total	cytarabine-free Events	cytarabine-free Total	Weight	Risk Ratio M-H, Fixed, 95% CI
Ferreri 2021	18	39	7	40	20.4%	2.64 [1.24, 5.60]
Omuro 2015	28	47	19	48	55.5%	1.51 [0.99, 2.29]
Wu 2018	10	25	8	24	24.1%	1.20 [0.57, 2.52]
**Total (95% CI)**		**111**		**112**	**100.0%**	**1.66 [1.19, 2.32]**
Total events	56		34			

Heterogeneity: Chi² = 2.40, df = 2 (P = 0.30); I² = 17%
Test for overall effect: Z = 3.00 (P = 0.003)

Risk Ratio
M-H, Fixed, 95% CI

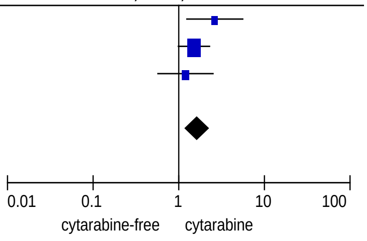

Supplementary Figure S2.5.10 Pooled ORR of HD-MTX based combined regimen with or without Cytarabine

Study or Subgroup	log[Hazard Ratio]	SE	Weight	Hazard Ratio IV, Fixed, 95% CI
Ferreri 2021	-0.18708664	0.12073848	55.6%	0.83 [0.65, 1.05]
Omuro 2015	-0.07058107	0.16077913	31.4%	0.93 [0.68, 1.28]
Wu 2018	0.02530586	0.25010317	13.0%	1.03 [0.63, 1.67]
**Total (95% CI)**			**100.0%**	**0.88 [0.74, 1.06]**

Heterogeneity: Chi² = 0.74, df = 2 (P = 0.69); I² = 0%
Test for overall effect: Z = 1.37 (P = 0.17)

Hazard Ratio
IV, Fixed, 95% CI

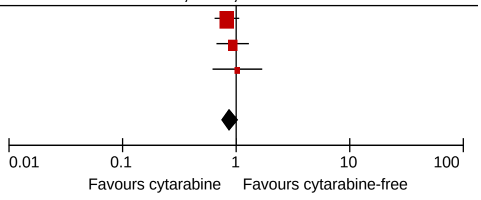

Supplementary Figure S2.5.11 Pooled OS of HD-MTX based combined regimen with or without Cytarabine

Study or Subgroup	log[Hazard Ratio]	SE	Weight	Hazard Ratio IV, Fixed, 95% CI
Ferreri 2021	-0.26760624	0.12051687	42.8%	0.77 [0.60, 0.97]
Omuro 2015	-0.09151498	0.12092607	42.5%	0.91 [0.72, 1.16]
Wu 2018	0.04921802	0.20502688	14.8%	1.05 [0.70, 1.57]
**Total (95% CI)**			**100.0%**	**0.86 [0.74, 1.01]**

Heterogeneity: Chi² = 2.13, df = 2 (P = 0.35); I² = 6%
Test for overall effect: Z = 1.85 (P = 0.06)

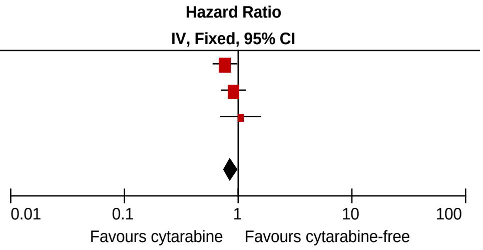

Supplementary Figure S2.5.12 Pooled PFS of HD-MTX based combined regimen with or without Cytarabine

5.5. Summary of finding (SoF) tables

Outcomes	Anticipated absolute effects* (95% CI)		Relative effect (95% CI)	№ of participants (studies)	Certainty of the evidence (GRADE)	Comments
	Risk with [comparison]	Risk with [intervention]				
MTX + rituximab vs MTX + WBRT ORR	586 per 1,000	0 per 1,000 (0 to 0)	not estimable	526 (8 studies)	⊕⊕⊕◯ Moderate [a]	
MTX + rituximab vs MTX ORR	609 per 1,000	0 per 1,000 (0 to 0)	not estimable	1094 (14 studies)	⊕⊕◯◯ Low [a,b]	
MTX + cytarabine vs MTX ORR	304 per 1,000	0 per 1,000 (0 to 0)	not estimable	223 (3 studies)	⊕⊕⊕◯ Moderate [c]	

***The risk in the intervention group** (and its 95% confidence interval) is based on the assumed risk in the comparison group and the **relative effect** of the intervention (and its 95% CI).
CI: confidence interval

GRADE Working Group grades of evidence
High certainty: we are very confident that the true effect lies close to that of the estimate of the effect.
Moderate certainty: we are moderately confident in the effect estimate: the true effect is likely to be close to the estimate of the effect, but there is a possibility that it is substantially different.
Low certainty: our confidence in the effect estimate is limited: the true effect may be substantially different from the estimate of the effect.
Very low certainty: we have very little confidence in the effect estimate: the true effect is likely to be substantially different from the estimate of effect.

Explanation
a. the risk of bias in many items is unclear; b. I^2=65; c. the simple size is under 300

5.6. Reference

[1] Bromberg JEC, Issa S, Bakunina K, et al. Rituximab in patients with primary CNS lymphoma (HOVON 105/ALLG NHL 24): a randomised, open-label, phase 3 intergroup study. Lancet Oncology. 2019;20:216-228.

[2] Ferreri AJ, Reni M, Foppoli M, et al. High-dose cytarabine plus high-dose methotrexate versus high-dose methotrexate alone in patients with primary CNS lymphoma: a randomised phase 2 trial. Lancet (london, england). 2009;374:1512 - 1520.

[3] Omuro A, Chinot O, Taillandier L, et al. Methotrexate and temozolomide versus methotrexate, procarbazine, vincristine, and cytarabine for primary CNS lymphoma in an elderly population: an intergroup ANOCEF-GOELAMS randomised phase 2 trial. The lancet Haematology. 2015;2:e251 - 9.

[4] Thiel E, Korfel A, Martus P, et al. High-dose methotrexate with or without whole brain radiotherapy for primary CNS lymphoma (G-PCNSL-SG-1): a phase 3, randomised, non-inferiority trial. Lancet Oncology. 2010;11:1036-1047.

[5] Ferreri AJ, Cwynarski K, Pulczynski E, et al. Chemoimmunotherapy with methotrexate, cytarabine, thiotepa, and rituximab (MATRix regimen) in patients with primary CNS lymphoma: results of the first randomisation of the International Extranodal Lymphoma Study Group-32 (IELSG32) phase 2 trial. Lancet Haematol. 2016;3:e217-27.

[6] He Z. Efficacy of high-dose methotrexate in combination with rituximab in the treatment of primary central nervous system lymphoma. Chinese Journal of Clinical Rational Drug Use. 2016;9:51-52.

[7] Li J. Efficacy of rituximab in combination with chemotherapy in the treatment of primary central nervous system lymphoma. The Journal of Medical Theory and Practice. 2019;32:3823-3824.

[8] Luo W, Ji L, Geng H, et al. Clinical Therapeutic Efficacy of Rituximab Combined with Methotrexate on Primary Central Nervous System Lymphoma. Journal Of Experimental Hematology. 2016;24:444-447.

[9] Sun H, Liu H, Yan J. Therapeutic effect of rituximab combined chemotherapy on primary central nervous system lymphoma. Journal of Chinese Practical Diagnosis and Therapy. 2017;31:914-916.

[10] Song C, Fang B, Cai X. Efficacy of Rituximab Combined with Chemotherapy Regimen in Treatment of Elderly Patients with Primary Central Nervous System Lymphomas. Journal of Chinese Oncology. 2018;24.

[11] Shan X. [Clinical efficacy of high-dose methotrexate in combination with rituximab in the treatment of patients with PCNSL]. Heilongjiang Medicine Journal. 2019;32:1364-1366.

[12] Wang B, Mu P. [Effect of high-dose methotrexate combined with rituximab

in the treatment of primary CNS lymphoma]. CHINA PRACTICAL MEDICINE. 2016;11:155-156.
[13] Zhang N. [Clinical effect of rituximab monoclonal antibody plus methotrexate on primary CNS lymphoma]. Laboratory Medicine and Clinic. 2018;15:768-771.
[14] Wu J, Duan L, Zhang L, et al. Fotemustine, teniposide and dexamethasone versus high-dose methotrexate plus cytarabine in newly diagnosed primary CNS lymphoma: a randomised phase 2 trial. Journal of neuro-oncology. 2018;140:427 - 434.
[15] Huang X, Wang Y. [Effects of Methotrexate Combined with Temozolomide in Treatment of Primary Central Nervous System Lymphoma]. Neural Injury and Functional Reconstruction. 2017;12:132-134.
[16] Yi Z. [Efficacy of different doses of methotrexate combined with radiotherapy in the treatment of primary central nervous system lymphoma]. Chinese Journal of Practical Nervous Diseases. 2014;17:120-121.

CQ 6. Should rituximab be used to treat newly-diagnosed PCNS-DLBCL patients in induction therapy?

- Population: Patients with PCNS-DLBCL
- Intervention: Rituximab or rituximab combined standard care
- Comparison: Placebo or standard care

6.1. Inclusion and exclusion criteria

- inclusion criteria: we only included published RCTs and observational studies in patient with PCNS-DLBCL, which compared rituximab (rituximab combined standard care) vs placebo (standard care). Studies published in English and Chinese are included.
- exclusion criteria: we excluded studies where we failed to access full text, or studies without sufficient data.

6.2. Characteristic information of included studies

Supplementary Table S2.6.1. characteristic information of included studies

Study ID	Country	Study design	N	Intervention group				Control group			
				n	Intervention	Medication regimen	Course	n	Control	Medication regimen	Course
Bromberg JEC et al, 2019 [1]	Netherlands, Australia, New Zealand	RCT	199	99	R-MBVP	-Intravenous rituximab 375 mg per m^2 on days 0, 7, 14, and 21 in cycle one and days 0 and 14 in cycle two.<br>-Methotrexate 3 g per m^2 on days 1 and 15 of 28-day cycles, intravenous teniposide 100 mg per m^2 on days 2 and 3, intravenous carmustine 100 mg per m^2 on day 4, and oral prednisolone 60 mg per m^2 on days 1-5.	28d/cycle, 2 cycles	100	MBVP	Methotrexate 3 g per m2 on days 1 and 15 of 28-day cycles, intravenous teniposide 100 mg per m^2 on days 2 and 3, intravenous carmustine 100 mg per m^2 on day 4, and oral prednisolone 60 mg per m^2 on days 1-5.	28d/cycle, 2 cycles

Ferreri AJM et al, 2016 [2]	Denmark, Germany, Italy, Switzerland, United Kingdom	RCT	144	69	R-MA	-Two doses of rituximab 375 mg/m^2 on days -5 and 0. -Methotrexate 3.5g/m^2 (0·5 g/m^2 in 15 min, followed by 3 g/m^2 in a 3-h infusion) on day 1 and cytarabine 2 g/m^2 (1-h infusion, twice daily, every 12 h) on days 2 and 3.	3w/cycle, 4 cycles	75	MA	Methotrexate 3.5g/m^2 (0·5 g/m^2 in 15 min, followed by 3 g/m^2 in a 3-h infusion) on day 1 and cytarabine 2 g/m^2 (1-h infusion, twice daily, every 12 h) on days 2 and 3.	3w/cycle, 4 cycles
Patekar M et al, 2019 [3]	India	Retrospective cohort study	73	27	R-MVP	-Rituximab 375 mg/m^2 on day1. -Methotrexate 3.5g/m^2 IV day 1 with hydration, alkalinisation and leucovorin rescue (25 mg IV every 6 h day 2–4),	2w/cycle, 5 cycles	46	MVP	Methotrexate 3.5g/m^2 IV day 1 with hydration, alkalinisation and leucovorin rescue (25 mg IV every 6 h day 2–4), vincristine 1.4 mg/m^2 (capped at 2 mg) IV day 1,	2w/cycle, 5 cycles

						vincristine 1.4 mg/m^2 (capped at 2 mg) IV day 1, procarbazine 100 mg/m^2 P.O. days1-7 in odd number cycles.				procarbazine 100 mg/m^2 P.O. days1-7 in odd number cycles.	
Chen C et al, 2019 [4]	China	Retrospective cohort study	62	32	R-MT	-Rituximab 375 mg/m^2 on day 0. - Methotrexate (3.5g/m^2) was intravenously administered on day 1, and temozolomide (150mg/m^2) was orally administered on days 1 - 5.	3w/cycle, 6-8 cycles	30	MT	Methotrexate (3.5g/m^2) was intravenously administered on day 1, and temozolomide (150mg/m^2) was orally administered on days 1 - 5.	3w/cycle, 6-8 cycles
Da Broi M et al, 2018 [5]	Norway	Retrospective cohort study	43	18	R-MVP	- Rituximab 375 mg/m^2 on day1. - Methotrexate 3.5g/m^2 IV day 1 with	2w/cycle, 5 cycles	25	MVP	- Methotrexate 3.5g/m^2 IV day 1 with hydration, alkalinisation and	2w/cycle, 5 cycles

						hydration, alkalinisation and leucovorin rescue (25 mg IV every 6 h day 2–4), vincristine 1.4 mg/m^2 (capped at 2 mg) IV day 1, procarbazine 100 mg/m^2 P.O. days1-7 in odd number cycles.				leucovorin rescue (25 mg IV every 6 h day 2–4), vincristine 1.4 mg/m^2 (capped at 2 mg) IV day 1, procarbazine 100 mg/m^2 P.O. days1-7 in odd number cycles.	
Sun X et al, 2017 [6]	China	Retrospective cohort study	60	36	R-MAD	-Rituximab 375 mg/m^2 on day 0.<br>-High dose methotrexate was administered intravenously at a dose of 3.5 g/m^2 over 3 hours on day 1; Ara-C was administered intravenously at (0.5–1) g/m^2 on day	3w/cycle, 6 cycles	24	MAD	High dose methotrexate was administered intravenously at a dose of 3.5 g/m^2 over 3 hours on day 1; Ara-C was administered intravenously at (0.5–1) g/m^2 on day 2; dexamethasone was	3w/cycle, 6 cycles

Houillier C et al, 2017 [7]	France	Retrospective cohort study	90	39	R-MPV-AAA	2; dexamethasone was administered at 5-10 mg on days 1-3. -Rituximab 375 mg/m^2 on day 0. -Methotrexate was administered intravenously at a dose of 3.5 g/m^2 on day 1 and day 15; procarbazine was administered at a dose of 100 mg/m^2/day from day 1 to day 7; vincristine was administered at a dose of 1.4 g/m^2 on day 1 and day 15; cytarabine	-MPV: 4w/cycle, 3 cycles; -AAA: 3 cycles	51	MPV-AAA	administered at 5-10 mg on days 1-3. Methotrexate was administered intravenously at a dose of 3.5 g/m^2 on day 1 and day 15; procarbazine was administered at a dose of 100 mg/m^2/day from day 1 to day 7; vincristine was administered at a dose of 1.4 g/m^2 on day 1 and day 15; cytarabine consolidation was administered at a dose of 3	-MPV: 4w/cycle, 3 cycles; -AAA: 3 cycles

						consolidation was administered at a dose of 3 g/m^2 on day 1 and day 2.				g/m^2 on day 1 and day 2.	
Mocikova H et al, 2016 [8]	Czech	Retrospective cohort study	164	49	R-MVP	-Rituximab 375 mg/m^2 on day 1. -Methotrexate, 1-3.5g/m^2 intravenously day 1 in 4 h infusion, vincristine, 2 mg intravenously day 1, and procarbazine 100mg/m2 orally day 1-7 in odd courses.	2w/cycle, 5-7 cycles	115	MVP	Methotrexate, 1-3.5g/m^2 intravenously day 1 in 4 h infusion, vincristine, 2 mg intravenously day 1, and procarbazine 100mg/m2 orally day 1-7 in odd courses.	2w/cycle, 5-7 cycles
Madle M et al, 2015 [9]	Germany	Retrospective cohort study	79	27	R+ Combination chemotherapy	-Rituximab -Multiple combination chemotherapy (with or without high-dose methotrexate)	NA	52	Combination chemotherapy	Multiple combination chemotherapy (with or without high-dose methotrexate)	NA
Kansar	Canada	Retrosp	74	25	R+HDMTX	-Rituximab	2w/cycl	49	HDMTX	High-dose	2w/cycle,

a R et al, 2015 [10]		ective cohort study				375 mg/m^2 on day 1 or day 2. -High-dose methotrexate 8g/m^2	e, 4 cycles			methotrexate 8g/m^2	4 cycles
Holdhoff M et al, 2014 [11]	America	Retrospective cohort study	81	27	R+HDMTX	-Rituximab 375 mg/m^2 on day 3 -High-dose methotrexate 8g/m^2	2w/cycle, 5 cycles	54	HDMTX	High-dose methotrexate 8g/m^2	2w/cycle, 5 cycles
Gregory G et al, 2013 [12]	Australia	Retrospective cohort study	117	18	R+ Combination chemotherapy	-Rituximab 375 mg/m^2 -Methotrexate dose of 8 g/m^2 per cycle vs 2–3.5 g/m^2, cytarabine, rituximab, radiotherapy, and intrathecal methotrexate	2-7 cycles	99	Combination chemotherapy	Methotrexate dose of 8 g/m^2 per cycle vs 2-3.5g/m^2, cytarabine, rituximab, radiotherapy, and intrathecal methotrexate	NA
Birnbaum T et al, 2012 [13]	Germany	Retrospective cohort study	36	17	R-MI	-Rituximab 375 mg/m^2 on day 0. -Methotrexate 4 g/m^2 on day 1 and ifosfamide	2w/cycle, 6 cycles	19	MI	Methotrexate 4 g/m^2 on day 1 and ifosfamide 1.5 g/m^2 on days 3-5; dexamethasone was	2w/cycle, 6 cycles

	1.5 g/m^2 on days 3-5; dexamethasone was given for 10 days during the first cycle only.		given for 10 days during the first cycle only.	

Note: R-MBVP: methotrexate, carmustine, teniposide, and prednisone (MBVP) plus rituximab; HDMTX: High dose methotrexate; R: Rituximab; HD-MA: high-dose methotrexate plus cytarabine; MVP: Methotrexate, vincristine, and procarbazine; MA: Methotrexate and Ara-C; MT: Methotrexate and temozolomide; MAD: Methotrexate, Ara-C and dexamethasone; MI: Methotrexate and ifosfamide; “NA “: not applicable.

6.3. Risk of bias

Supplementary Table S2.6.2. Risk of bias of included RCTs assessed by the Cochrane Risk of Bias tool

Study ID	Random sequence generation (selection bias)	Allocation concealment (selection bias)	Blinding of participants and researchers (performance bias)	Blinding of outcome assessment (detection bias)	Incomplete outcome data (attrition bias)	Selective reporting (reporting bias)	Other bias
Bromberg JEC et al, 2019 [1]	Low ROB	Low ROB	High ROB	Unclear ROB	Low ROB	Low ROB	Low ROB
Ferreri AJM et al, 2016 [2]	Low ROB	Low ROB	High ROB	Unclear ROB	Low ROB	Low ROB	Low ROB

Supplementary Table S2.6.3. Risk of bias of included cohort studies assessed by the Newcastle-Ottawa Scale

Study ID	Selection of exposure				Comparability			Outcome		Total score
	Representativeness of the exposed cohort	Selection of the non-exposed cohort	Ascertainment of exposure to implants	Demonstration that outcome of interest was not present at start of study	Study controls the most important factor	Study controls for any additional factor	Assessment of outcome	Was follow up long enough for outcomes to occur	Adequacy of follow up of cohorts	
Patekar M et al, 2019 [3]	Yes	Yes	Yes	Yes	Yes	No	No	Yes	Yes	7
Chen C et al, 2019 [4]	Yes	Yes	Yes	Yes	Yes	Yes	No	Yes	Yes	8

Da Broi M et al, 2018 [5]	Yes	Yes	Yes	Yes	Yes	Yes	No	Yes	Yes	8
Sun X et al, 2017 [6]	Yes	Yes	Yes	Yes	Yes	No	No	Yes	Yes	7
Houillier C et al, 2017 [7]	Yes	Yes	Yes	Yes	No	No	No	Yes	Yes	6
Mocikova H et al, 2016 [8]	Yes	Yes	Yes	Yes	Yes	Yes	No	Yes	Yes	8
Madle M et al, 2015 [9]	Yes	Yes	Yes	Yes	Yes	No	No	Yes	Yes	7
Kansara R et al, 2015 [10]	Yes	Yes	Yes	Yes	Yes	Yes	No	Yes	Yes	8
Holdhoff M et al, 2014 [11]	Yes	Yes	Yes	Yes	Yes	Yes	No	Yes	Yes	8
Gregory G et al, 2013 [12]	Yes	Yes	Yes	Yes	Yes	Yes	No	Yes	Yes	8
Birnbaum T et al, 2012 [13]	Yes	Yes	Yes	Yes	Yes	No	Yes	Yes	Yes	8

Note: "*" equals "low risk of bias"; "-" equals "high risk of bias".

6.4. Meta-analysis results

Study or Subgroup	rituximab Events	rituximab Total	non-rituximab Events	non-rituximab Total	Weight	Risk Ratio M-H, Fixed, 95% CI
Birnbaum 2012	17	17	17	19	5.8%	1.11 [0.93, 1.33]
Bromberg 2019	80	99	75	100	26.2%	1.08 [0.93, 1.25]
Chen 2019	30	32	20	29	7.4%	1.36 [1.05, 1.76]
Da Broi 2018	17	18	14	25	4.1%	1.69 [1.17, 2.43]
Ferreri 2016	51	69	40	75	13.5%	1.39 [1.08, 1.79]
Houillier 2017	30	39	27	51	8.2%	1.45 [1.07, 1.98]
Kansara 2015	12	25	25	49	5.9%	0.94 [0.58, 1.54]
Mocikova 2016	32	49	61	115	12.8%	1.23 [0.94, 1.61]
Patekar 2019	24	27	34	46	8.8%	1.20 [0.97, 1.49]
Sun 2017	26	36	17	24	7.2%	1.02 [0.74, 1.41]
**Total (95% CI)**		**411**		**533**	**100.0%**	**1.22 [1.12, 1.32]**
Total events	319		330			

Heterogeneity: Chi² = 11.74, df = 9 (P = 0.23); I² = 23%
Test for overall effect: Z = 4.60 (P < 0.00001)

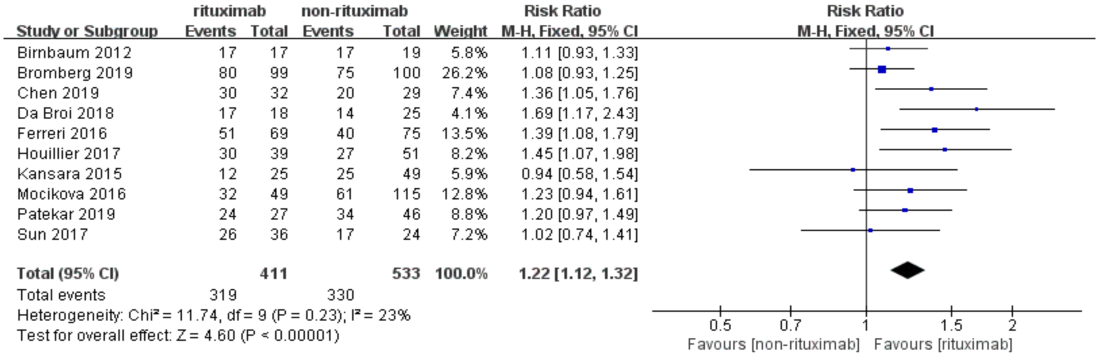

Supplementary Figure S2.6.1. Meta-analysis for OR in patient with PCNSL (rituximab vs. non-rituximab)

Study or Subgroup	rituximab Events	rituximab Total	non-rituximab Events	non-rituximab Total	Weight	Risk Ratio M-H, Fixed, 95% CI
Birnbaum 2012	17	17	13	19	6.7%	1.44 [1.05, 1.97]
Bromberg 2019	67	99	66	100	34.4%	1.03 [0.84, 1.25]
Chen 2019	17	32	8	29	4.4%	1.93 [0.98, 3.78]
Da Broi 2018	17	18	14	25	6.1%	1.69 [1.17, 2.43]
Ferreri 2016	21	69	17	75	8.5%	1.34 [0.78, 2.33]
Holdhoff 2014	19	27	19	54	6.6%	2.00 [1.29, 3.10]
Kansara 2015	9	25	17	49	6.0%	1.04 [0.54, 1.98]
Mocikova 2016	20	49	39	115	12.2%	1.20 [0.79, 1.84]
Patekar 2019	22	27	26	46	10.1%	1.44 [1.06, 1.97]
Sun 2017	24	36	8	24	5.0%	2.00 [1.09, 3.69]
**Total (95% CI)**		**399**		**536**	**100.0%**	**1.34 [1.18, 1.51]**
Total events	233		227			

Heterogeneity: Chi² = 15.94, df = 9 (P = 0.07); I² = 44%
Test for overall effect: Z = 4.61 (P < 0.00001)

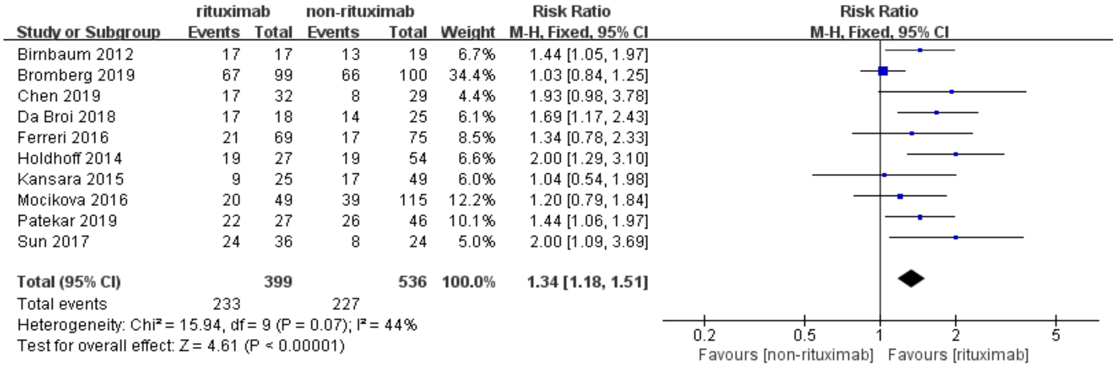

Supplementary Figure S2.6.2. Meta-analysis for CR in patient with PCNSL (rituximab vs. non- rituximab)

Study or Subgroup	rituximab Events	rituximab Total	non-rituximab Events	non-rituximab Total	O-E	Variance	Weight	Hazard Ratio Exp[(O-E) / V], Fixed, 95% CI
Birnbaum 2012	0	0	0	0	0.77	5.91	5.3%	1.14 [0.51, 2.55]
Bromberg 2019	0	0	0	0	-6.88	26.32	23.5%	0.77 [0.53, 1.13]
Chen 2019	0	0	0	0	-1.74	2.85	2.5%	0.54 [0.17, 1.73]
Da Broi 2018	0	0	0	0	-0.6	2.67	2.4%	0.80 [0.24, 2.65]
Ferreri 2016	0	0	0	0	-5.82	8.91	8.0%	0.52 [0.27, 1.00]
Holdhoff 2014	0	0	0	0	-5.77	7.3	6.5%	0.45 [0.22, 0.94]
Houillier 2017	0	0	0	0	-0.22	16.14	14.4%	0.99 [0.61, 1.61]
Kansara 2015	0	0	0	0	-3.3	11.47	10.2%	0.75 [0.42, 1.34]
Mocikova 2016	0	0	0	0	-6.4	26.09	23.3%	0.78 [0.53, 1.15]
Sun 2017	0	0	0	0	-4.55	4.29	3.8%	0.35 [0.13, 0.89]
**Total (95% CI)**		**0**		**0**			**100.0%**	**0.73 [0.61, 0.88]**
Total events	0		0					

Heterogeneity: Chi² = 8.17, df = 9 (P = 0.52); I² = 0%
Test for overall effect: Z = 3.26 (P = 0.001)

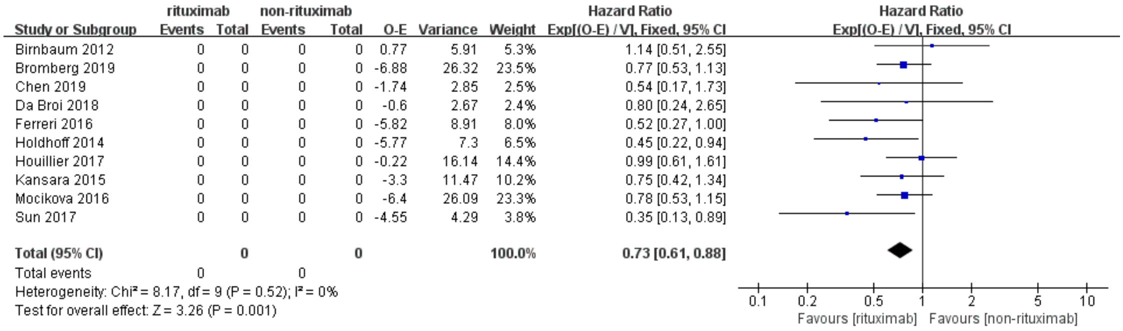

Supplementary Figure S2.6.3. Meta-analysis for PFS in patient with PCNSL (rituximab vs. non- rituximab)

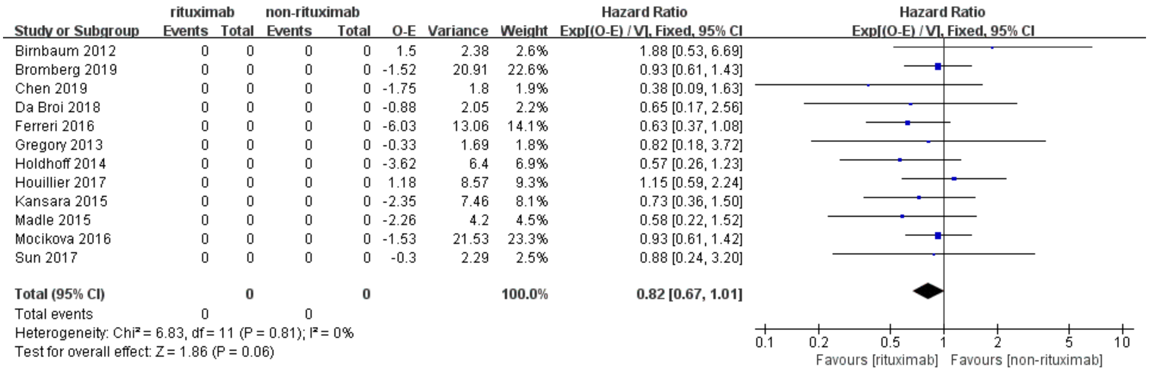

Supplementary Figure S2.6.4. Meta-analysis for OS in patient with PCNSL (rituximab vs. non- rituximab)

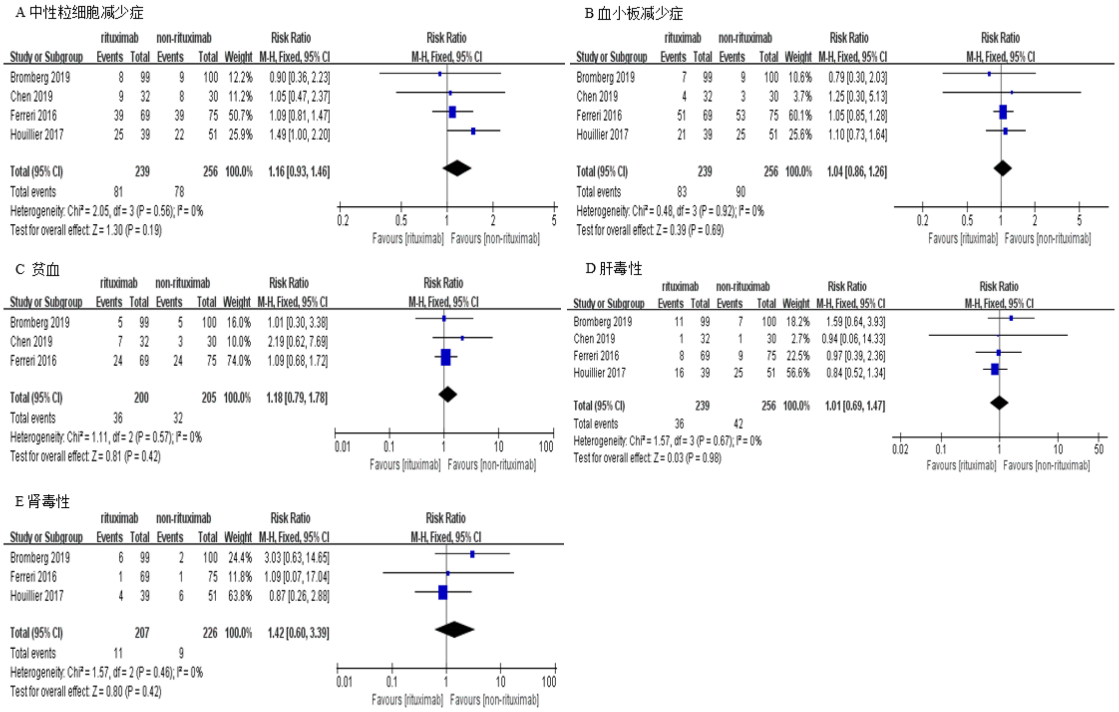

Supplementary Figure S2.6.5. Grade 3 or higher adverse events: A Neutropenia; B Thrombocytopenia; C Anemia; D Hepatotoxicity; E Nephrotoxicity.

6.5. Summary of finding (SoF) tables

Outcomes	Anticipated absolute effects* (95% CI)		Relative effect (95% CI)	№ of participants (studies)	Certainty of the evidence (GRADE)	Comments
	Risk with [comparison]	Risk with [intervention]				
**OR**	619 per 1,000	755 per 1,000 (693 to 817)	**RR 1.22** (1.12 to 1.32)	944 (10 studies)	⊕⊕◯◯ Low[a]	None
**CR**	424 per 1,000	568 per 1,000 (500 to 639)	**RR 1.34** (1.18 to 1.51)	935 (10 studies)	⊕⊕◯◯ Low[a]	None
**PFS**	NA	NA	**HR 0.73** (0.61 to 0.88)	953 (10 studies)	⊕⊕◯◯ Low[a]	None
**OS**	NA	NA	**HR 0.82** (0.67 to 1.01)	1149 (12 studies)	⊕⊕◯◯ Low[a]	None

*The risk in the intervention group (and its 95% confidence interval) is based on the assumed risk in the comparison group and the relative effect of the intervention (and its 95% CI). CI: Confidence interval; RR: Risk ratio; HR: Hazard ratio; NA: Not applicable.

Explanation

[a] The initial quality of evidence for the results of meta-analyses of observational studies was low.

6.6. References

[1] Bromberg JEC, Issa S, Bakunina K, et al. Rituximab in patients with primary CNS lymphoma (HOVON 105/ALLG NHL 24): a randomised, open-label, phase 3 intergroup study. The Lancet Oncology. 2019;20(2):216‐28.

[2] Ferreri AJM, Cwynarski K, Pulczynski E, et al. Chemoimmunotherapy with methotrexate, cytarabine, thiotepa, and rituximab (MATRix regimen) in patients with primary CNS lymphoma: results of the first randomisation of the International Extranodal Lymphoma Study Group-32 (IELSG32) phase 2 trial. The lancet haematology. 2016;3(5):e217‐e27.

[3] Patekar M, Adhikari N, Biswas A, et al. Primary CNS Lymphoma in India: A 17-Year Experience From the All India Institute of Medical Sciences. Journal of global oncology. 2019;5:1-9.

[4] Chen C, Sun P, Cui J, et al. High-dose Methotrexate plus temozolomide with or without rituximab in patients with untreated primary central nervous system lymphoma: A retrospective study from China. Cancer medicine. 2019;8(4):1359-67.

[5] Da Broi M, Jahr G, Beiske K, et al. Efficacy of the Nordic and the MSKCC chemotherapy protocols on the overall and progression-free survival in intracranial PCNSL. Blood cells, molecules & diseases. 2018;73:25-32.

[6] Sun X, Liu J, Wang Y, et al. Methotrexate-cytarabine-dexamethasone combination chemotherapy with or without rituximab in patients with primary central nervous system lymphoma. Oncotarget. 2017;8(30):49156-64.

[7] Houillier C, Ghesquières H, Chabrot C, et al. Rituximab, methotrexate, procarbazine, vincristine and intensified cytarabine consolidation for primary central nervous system lymphoma (PCNSL) in the elderly: a LOC network study. Journal of neuro-oncology. 2017;133(2):315-20.

[8] Mocikova H, Pytlik R, Sykorova A, et al. Role of rituximab in treatment of patients with primary central nervous system lymphoma: a retrospective analysis of the Czech lymphoma study group registry. Leukemia & lymphoma. 2016;57(12):2777-83.

[9] Madle M, Krämer I, Lehners N, et al. The influence of rituximab, high-dose therapy followed by autologous stem cell transplantation, and age in patients with primary CNS lymphoma. Annals of hematology. 2015;94(11):1853-7.

[10] Kansara R, Shenkier TN, Connors JM, et al. Rituximab with high-dose methotrexate in primary central nervous system lymphoma. American journal of hematology. 2015;90(12):1149-54.

[11] Holdhoff M, Ambady P, Abdelaziz A, et al. High-dose methotrexate with or without rituximab in newly diagnosed primary CNS lymphoma. Neurology. 2014;83(3):235-9.

[12] Gregory G, Arumugaswamy A, Leung T, et al. Rituximab is associated with improved survival for aggressive B cell CNS lymphoma. Neuro-oncology. 2013;15(8):1068-73.
[13] Birnbaum T, Stadler EA, von Baumgarten L, et al. Rituximab significantly improves complete response rate in patients with primary CNS lymphoma. Journal of neuro-oncology. 2012;109(2):285-91.

CQ 7. Which is the preferred approach to treat patients with PCNSL at consolidation therapy, whole-brain radiotherapy (WBRT) or autologous hematopoietic stem cell transplantation (ASCT)?

- Population: Patients with PCNSL
- Intervention: Autologous hematopoietic stem cell transplantation (ASCT)
- Comparison: Whole-brain radiotherapy (WBRT)

7.1. Inclusion and exclusion criteria

- inclusion criteria: we only included published RCTs and observational studies in patient with PCNS, which compared whole-brain radiotherapy vs autologous hematopoietic stem cell transplantation. Studies published in English and Chinese are included.
- exclusion criteria: we excluded studies where we failed to access full text, or studies without sufficient data.

7.2. Characteristic information of included studies

Supplementary Table S2.7.1. Characteristic information of included studies

Study ID	Country	Study design	N	Intervention group			Control group		
				n	Intervention	Medication regimen	n	Control	Medication regimen
Ferreri AJM et al, 2017 [1]	Italy	RCT	118	58	HDC-ASCT	D-6: carmustine (400 mg/m²); D-5,-4: thiotepa (5 mg/kg q12h) D0: re-infusion of autologous peripheral blood stem cells	55	WBRT	36 Gy, with the addition of a 9 Gy tumour-bed boost in patients in partial response: photons of 4-10 MeV; 180 cGy/fraction; 5 d/wk
Houillier C et al, 2019 [2]	France	RCT	140	44	HDC-ASCT	D-9,-8,-7: thiotepa (250 mg/m2/d); D-6,-5,-4: busulfan (8 mg/kg); D-3,-2: Cyclophosphamide (60 mg/kg/day); D-3: polyethylene glycol filgrastim; D0: re-infusion of autologous peripheral blood stem cells	53	WBRT	40 Gy: photons of 6-10 MeV; 2 Gy/fraction; 5 d/wk
Correa DD et al, 2019 [3]	America	Observational study	29	15	HDC-ASCT	Thiotepa, busulfan, cyclophosphamide, and autohematopoietic stem cell transplantation	14	rdWBRT + Ara-C	23.4 Gy: 1.8 Gy/fraction; 13 day; two cycles of Ara-C

Ferreri AJM et al, 2020 [4]	Italy	Observational study	28	5	HDC-ASCT	Carmustine, thiotepa, and autohematopoietic stem cell transplantation	7	WBRT	30-36 Gy
Houillier C et al, 2020 [5]	France	Observational study	1002	56	HDC-ASCT	Thiotepa-based HDC-ASCT: 86%; BEAM-ASCT (carmustine, etoposide, cytarabine, melphalan + autologous hematopoietic stem cell transplantation): 14%	149	WBRT	18-56 Gy: >30 Gy: 58%; ≤30 Gy: 32%; NA: 10%
Kim JE et al, 2012 [6]	Korea	Observational study	65	18	Chemotherapy-ASCT	NA	13	Chemotherapy-WBRT	NA

HDC: high-dose chemotherapy; WBRT: whole-brain radiotherapy; ASCT: autologous hematopoietic stem cell transplantation; NA: not applicable.

7.3. Risk of bias

Table S2.7.2 Risk of bias of included RCTs assessed by the Cochrane Risk of Bias tool

Study ID	Random sequence generation (selection bias)	Allocation concealment (selection bias)	Blinding of participants and researchers (performance bias)	Blinding of outcome assessment (detection bias)	Incomplete outcome data (attrition bias)	Selective reporting (reporting bias)	Other bias
Ferreri AJM et al, 2017 [1]	Low ROB	Low ROB	High ROB	Unclear ROB	Low ROB	Low ROB	Low ROB
Houillier C et al, 2019 [2]	Low ROB	Low	High	Unclear ROB	Low ROB	Low ROB	Low ROB

Note: ROB: risk of bias.

Supplementary Table S2.7.3. Risk of bias of included observational studies assessed by the Newcastle-Ottawa Scale

Study ID	Selection of exposure				Comparability			Outcome		Total score
	Representativeness of the exposed cohort	Selection of the non-exposed cohort	Ascertainment of exposure to implants	Demonstration that outcome of interest was not present at start of study	Study controls the most important factor	Study controls for any additional factor	Assessment of outcome	Was follow up long enough for outcomes to occur	Adequacy of follow up of cohorts	
Rorrea DD et al, 2019 [3]	Yes	Yes	Yes	Yes	No	No	No	Yes	Yes	6
Ferreri AJM et al, 2020 [4]	Yes	Yes	Yes	Yes	No	No	No	Yes	Yes	6

Houillier C et al, 2020 [5]	Yes	Yes	Yes	Yes	Yes	No	No	Yes	Yes	7
Kim JE et al, 2012 [6]	Yes	Yes	Yes	Yes	Yes	No	No	Yes	Yes	7

Note: “*” equals “low risk of bias”; “-” equals “high risk of bias”.

7.4. Meta-analysis results

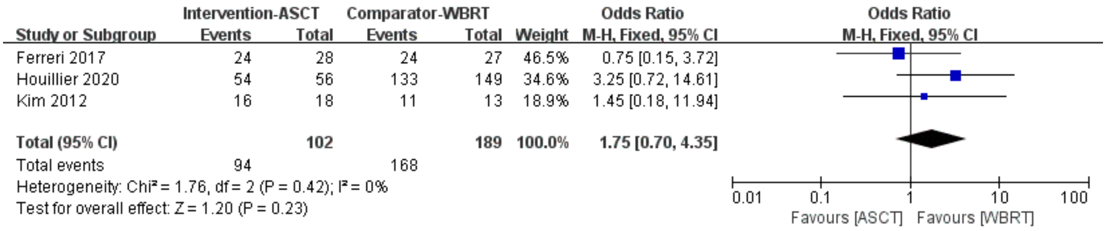

Supplementary Figure S2.7.1. Meta-analysis for OR in patient with PCNSL (ASCT vs. WBRT)

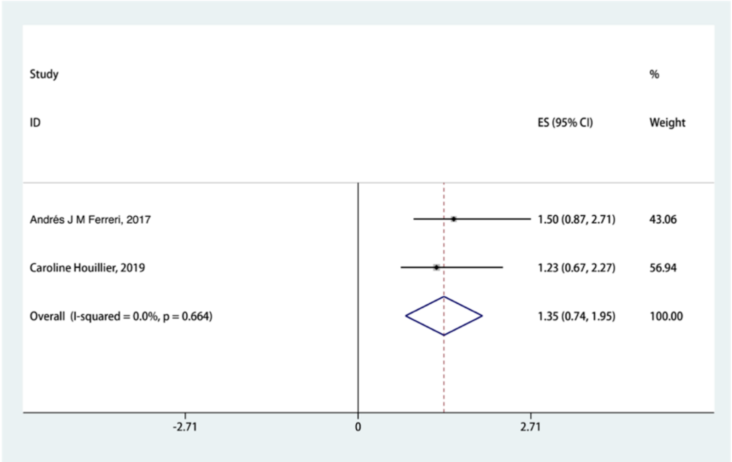

Supplementary Figure S2.7.2 Meta-analysis for 2-year PFS in patient with PCNSL (ASCT vs. WBRT).

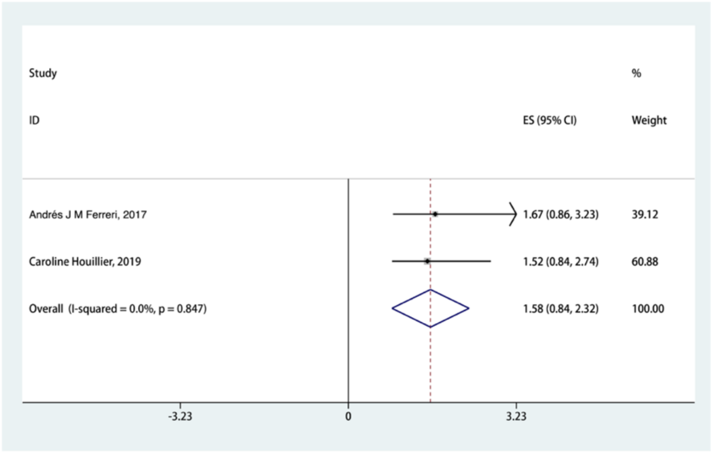

Supplementary Figure S2.7.3. Meta-analysis for 2-year OS in patient with PCNSL (ASCT vs. WBRT)

7.5. Summary of finding (SoF) tables

Outcomes	Anticipated absolute effects* (95% CI)		Relative effect (95% CI)	№ of participants (studies)	Certainty of the evidence (GRADE)	Comments
	Risk with [comparison]	Risk with [intervention]				
**OR**	889 per 1,000	933 per 1,000 (848 to 972)	**RR 1.75** (0.70 to 4.35)	291 (1RCT, 2 observational studies)	⨁◯◯◯ Low[a]	None
**2-year PFS**	NA	NA	**HR 1.35** (0.61 to 0.88)	250 (2 RCTs)	⨁⨁◯◯ Low[a,b]	None
**2-year OS**	NA	NA	**HR 1.58** (0.84 to 2.32)	250 (2 RCTs)	⨁⨁◯◯ Low[a,b]	None

*The risk in the intervention group (and its 95% confidence interval) is based on the assumed risk in the comparison group and the relative effect of the intervention (and its 95% CI). CI: Confidence interval; RR: Risk ratio; HR: Hazard ratio; NA: Not applicable.

Explanation

a. The sample size is lower than the optimal information sample size.
b. None of the RCTs were blinded.

7.6. References

[1] Ferreri AJM, Cwynarski K, Pulczynski E, et al. Whole-brain radiotherapy or autologous stem-cell transplantation as consolidation strategies after high-dose methotrexate-based chemoimmunotherapy in patients with primary CNS lymphoma: results of the second randomisation of the International Extranodal Lymphoma Study Group-32 phase 2 trial. The lancet Haematology. 2017;4(11):e510 - e23.

[2] Houillier C, Taillandier L, Dureau S, et al. Radiotherapy or Autologous Stem-Cell Transplantation for Primary CNS Lymphoma in Patients 60 Years of Age and Younger: Results o the Intergroup ANOCEF-GOELAMS Randomized Phase II PRECIS Study. Journal of Clinical Oncology. 2019;37(10): 823-833.

[3] Correa DD, Braun E, Kryza-Lacombe M, et al. Longitudinal cognitive assessment in patients with primary CNS lymphoma treated with induction chemotherapy followed by reduced-dose whole-brain radiotherapy or autologous stem cell transplantation. Journal of Neuro-Oncology. 2019;144(3):553-62.

[4] Ferreri AJM, Calimeri T, Ponzoni M, et al. Improving the antitumor activity of R-CHOP with NGR-hTNF in primary CNS lymphoma: final results of a phase 2 trial. Blood Adv. 2020;4(15):3648-58.

[5] Houillier C, Soussain C, Ghesquières H, et al. Management and outcome of primary CNS lymphoma in the modern era: An LOC network study. Neurology. 2020;94(10):e1027-e39.

[6] Kim JE, Yoon DH, Kim S, et al. Relapse pattern and prognostic factors for patients with primary central nervous system lymphoma. The Korean journal of hematology. 2012;47(1):60-6.

CQ 8: Should BTK inhibitors be used to treat patients with PCNSL?

- Population: Patients with PCNSL
- Intervention: BTK inhibitors
- Comparison: Other treatment, placebo

8.1. Inclusion and exclusion criteria

- inclusion criteria: we included published studies, which treated the localized recurrent refractory PCNSL with BTK inhibitors. Studies published in English and Chinese are included.
- exclusion criteria: we excluded studies where we failed to access full text, or studies without sufficient data.

8.2. Characteristic information of included studies

Supplementary Table S2.8.1. Characteristic information of included studies

Study ID	Country	Research type	Disease Stage	N	Intervention	Dosing method	ORR	CR	PR	PFS	OS
Yu 2021 [1]	China	Case report	newly diagnosed / relapsed or refractory	3	ibrutinib	single/combination	100% (3/3)	67%	33%	7m	9.3 m
Lewis 2021 [2]	Australia	Case series	relapsed or refractory	9	ibrutinib	single/combination	44% (4/9)	44%	/	31m	31m
Chen 2020 [3]	China	Case series	newly diagnosed	11	ibrutinib	ibrutinib+HD-MTX	82% (9/11)	64%	18%	7.4m	/
Grommes 2017 [4]	America	Case series	relapsed or refractory	13	ibrutinib	single/combination	77% (10/13)	38.5 %	38.5 %	4.6m	15m
Grommes 2019 [5]	America	Cohort study	relapsed or refractory	9	ibrutinib	combination	89% (8/9)	67%	22%	/	/
Chamoun 2017 [6]	French	Case series	relapsed or refractory	14	ibrutinib	single/combination	50% (7/14)	21%	28.5 %	/	/

Lionakis 2017 [7]	French	Case series	newly diagnosed / relapsed or refractory	18	ibrutinib	single/combination	94% (17/18)	88%	6%	11.2 m	/
Soussain 2019 [8]	French	Single-arm study	relapsed or refractory CNSL / PVRL	52	ibrutinib	single	52% (27/52)	19%	33%	4.8m	19.2 m
LAUER EM 2020 [9]	Germany	Single centre case series	relapsed or refractory	9	ibrutinib	single/combination	66%（6/9）	66%（6/9）	/	/	/
Grommes 2018 [10]	America	Single-arm study	relapsed or refractory	27	ibrutinib	single	81% (31/40)	/	/	/	/
Grommes 2019 [11]	America	Single-arm study	relapsed or refractory	6	ibrutinib	Ibrutinib + copanlisib	67% (4/6)	17%	50%	/	/
Dunleavy 2015 [12]	America	Cohort study	newly diagnosed / relapsed or refractory	11	ibrutinib	single/combination (ibrutinib + DA-TEDDI-R)	73% (8/11)	/	64%	/	/
Bairey 2019 [13]	Israel	Single-arm study	newly diagnosed / the elder	12	ibrutinib	Ibrutinib + HD-MTX	/	25%	/	22.5 m	/
Roschewski 2020 [14]	America	Single-arm study	relapsed or refractory	13	ibrutinib	combination	85% (11/13)	61.5 %	/	/	/

Lewis 2019 [15]	Australia	Case series	newly diagnosed / relapsed or refractory	8	ibrutinib	combination	50% (4/8)	/	/	/	/
Roschewski 2018 [16]	America	Cohort study	newly diagnosed / relapsed or refractory	18	ibrutinib	ibrutinib + TEDD-R	/	50%	/	15.2 m	/
Christian G 2015 [17]	America	Case series	relapsed or refractory	4	ibrutinib	single	50%(2/4)	/	/	/	/
Yuedan C 2020 [18]	China	Single-arm study	relapsed or refractory	18	ibrutinib	ibrutinib + I-MIDD regimen	83.3% (5/18)	55.5 %	27.8 %	6m	/
Narita 2021 [19]	Japan	non-randomized controlled study	relapsed or refractory	44	tirabrutinib	single	64% (28/44)	9.1% (4/44)	29.5 % (13/44)	4.9m	/
Hou K 2021 [20]	China	Systematic review	non-GCB DLBCL and relapsed/refractory CNSL	1145	ibrutinib	Single/combination (ibrutinib + RTX)	57.9 % (663/1145)	35.0 %	20.1 %	4.45 m	11.5 m

/: none

8.3. Risk of bias

Supplementary Table S2.8.2. Risk of bias of included non-randomized controlled studies assessed by the Newcastle-Ottawa Scale

Study ID	Selection of exposure				Comparability			Outcome		Total score
	Representativeness of the exposed cohort	Selection of the non-exposed cohort	Ascertainment of exposure to implants	Demonstration that outcome of interest was not present at start of study	Study controls the most important factor	Study controls for any additional factor	Assessment of outcome	Was follow up long enough for outcomes to occur	Adequacy of follow up of cohorts	
Grommes 2019 [5]	c	Yes	Yes	Yes	Yes	No	No	Yes	Yes	7
Dunleavy 2015 [12]	Yes	No	Yes	Yes	Yes	No	No	Yes	Yes	6
Roschewski 2018 [16]	Yes	Yes	No	Yes	Yes	No	No	Yes	Yes	6
Soussain [8]	Yes	No	Yes	Yes	Yes	No	No	Yes	Yes	6
LAUER EM [9]	Yes	Yes	Yes	Yes	Yes	Yes	Yes	Yes	Yes	4
Grommes [10]	Yes	Yes	Yes	Yes	Yes	No	No	Yes	Yes	7
Grommes [11]	Yes	Yes	Yes	Yes	Yes	No	No	Yes	Yes	7
Bairey [13]	Yes	No	Yes	No	No	Yes	Yes	No	Yes	5
Roschewski [14]	Yes	Yes	Yes	No	No	Yes	No	No	No	4
Yuedan C [18]	Yes	Yes	No	Yes	Yes	No	No	Yes	Yes	6
Narita[19]	Yes	Yes	Yes	Yes	Yes	No	No	Yes	Yes	7

Supplementary Table S2.8.3. Risk of bias of included case report and case series studies assessed by the Institute of Health Economics checklist

Study ID	Study objective	Study population					Intervention and co-intervention	
	Is the hypothesis, aim, objective of the study clearly stated?	Are the characteristics of the participants included in the study described?	Were the cases collected in more than one centre?	Are the eligibility criteria for entry into the study clearly stated?	Were participants recruited consecutively?	Did participants enter the study at a similar point in the disease?	Was the intervention of interest clearly described?	Were additional interventions (co-interventions) reported in the study?
Yu [1]	Yes	No	No	Unclear	Unclear	Unclear	No	Yes
Lewis [2]	Yes	Yes	Yes	Unclear	Unclear	No	Yes	Yes
Chen [3]	Yes	Yes	Yes	Unclear	Unclear	No	Yes	Yes
Grommes [4]	Yes	Yes	Yes	Unclear	Unclear	No	Yes	Yes
Chamoun [6]	Yes	Yes	Yes	Unclear	Unclear	No	Yes	Yes
Lionakis [7]	Yes	Yes	Yes	Unclear	Unclear	No	Yes	Yes
Lewis [15]	Yes	Yes	Yes	Unclear	Unclear	No	Yes	Yes

Continued Supplementary Table S2.8.3.

Study ID	Outcome measure			Statistical analysis	Results and conclusions	
	Are the outcome measures established a priori?	Were the relevant outcomes measured with appropriate objective and/or	Were the relevant outcomes measured before and after	Were the statistical tests used to assess the relevant outcomes	Was the length of follow-up reported?	Was the loss to follow-up reported?

		subjective methods?	the intervention?	appropriate?		
Yu [1]	Yes	Yes	No	Yes	No	No
Lewis [2]	Partial reported	Unclear	No	Unclear	No	No
Chen [3]	Yes	Yes	No	No	No	No
Grommes [4]	Yes	Yes	No	Yes	Unclear	No
Chamoun [6]	No	Yes	Yes	No	Unclear	Unclear
Lionakis [7]	No	Partial reported	Yes	No	Unclear	Unclear
Lewis [15]	No	Yes	Yes	No	No	Unclear

Continued Supplementary Table S2.8.3.

Study ID	Results and conclusions			Competing interest and source of support	New items		Total score
	Does the study provide estimates of the random variability in the data analysis of relevant outcomes?	Are the adverse events related with the intervention reported?	Are the conclusions of the study supported by results?	Are both competing interests and sources of support for the study reported?	Was the study conducted prospectively?	Were the relevant outcomes assessed blinded to intervention status?	
Yu [1]	No	No	Yes	Yes	Unclear	Unclear	10
Lewis [2]	No	No	Yes	Partial reported	No	Unclear	5
Chen [3]	No	No	Yes	Partial reported	Yes	Unclear	8
Grommes [4]	No	No	Yes	No	Yes	Unclear	9
Chamoun [6]	No	No	Unclear	Yes	Unclear	No	8

Lionakis [7]	Partial reported	Unclear	Unclear	Unclear	Yes	No	6
Lewis [15]	Yes	Yes	Unclear	Yes	No	No	8

Supplementary Table S2.8.4 Risk of bias of included systematic reviews assessed by the AMSTAR

Study ID	Was an 'a priori' design provided?	Was there duplicate study selection and data extraction?	Was a comprehensive literature search performed?	Was the status of publication (i.e., grey literature) used as an inclusion criterion?	Was a list of studies (included and excluded) provided?	Were the characteristics of the included studies provided?	Was the scientific quality of the included studies assessed and documented?	Was the scientific quality of the included studies used appropriately in formulating conclusions?	Were the methods used to combine the findings of studies appropriate?	Was the likelihood of publication bias assessed?	Was the likelihood of publication bias assessed?
Hou K [20]	No	Yes	Yes	Yes	No	Yes	Yes	Yes	Yes	No	Yes

8.4 Meta-analysis results

Study or Subgroup	Risk Difference	SE	Weight	Risk Difference IV, Random, 95% CI
Chamoun 2017	0.5	0.134	6.0%	0.50 [0.24, 0.76]
Chen(ca) 2020	0.833	0.088	9.0%	0.83 [0.66, 1.01]
Chen 2020	0.82	0.116	7.0%	0.82 [0.59, 1.05]
Dunleavy(ca) 2015	0.73	0.134	6.0%	0.73 [0.47, 0.99]
Grommes(ca) 2015	0.67	0.271	2.1%	0.67 [0.14, 1.20]
Grommes(ca) 2018	0.81	0.075	10.0%	0.81 [0.66, 0.96]
Grommes(ca) 2019	0.67	0.192	3.7%	0.67 [0.29, 1.05]
Grommes 2017	0.77	0.117	6.9%	0.77 [0.54, 1.00]
Grommes 2019	0.89	0.104	7.8%	0.89 [0.69, 1.09]
Lauer 2020	0.6	0.22	3.0%	0.60 [0.17, 1.03]
Lewis(ca) 2019	0.5	0.177	4.1%	0.50 [0.15, 0.85]
Lewis 2021	0.44	0.165	4.6%	0.44 [0.12, 0.76]
Lionakis 2017	0.94	0.056	11.6%	0.94 [0.83, 1.05]
Roschewski(ca) 2020	0.85	0.099	8.1%	0.85 [0.66, 1.04]
Soussain 2019	0.59	0.074	10.1%	0.59 [0.44, 0.74]
Yu 2021	1	0		Not estimable
**Total (95% CI)**			**100.0%**	**0.74 [0.66, 0.83]**

Heterogeneity: Tau² = 0.01; Chi² = 29.44, df = 14 (P = 0.009); I² = 52%
Test for overall effect: Z = 17.46 (P < 0.00001)

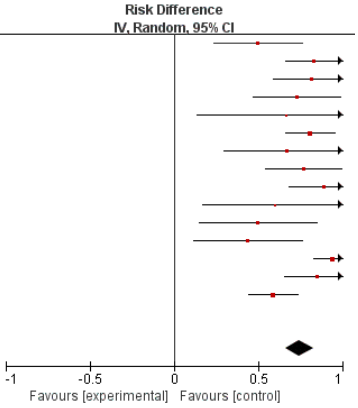

Supplementary Figure S2.8.1. Forest plot of overall efficiency of ibrutinib

Study or Subgroup	Risk Difference	SE	Weight	Risk Difference IV, Random, 95% CI
Bairey(ca) 2019	0.25	0.125	6.9%	0.25 [0.01, 0.49]
Chamoun 2017	0.21	0.109	7.4%	0.21 [-0.00, 0.42]
Chen(ca) 2020	0.555	0.117	7.1%	0.56 [0.33, 0.78]
Chen 2020	0.64	0.145	6.3%	0.64 [0.36, 0.92]
Dunleavy(ca) 2015	0.45	0.15	6.2%	0.45 [0.16, 0.74]
Grommes(ca) 2015	0.33	0.271	3.5%	0.33 [-0.20, 0.86]
Grommes(ca) 2019	0.17	0.153	6.1%	0.17 [-0.13, 0.47]
Grommes 2017	0.385	0.135	6.6%	0.39 [0.12, 0.65]
Grommes 2019	0.67	0.157	6.0%	0.67 [0.36, 0.98]
Lauer 2020	0.6	0.22	4.4%	0.60 [0.17, 1.03]
Lewis 2021	0.44	0.165	5.7%	0.44 [0.12, 0.76]
Lionakis 2017	0.86	0.082	8.1%	0.86 [0.70, 1.02]
Roschewski(ca) 2018	0.5	0.118	7.1%	0.50 [0.27, 0.73]
Roschewski(ca) 2020	0.615	0.135	6.6%	0.61 [0.35, 0.88]
Soussain 2019	0.23	0.063	8.6%	0.23 [0.11, 0.35]
Yu 2021	0.67	0.271	3.5%	0.67 [0.14, 1.20]
**Total (95% CI)**			**100.0%**	**0.47 [0.34, 0.59]**

Heterogeneity: Tau² = 0.04; Chi² = 55.67, df = 15 (P < 0.00001); I² = 73%
Test for overall effect: Z = 7.37 (P < 0.00001)

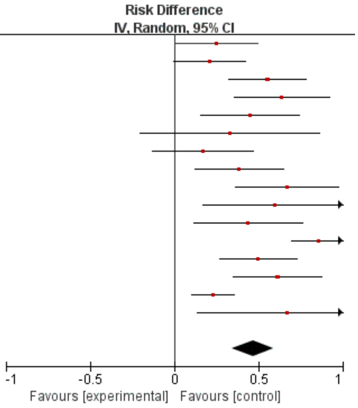

Supplementary Figure S2.8.2. Forest plot of complete remission rate of ibrutinib

Study or Subgroup	Risk Difference	SE	Weight	Risk Difference IV, Random, 95% CI
Chamoun 2017	0.285	0.121	10.1%	0.28 [0.05, 0.52]
Chen(ca) 2020	0.278	0.106	12.2%	0.28 [0.07, 0.49]
Chen 2020	0.18	0.116	10.7%	0.18 [-0.05, 0.41]
Grommes(ca) 2015	0.33	0.271	2.6%	0.33 [-0.20, 0.86]
Grommes(ca) 2019	0.5	0.204	4.3%	0.50 [0.10, 0.90]
Grommes 2017	0.385	0.135	8.6%	0.39 [0.12, 0.65]
Grommes 2019	0.22	0.138	8.3%	0.22 [-0.05, 0.49]
Lionakis 2017	0.08	0.064	21.5%	0.08 [-0.05, 0.21]
Soussain 2019	0.36	0.072	19.2%	0.36 [0.22, 0.50]
Yu 2021	0.33	0.271	2.6%	0.33 [-0.20, 0.86]
**Total (95% CI)**			**100.0%**	**0.26 [0.17, 0.35]**

Heterogeneity: Tau² = 0.01; Chi² = 12.58, df = 9 (P = 0.18); I² = 28%
Test for overall effect: Z = 5.75 (P < 0.00001)

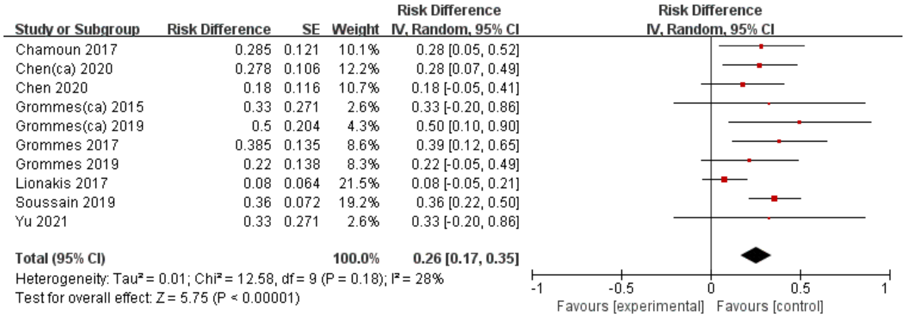

Supplementary Figure S2.8.3. Forest plot of partial response rate of ibrutinib

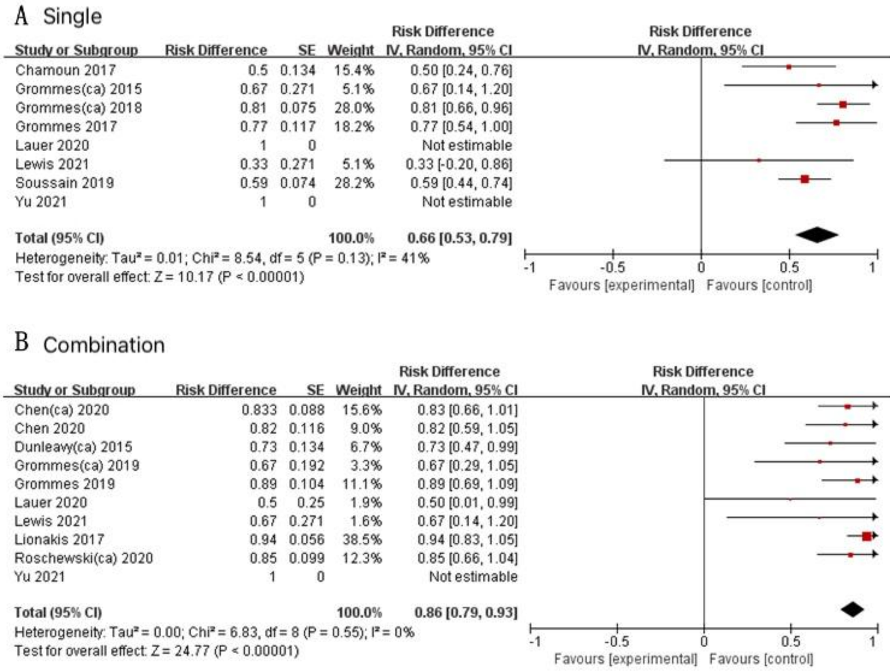

Supplementary Figure S2.8.4. Forest plot of overall efficiency of ibrutinib for PCNSL (A) alone (B) in combination

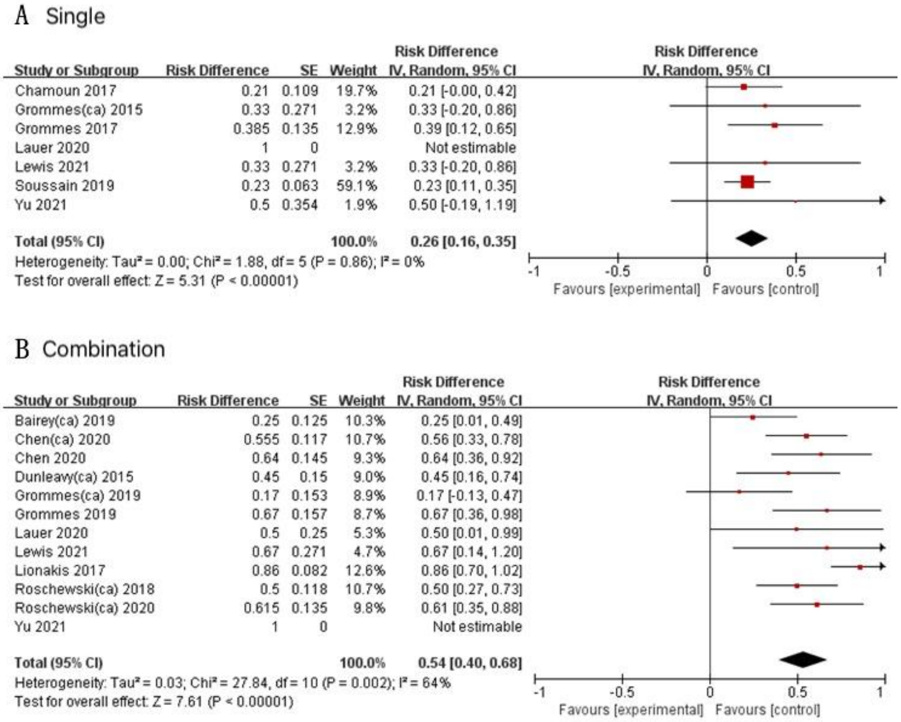

Supplementary Figure S2.8.5. Forest plot of complete remission rate of PCNSL treated with ibrutinib (A) alone (B) in combination

A Single

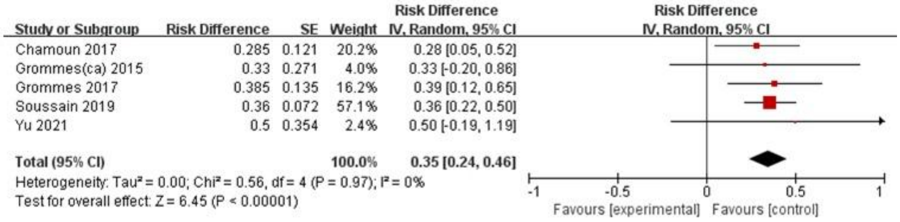

B Combination

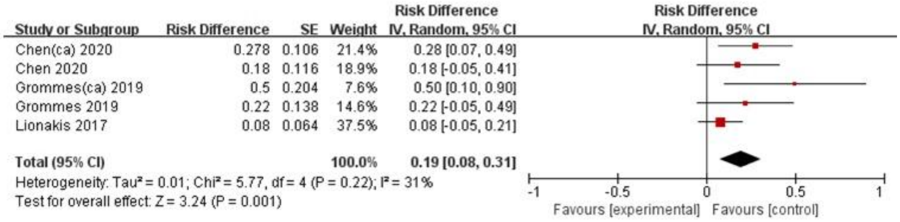

Supplementary Figure S2.8.6. Forest plot of partial remission rate of PCNSL treated with ibrutinib (A) alone (B) in combination

8.5. Summary of finding (SoF) tables

Outcomes	Anticipated absolute effects* (95% CI)		Relative effect (95% CI)	№ of participants (studies)	Certainty of the evidence (GRADE)	Comments
	Risk with [comparison]	Risk with [intervention]				
overall efficiency	-	-	-	215 (15 observational studies)	⊕⊕◯◯ LOW	
complete remission rate	-	-	-	199 (14 observational studies)	⊕⊕◯◯ LOW	
partial response rate	-	-	-	158 (11 observational studies)	⊕⊕◯◯ LOW	

***The risk in the intervention group** (and its 95% confidence interval) is based on the assumed risk in the comparison group and the **relative effect** of the intervention (and its 95% CI).
CI: confidence interval

GRADE Working Group grades of evidence
High certainty: we are very confident that the true effect lies close to that of the estimate of the effect.
Moderate certainty: we are moderately confident in the effect estimate: the true effect is likely to be close to the estimate of the effect, but there is a possibility that it is substantially different.
Low certainty: our confidence in the effect estimate is limited: the true effect may be substantially different from the estimate of the effect.
Very low certainty: we have very little confidence in the effect estimate: the true effect is likely to be substantially different from the estimate of effect.

Explanation
a. the risk of bias in many items is unclear; b. I^2=65; c. the simple size is under 300

8.6. Reference

[1] YU H, KONG H, LI C, et al. Bruton's tyrosine kinase inhibitors in primary central nervous system lymphoma-evaluation of anti-tumor efficacy and brain distribution [J]. TRANSLATIONAL CANCER RESEARCH, 2021, 10(5): 1975-83.

[2] Lewis K L, CHIN C K, MANOS K, et al. Ibrutinib for central nervous system lymphoma: the Australasian Lymphoma Alliance/MD Anderson Cancer Center experience [J]. Br J Haematol, 2021, 192(6): 1049-53.

[3] Chen F, Pang D, Guo H, et al. Clinical outcomes of newly diagnosed primary CNS lymphoma treated with ibrutinib - based combination therapy: A real - world experience of off - label ibrutinib use[J]. Cancer medicine, 2020, 9(22): 8676-8684.

[4] GROMMES C, PASTORE A, PALASKAS N, et al. Ibrutinib Unmasks Critical Role of Bruton Tyrosine Kinase in Primary CNS Lymphoma [J]. Cancer discovery, 2017, 7(9): 1018-1029.

[5] Grommes C, Tang S S, Wolfe J, et al. Phase 1b trial of an ibrutinib-based combination therapy in recurrent/refractory CNS lymphoma[J]. Blood, The Journal of the American Society of Hematology, 2019, 133(5): 436-445.

[6] CHAMOUN K, CHOQUET S, BOYLE E, et al. Ibrutinib monotherapy in relapsed/refractory CNS lymphoma: A retrospective case series [J]. Neurology, 2017, 88(1): 101-2.

[7] LIONAKIS M S, DUNLEAVY K, ROSCHEWSKI M, et al. Inhibition of B Cell Receptor Signaling by Ibrutinib in Primary CNS Lymphoma [J]. Cancer cell, 2017, 31(6): 833-43.e5.

[8] Soussain C, Choquet S, Blonski M, et al. Ibrutinib monotherapy for relapse or refractory primary CNS lymphoma and primary vitreoretinal lymphoma: final analysis of the phase II ‘proof-of-concept’iLOC study by the Lymphoma study association (LYSA) and the French oculo-cerebral lymphoma (LOC) network[J]. European journal of cancer, 2019, 117: 121-130.

[9] LAUER EM, WATERHOUSE M, BRAIG M, et al. Ibrutinib in patients with relapsed/refractory central nervous system lymphoma: A retrospective single-centre analysis [J]. Br J Haematol, 2020, 190(2): e110-e4.

[10] Grommes C, Wolfe J, Gavrilovic I, et al. Phase II of single-agent ibrutinib in recurrent/refractory primary (PCNSL) and secondary CNS lymphoma (SCNSL)[J]. Blood, 2018, 132: 2965.

[11] Grommes C, Gavrilovic I, Miller A M, et al. Phase Ib of copanlisib in combination with ibrutinib in recurrent/refractory primary CNS lymphoma (PCNSL)[J]. Blood, 2019, 134: 1598.

[12] Dunleavy K, Lai C E, Roschewski M, et al. Phase I study of dose-adjusted-Teddi-R with ibrutinib in untreated and relapsed/refractory primary CNS lymphoma[J]. Blood, 2015, 126(23): 472.

[13] Bairey O, Amiel A, Yust-Katz S, et al. P05. 04 Phase 2 open-label study of maintenance treatment with ibrutinib following first line methotrexate-based immuno-chemotherapy in elderly patients with primary CNS lymphoma (PCNSL)[J]. Neuro-Oncology, 2019, 21(Suppl 3): iii34.
[14] Roschewski M, Melani C, Lakhotia R, et al. Phase 1 study of escalating doses of ibrutinib and temozolomide, etoposide, liposomal doxorubicin, dexamethasone, rituximab (TEDDI-R) with isavuconazole for relapsed and refractory primary CNS lymphoma[J]. Blood, 2020, 136: 12-13.
[15] Lewis K L, Manos K, Casey J, et al. Outcomes for Patients with Primary or Secondary Central System Lymphoma Treated with Ibrutinib: A Multicentre Retrospective Analysis[J]. Blood, 2019, 134: 1620.
[16] Roschewski M, Lionakis M S, Melani C, et al. Dose-adjusted teddi-R induces durable complete remissions in relapsed and refractory primary CNS lymphoma[J]. Blood, 2018, 132: 4195.
[17] Christian G, Thomas K, Abdel-Wahab O, et al. Phase I Study of Single Agent Ibrutinib in Recurrent/Refractory Primary and Secondary CNS Lymphoma[J]. Blood, 2015, 126(23): 3960.
[18] Yuedan C, Sun X, Bai X, et al. Preliminary Exploration of Ibrutinib Combined with Methotrexate, Ifosfamide, Liposomal Doxorubicin and Methylprednisolone in the Treatment of Relapsed/Refractory Primary CNS Lymphoma[J]. Blood, 2020, 136: 13-14.
[19] Narita, Y., Nagane, M., Mishima, K., et al., Phase I/II study of tirabrutinib, a second-generation Bruton's tyrosine kinase inhibitor, in relapsed/refractory primary central nervous system lymphoma[J]. **Neuro Oncol,** 2021. 23(1): p. 122-133.
[20] Hou K, Yu Z, Jia, Y, et al., Efficacy and safety of ibrutinib in diffuse large B-cell lymphoma: A single-arm meta-analysis[J]. **Crit Rev Oncol Hematol,** 2020. 152: p. 103010.

CQ 9: Should stereotactic radiosurgery be used to treat localized recurrent PCNSL patients who were refractory to chemotherapy and previously received WBRT?

- Population: Patients with localized recurrent PCNSL who were refractory to chemotherapy and previously received WBRT
- Intervention: Treat with stereotactic radiosurgery (SRS)
- Comparison: Other treatment

9.1. Inclusion and exclusion criteria

- inclusion criteria: we included published studies, which treated the localized recurrent refractory PCNSL with SRS. Studies published in English and Chinese are included.
- exclusion criteria: we excluded studies where we failed to access full text, or studies without sufficient data.

9.2. Characteristic information of included studies

Supplementary Table S2.9.1. Characteristic information of included studies

Study ID	Publication year	Country	N	Population	Study type	Lesion	Volume of tumors	Dosage	Other treatment	CR	PR	SD+PD	mPFS	mOS	Neurovirulence
Matsumoto et al [1]	2007	Japanese	2	recurrent PCNSL	case report	Patient 1: Single lesion (38.4*39.1*30.0 mm with a volume of 24.6 mL)	NR	Center dose 30Gy Edge dose 15Gy	NR	NR	NR	NR	3m	13m	NR
						Patient 2: Multiple lesions	NR	NR	NR	NR	NR	NR	13m	15m	NR
Kenai et al [2]	2006	Sweden	22 (4 initial, 18 recurrent)	PCNSL	case series	16.2 mm (3.24 mm-42.4 mm)	4.14cm^3 (0.02cm^3 - 39.9cm^3)	Maximum dose 38.5 Gy Edge dose	NR	NR	NR	NR	32.1m (6m - 67 m)	NR	0 (0%)

Sakamoto et al [3]	2006	Japanese	9	recurrent PCNSL	case report	NR	3.5mL(0.4-24.5mL)	16.5 Gy Minimum dose 9.1Gy Maximum dose 15.2 Gy	NR	8(88.9%)	1(11.1%)	NR	7.7 m	0 (0%)
Shin et al [4]	2017	America	23 (7 initial, 16 recurrent)	PCNSL	case series	NR	4cm3(0.1cm3-26cm3)	NR	NR	NR NR	NR	NR	11 m (5.7-33.2m)	0 (0%)
Kumar et al [5]	2015	America	14 (7 initial, 7 recurrent)	Intracranial recurrent lymphoma	case series	NR	6.7cm3(0.5cm3-37.7cm3)	Edge dose 15.5 Gy Maximum dose 32Gy	NR	11 (78.6%)	NR	3.3 m	9.5 m (0.4 m-94 m)	0 (0%)

NR: Not report; CR: Complete Response; PR: Partial Response; SD+PD: Stable Disease + Progressive Disease; mPFS: median Progression-Free Survival; mOS: median Overall Survival.

9.3. Risk of bias

Supplementary Table 2.9.2. Risk of bias of included case series assessed by the Institute of Health Economics checklist

Study ID	Study objective	Study population					Intervention and co-intervention	
	Is the hypothesis, aim, objective of the study clearly stated?	Are the characteristics of the participants included in the study described?	Were the cases collected in more than one centre?	Are the eligibility criteria for entry into the study clearly stated?	Were participants recruited consecutively?	Did participants enter the study at a similar point in the disease?	Was the intervention of interest clearly described?	Were additional interventions (co-interventions) reported in the study?
Matsumoto et al,	Yes	Yes	No	Yes	Unclear	No	Yes	Yes
Kenai et al.	Yes	Yes	No	No	Yes	No	Yes	Unclear
Sakamoto et al.	No	Yes	No	No	Yes	No	Yes	No
Shin et al.	Yes	Yes	Yes	Partial reported	Yes	No	Yes	Unclear
Kumar et al.	Yes	Yes	No	Partial reported	Unclear	Unclear	Yes	Unclear

Continued Supplementary Table S2.9.2.

Study ID	Outcome measure			Statistical analysis	Results and conclusions	
	Are the outcome measures established a priori?	Were the relevant outcomes measured with	Were the relevant outcomes measured before and	Were the statistical tests used to assess the relevant	Was the length of follow-up reported?	Was the loss to follow-up

		appropriate objective and/or subjective methods?	after the intervention?	outcomes appropriate?		reported?
Matsumoto et al,	No	No	Unclear	Yes	Unclear	No
Kenai et al.	Partial reported	Unclear	Unclear	Unclear	Yes	No
Sakamoto et al.	Yes	Yes	Unclear	Yes	No	No
Shin et al.	Yes	Yes	Unclear	Yes	Yes	No
Kumar et al.	Partial reported	Unclear	Unclear	Yes	Yes	No

Continued Supplementary Table S2.9.2

Study ID	Results and conclusions			Competing interest and source of support	New items		Total score
	Does the study provide estimates of the random variability in the data analysis of relevant outcomes?	Are the adverse events related with the intervention reported?	Are the conclusions of the study supported by results?	Are both competing interests and sources of support for the study reported?	Was the study conducted prospectively?	Were the relevant outcomes assessed blinded to intervention status?	
Matsumoto et al,	No	Yes	Yes	No	Unclear	Unclear	8
Kenai et al.	No	Partial reported	Yes	No	Unclear	Unclear	6

Sakamoto et al.	No	Yes	Yes	No	No	Unclear	8
Shin et al.	Partial reported	Yes	Yes	Yes	Unclear	Unclear	12
Kumar et al.	Yes	Yes	Yes	Partial reported	No	Unclear	8

9.4. Reference

[1] Matsumoto Y, Horiike S, Fujimoto Y, et al. Effectiveness and limitation of gamma knife radiosurgery for relapsed central nervous system lymphoma: a retrospective analysis in one institution. International Journal of Hematology. 2007;85(4):333-337.

[2] Kenai H, Yamashita M, Nakamura T, et al. Gamma Knife surgery for primary central nervous system lymphoma: usefulness as palliative local tumor control. Journal of Neurosurgery. 2006;105 Suppl:133-138.

[3] Sakamoto M, Oya N, Mizowaki T, et al. Initial experiences of palliative stereotactic radiosurgery for recurrent brain lymphomas. Journal of Neuro-Oncology. 2006;77(1):53-58.

[4] Shin SM, Silverman JS, Bowden G, et al. Relapsed or refractory primary central nervous system lymphoma radiosurgery: Report of the International Gamma Knife Research Foundation. Journal of Radiosurgery and SBRT. 2017;4(4):247-253.

[5] Kumar R, Laack N, Pollock BE, et al. Stereotactic Radiosurgery in the Treatment of Recurrent CNS Lymphoma. World Neurosurgery. 2015;84(2):390-397.

CQ 10. Which is the preferred approach to make the diagnosis of a suspected PVRL, vitreous biopsy or aqueous humor/vitreous puncture?

- Population: Patients with PVRL
- Intervention: Diagnosis with vitreous biopsy
- Comparison: Diagnosis with aqueous humor/vitreous puncture

10.1. Inclusion and exclusion criteria

- inclusion criteria: we included published studies, which compared the diagnosis sensitivity and specificity for a suspected PVRL patient between vitreous biopsy or aqueous humor/vitreous puncture.
- exclusion criteria: we excluded studies where we failed to access full text, or studies without sufficient data.

10.2. Characteristic information of included studies

Supplementary Table S2.10.1. Characteristic information of included studies

Study ID	Publication year	Country	N	Population	Study type	Paired-samples	Aqueous humor/vitreous puncture			Vitreous biopsy		
							Diagnosis methods	Sample numbers	Positive numbers	Diagnosis methods	Sample numbers	Positive numbers
Oahalou A et al [1]	2014	Netherland	75 (84 eyes)	suspected patients with uveitis	case series	Yes	cytologic-testing	53 eyes	0	cytologic-testing plus flow cytometry	53 eyes	1 eye
Hiemcke-Jiwa LS et al [2]	2018	Netherland	23 (28 eyes)	patients with VRL	cohort study	Yes	polymerase chain reaction (MYD88 L265P)	11 (12 eyes)	8 (8 eyes)	cytologic-testing plus flow cytometry	11 (12 eyes)	7 (8 eyes)
						No	polymerase chain reaction (MYD88 L265P)	10 (15 eyes)	2 (3 eyes)	cytologic-testing plus flow cytometry	8 (9 eyes)	4 (4 eyes)
Dalal M et al [3]	2014	America	27	patients with VRL	case series	No	cytology, microdissection plus molecular analysis	3	2	cytology, microdissection plus molecular analysis	19	15
Miserocchi E et al [4]	2019	Italy	8 (16 eyes)	patients with VRL	case series	No	polymerase chain reaction (MYD88 L265P)	8 (15 eyes)	6 (8 eyes)	cytologic-testing	8 (10 eyes)	7 (8 eyes)

Cassoux N et al [5]	2007	France	167	suspected patients with uveitis	case series	No	molecular analysis	45	40	cytologic-testing plus flow cytometry	51	47

10.3. Risk of bias

Supplementary Table S2.10.2. Risk of bias of included cohort studies assessed by the Newcastle-Ottawa Scale

Study ID	Selection of exposure				Comparability			Outcome		Total score
	Representativeness of the exposed cohort	Selection of the non-exposed cohort	Ascertainment of exposure to implants	Demonstration that outcome of interest was not present at start of study	Study controls the most important factor	Study controls for any additional factor	Assessment of outcome	Was follow up long enough for outcomes to occur	Adequacy of follow up of cohorts	
Hiemcke-Jiwa LS et al	Yes	Yes	Yes	Yes	No	No	Yes	Yes	Yes	7

Supplementary Table S2.10.3. Risk of bias of included case series assessed by the Institute of Health Economics checklist

Study ID	Study objective	Study population					Intervention and co-intervention	
	Is the hypothesis, aim, objective of the study clearly stated?	Are the characteristics of the participants included in the study described?	Were the cases collected in more than one centre?	Are the eligibility criteria for entry into the study clearly stated?	Were participants recruited consecutively?	Did participants enter the study at a similar point in the disease?	Was the intervention of interest clearly described?	Were additional interventions (co-interventions) reported in the study?
Oahalou A et al	Yes	Yes	No	Yes	Yes	Unclear	Yes	Unclear
Dalal M et al	Yes	Yes	No	Partial reported	Yes	Unclear	Yes	Unclear

Miserocchi E et al	Yes	Yes	Unclear	Partial reported	Yes	Unclear	Yes	Unclear
Cassoux N et al	Yes	Yes	No	Partial reported	Yes	Unclear	Yes	Unclear

Continued Supplementary Table 2.10.3.

**Study ID**	**Outcome measure**			**Statistical analysis**	**Results and conclusions**	
	**Are the outcome measures established a priori?**	**Were the relevant outcomes measured with appropriate objective and/or subjective methods?**	**Were the relevant outcomes measured before and after the intervention?**	**Were the statistical tests used to assess the relevant outcomes appropriate?**	**Was the length of follow-up reported?**	**Was the loss to follow-up reported?**
Oahalou A et al	Yes	Yes	No	Yes	No	No
Dalal M et al	Partial reported	Unclear	No	Unclear	No	No
Miserocchi E et al	Yes	Yes	No	No	No	No
Cassoux N et al	Yes	Yes	No	Yes	Unclear	No

Continued Supplementary Table S2.10.3.

**Study ID**	**Results and conclusions**			**Competing interest and source of support**	**New items**		**Total score**
	**Does the study provide estimates of the random variabilit**	**Are the adverse events related with the interventi**	**Are the conclusions of the study supported by results?**	**Are both competing interests and sources of support for the study reported?**	**Was the study conducted prospectively?**	**Were the relevant outcomes assessed blinded to interventi**	

	y in the data analysis of relevant outcomes?	on reported?				on status?	
Oahalou A et al	No	No	Yes	Yes	Unclear	Unclear	10
Dalal M et al	No	No	Yes	Partial reported	No	Unclear	5
Miserocchi E et al	No	No	Yes	Partial reported	Yes	Unclear	8
Cassoux N et al	No	No	Yes	No	Yes	Unclear	9

10.4. Reference

[1] Oahalou A, Schellekens PA, de Groot-Mijnes JD, et al. Diagnostic pars plana vitrectomy and aqueous analyses in patients with uveitis of unknown cause. Retina. 2014;34(1):108-114.

[2] Hiemcke-Jiwa LS, Ten DN, Leguit RJ, et al. Potential Diagnosis of Vitreoretinal Lymphoma by Detection of MYD88 Mutation in Aqueous Humor with Ultrasensitive Droplet Digital Polymerase Chain Reaction. JAMA Ophthalmology. 2018;136(10):1098-1104.

[3] Dalal M, Casady M, Moriarty E, et al. Diagnostic procedures in vitreoretinal lymphoma. Ocular Immunology and Inflammation. 2014;22(4):270-276.

[4] Miserocchi E, Ferreri A, Giuffrè C, et al. MYD88 L265P MUTATION DETECTION IN THE AQUEOUS HUMOR OF PATIENTS WITH VITREORETINAL LYMPHOMA. Retina. 2019;39(4):679-684.

Cassoux N, Giron A, Bodaghi B, et al. IL-10 measurement in aqueous humor for screening patients with suspicion of primary intraocular lymphoma. Investigative Ophthalmology & Visual Science. 2007;48(7):3253-3259.

CQ 11: Which is the preferred approach to treat PVRL patients and PCNSL patients with concurrent VRL, systemic therapy, local therapy, or combined systemic and local therapy?

- Population: PVRL patients and PCNSL patients with concurrent VRL
- Intervention: systemic treatment
- Comparison: local treatment, or combined systemic and local treatment

11.1. Inclusion and exclusion criteria

- inclusion criteria: we included published studies, which compared the systemic treatment, local treatment, or combined systemic and local treatment for PVRL patients and PCNSL patients with concurrent VRL.
- exclusion criteria: we excluded studies where we failed to access full text, or studies without sufficient data.

11.2. Characteristic information of included studies

Supplementary Table S2.11.1. Characteristic information of included studies

Study ID	Country	N	Age	Systemic treatment	Local treatment	Combined systemic and local treatment	Follow time
Castellino, et al 2019[1]	United States	69	65（36-85）	34	19	15	33.6（95% CI：1.2-175.2）
Klimova, et al 2018[2]	Czech Republic	20	61（48-77）	1	3	16	66（14-166）
Akiyama, et al 2016[3]	Japan	10	68.5 (46–78)	-	8	10	29.5
Grimm, et al 2008[4]	16 centers in 7 countries	221	60	74	-	106	36
Jahnke, et al 2006[5]	Germany	22	64（38-83）	13	9	-	10.25
Grimm, et al 2007[6]	16 centers in 7 countries	83	63（24-85）	-	23	53	32
Riemens, et al 2015[7]	17 centers in Europe	78	58（39-86）	40	30	17	49

“-“: not applicable

11.3 Risk of bias

Supplementary Table S2.11.2. Risk of bias of included cohort studies assessed by the Newcastle-Ottawa Scale

Study ID	Selection of exposure				Comparability		Outcome			Total score
	Representativeness of the exposed cohort	Selection of the non-exposed cohort	Ascertainment of exposure to implants	Demonstration that outcome of interest was not present at start of study	Study controls the most important factor	Study controls for any additional factor	Assessment of outcome	Was follow up long enough for outcomes to occur	Adequacy of follow up of cohorts	
Castellino, et al 2019[1]	Yes	Yes	Yes	No	No	No	Yes	Yes	Yes	6
Klimova, et al 2018[2]	Yes	Yes	Yes	No	No	No	Yes	Yes	Yes	6
Grimm, et al 2008[4]	Yes	Yes	Yes	No	No	No	Yes	Yes	Yes	6
Jahnke, et al 2006[5]	Yes	Yes	Yes	No	No	No	Yes	Yes	Yes	6
Grimm, et al 2007[6]	Yes	Yes	Yes	No	No	No	Yes	Yes	Yes	6
Riemens, et al 2015[7]	Yes	Yes	Yes	No	No	No	Yes	Yes	Yes	6

Supplementary Table S2.11.3. National Institutes of Health (NIH) Study Quality Assessment Tools for Before-After (Pre-Post) Studies with No Control Group

**Item**	**Akiyama, et al 2016[3]**
1. Was the study question or objective clearly stated?	Yes
2. Were eligibility/selection criteria for the study population prespecified and clearly described?	Yes
3. Were the participants in the study representative of those who would be eligible for the test/service/intervention in the general or clinical population of interest?	Yes
4. Were all eligible participants that met the prespecified entry criteria enrolled?	Yes
5. Was the sample size sufficiently large to provide confidence in the findings?	Not reported
6. Was the test/service/intervention clearly described and delivered consistently across the study population?	Yes
7. Were the outcome measures prespecified, clearly defined, valid, reliable, and assessed consistently across all study participants?	Yes
8. Were the people assessing the outcomes blinded to the participants' exposures/interventions?	Not reported
9. Was the loss to follow-up after baseline 20% or less? Were those lost to follow-up accounted for in the analysis?	Yes
10. Did the statistical methods examine changes in outcome measures from before to after the intervention? Were statistical tests done that provided p values for the pre-to-post changes?	Yes
11. Were outcome measures of interest taken multiple times before the intervention and multiple times after the intervention (i.e., did they use an interrupted time-series design)?	Yes
12. If the intervention was conducted at a group level (e.g., a whole hospital, a community, etc.) did the	Yes

statistical analysis take into account the use of individual-level data to determine effects at the group level?	
**Quality Rating**	**Good**

11.4. Reference

[1] Castellino A, Pulido JS, Johnston PB, et al. Role of systemic high-dose methotrexate and combined approaches in the management of vitreoretinal lymphoma: A single center experience 1990-2018. Am J Hematol. 2019;94(3):291-298. doi:10.1002/ajh.25350.

[2] Klimova A, Heissigerova J, Rihova E, et al. Combined treatment of primary vitreoretinal lymphomas significantly prolongs the time to first relapse. Br J Ophthalmol. 2018;102(11):1579-1585. doi:10.1136/bjophthalmol-2017-311574.

[3] Akiyama H, Takase H, Kubo F, et al. High-dose methotrexate following intravitreal methotrexate administration in preventing central nervous system involvement of primary intraocular lymphoma. Cancer Sci. 2016;107(10):1458-1464. doi:10.1111/cas.13012.

[4] Grimm SA, McCannel CA, Omuro AM, et al. Primary CNS lymphoma with intraocular involvement: International PCNSL Collaborative Group Report. Neurology. 2008;71(17):1355-1360. doi:10.1212/01.wnl.0000327672.04729.8c.

[5] Jahnke K, Korfel A, Komm J, et al. Intraocular lymphoma 2000-2005: results of a retrospective multicentre trial. Graefes Arch Clin Exp Ophthalmol. 2006;244(6):663-669. doi:10.1007/s00417-005-0138-9.

[6] Grimm SA, Pulido JS, Jahnke K, et al. Primary intraocular lymphoma: an International Primary Central Nervous System Lymphoma Collaborative Group Report. Ann Oncol. 2007;18(11):1851-1855. doi:10.1093/annonc/mdm340.

[7] Riemens A, Bromberg J, Touitou V, et al. Treatment strategies in primary vitreoretinal lymphoma: a 17-center European collaborative study. JAMA Ophthalmol. 2015;133(2):191-197. doi:10.1001/jamaophthalmol.2014.4755.
