# Supplementary material for: Evidence-based expert consensus on the management of primary central nervous system lymphoma in China
Source: J Hematol Oncol. 2022 Sep 29;15:136. doi: 10.1186/s13045-022-01356-7 (PMC9524012; doi:10.1186/s13045-022-01356-7)
Supplement: Supplementary file 4 — Additional file 4. Delphi results. [file 13045_2022_1356_MOESM4_ESM.pdf]

Additional file 4. Delphi results

The evidence evaluation group drafted preliminary recommendations for each CQ based on the results of systematic reviews and summary of findings tables. Then, expert consensus group was invited to vote on all the recommendations and definitions of terms and propose revision comments. A recommendation was deemed as reached consensus if most participants agreed (defined as ⩾ 75% agreement), otherwise, the recommendation would be entering the second round after revision.

Supplementary Table S3.1 Delphi consensus on recommendations

Clinical Questions and recommendations	Round 1	Round 2
**CQ 1: Which is the preferred approach to obtain the pathology specimen in patients with PCNSL, stereotactic brain biopsy or resection?**		
● Recommendation 1	agree: 100% (14/14)<br>disagree: 0<br>unsure: 0<br>**Reached consensus**	/
**CQ 2: Should corticosteroids be withdrawn from patients with suspected PCNSL/PVRL before biopsy?**		
● Recommendation 2.1	agree: 92.9% (13/14)<br>disagree: 0<br>unsure: 7.1% (1/14)<br>**Reached consensus**	/
● Recommendation 2.2	agree: 84.6% (11/13)<br>disagree: 0<br>unsure: 15.4% (2/13)<br>**Reached consensus**	/
**CQ 3: Which is the preferred imaging examination for PCNSL patients, MRI or whole-body PET-CT?**		
● Recommendation 3.1	agree: 100% (14/14)<br>disagree: 0<br>unsure: 0<br>**Reached**	/

	**consensus**	
● Recommendation 3.2	agree: 75.0% (9/12)<br>disagree: 8.3% (1/12)<br>unsure: 16.7%% (2/12)<br>**Reached consensus**	**/**
**CQ 4: Should cognitive function assessment be used for PCNSL patients?**		
● Recommendation 4	agree: 92.9% (13/14)<br>disagree: 0<br>unsure: 7.1% (1/14)<br>**Reached consensus**	**/**
**CQ 5: Which regimen is preferred to be combined with HD-MTX backbone in induction therapy?**		
● Recommendation 5	agree: 76.9% (10/13)<br>disagree: 0<br>unsure: 23.1% (3/13)<br>**Reached consensus**	**/**
**CQ 6: Should rituximab be used to treat newly-diagnosed PCNS-DLBCL patients in induction therapy?**		
● Recommendation 6	agree: 71.4% (10/14)<br>disagree: 0<br>unsure: 28.6% (4/14)<br>**Entering the second round**	agree: 78.6% (11/14)<br>disagree: 0<br>unsure: 21.4% (3/14)<br>**Reached consensus**
**CQ 7: Which is the preferred consolidation therapy for patients with PCNSL at consolidation therapy, whole-brain radiotherapy (WBRT) or autologous hematopoietic stem cell transplantation (ASCT)?**		
● Recommendation 7	agree: 92.9% (13/14)<br>disagree: 0<br>unsure: 7.1% (1/14)<br>**Reached**	**/**

	**consensus**	
**CQ 8: Should BTK inhibitors be used to treat patients with PCNSL?**		
● Recommendation 8	agree: 85.7% (12/14)<br>disagree: 0<br>unsure: 14.3% (2/14)<br>**Reached consensus**	/
**CQ 9: Should stereotactic radiosurgery be used to treat localized recurrent PCNSL patients who were refractory to chemotherapy and previously received WBRT?**		
● Recommendation 9	agree: 78.6% (11/14)<br>disagree: 0<br>unsure: 21.4% (3/14)<br>**Reached consensus**	/
**CQ 10: Which is the preferred approach to make the diagnosis of a suspected PVRL, vitreous biopsy or aqueous humor/vitreous puncture?**		
● Recommendation 10	agree: 92.9% (13/14)<br>disagree: 0<br>unsure: 7.1% (1/14)<br>**Reached consensus**	/
**CQ 11: Which is the preferred approach to treat PVRL patients and PCNSL patients with concurrent VRL, systemic therapy, local therapy, or combined systemic and local therapy?**		
● Recommendation 11	agree: 100% (14/14)<br>disagree: 0<br>unsure: 0<br>**Reached consensus**	/

Supplementary Table S3.2 Delphi consensus on definitions of essential terms

Terms	Round 1	Round 2
**primary central nervous system lymphoma, PCNSL**	agree: 92.3% (12/13)<br>disagree: 0<br>unsure: 7.7% (1/13)<br>**Reached consensus**	
Reference: Fox C P, Phillips E H, Smith J, et al. Guidelines for the diagnosis and management of primary central nervous system diffuse large B-cell lymphoma[J]. British journal of haematology, 2019, 184(3): 348-363.		
**Diffuse large B cell lymphoma, DLBCL**	agree: 100% (13/13)<br>disagree: 0<br>unsure: 0<br>**Reached consensus**	
Reference: Swerdlow S H, Campo E, Harris N. WHO Classification of Tumours of Haematopoietic and Lymphoid Tissues In: Bosman FT, Jaffe ES, Lakhani SR, Ohgaki H, eds[J]. World Health Organization Classification of Tumours Revised 4th Edition ed. Lyon, France: International Agency for Research on Cancer (IARC), 2017.		
**Primary vitreoretinal lymphoma, PVRL**	agree: 100% (13/13)<br>disagree: 0<br>unsure: 0<br>**Reached consensus**	
Reference: Chan C C, Sen H N. Current concepts in diagnosing and managing primary vitreoretinal (intraocular) lymphoma[J]. Discovery medicine, 2013, 15(81): 93.		
**Whole brain radiotherapy, WBRT）**	agree: 92.3% (12/13)<br>disagree: 0<br>unsure: 7.7% (1/13)<br>**Reached consensus**	
Reference: https://www.mayoclinic.org/diseases-conditions/brain-metastases/diagnosis-treatment/drc-20350140		

**Autologous hematopoetic stem cell transplantation, ASCT**	agree: 100% (13/13)<br>disagree: 0<br>unsure: 0<br>**Reached consensus**	
Reference: Cao L, Ding K, Song H, et al. Efficacy and influencing factors of autologous hematopoietic stem cell transplantation in the treatment of malignant lymphoma [J]. Chinese Journal of Tissue Engineering Research,2021,25(13):1993-1998.		
**Induction therapy**	agree: 92.3% (12/13)<br>disagree: 0<br>unsure: 7.7% (1/13)<br>**Reached consensus**	
Reference: Canadian Cancer Society. Induction treatments for acute myelogenous leukemia. https://www.cancer.ca/en/cancer-information/cancer-type/leukemia-acute-myelogenous-aml/treatment/induction/?region=nl		
**Consolidation therapy**	agree: 92.3% (12/13)<br>disagree: 0<br>unsure: 7.7% (1/13)<br>**Reached consensus**	
Reference: National Cancer Institute. https://www.cancer.gov/publications/dictionaries/cancer-terms/def/consolidation-therapy		
**Maintenance therapy**	agree: 92.3% (12/13)<br>disagree: 0<br>unsure: 7.7% (1/13)<br>**Reached consensus**	
Reference: National Cancer Institute. https://www.cancer.gov/publications/dictionaries/cancer-terms/def/maintenance-therapy		
**Multimodal tomography-guided biopsy**	agree: 100% (12/12)<br>disagree: 0<br>unsure: 0	

	**Reached consensus**	
Reference: Abi-Jaoudeh N, Kruecker J, Kadoury S, et al. Multimodality image fusion–guided procedures: technique, accuracy, and applications[J]. Cardiovascular and interventional radiology, 2012, 35(5): 986-998.		
**High-dose methotrexate, HD-MTX**	agree: 61.5% (8/13)<br>disagree: 7.7% (1/13)<br>unsure: 30.8% (4/13)<br>**Entering the second round**	agree: 92.9% (13/14)<br>disagree: 0<br>unsure: 7.1% (1/14)<br>**Reached consensus**
Reference: Zhu J J, Gerstner E R, Engler D A, et al. High-dose methotrexate for elderly patients with primary CNS lymphoma[J]. Neuro-oncology, 2009, 11(2): 211-215.		
**Progress free survival，PFS**	agree: 92.3% (12/13)<br>disagree: 0<br>unsure: 7.7% (1/13)<br>**Reached consensus**	
Reference: National Cancer Institute. https://www.cancer.gov/publications/dictionaries/cancer-terms/def/progression-free-survival?redirect=true		
**Overall survival, OS**	agree: 100% (13/13)<br>disagree: 0<br>unsure: 0<br>**Reached consensus**	
Reference: National Cancer Institute. https://www.cancer.gov/publications/dictionaries/cancer-terms/def/overall-survival		
**stereotactic radio-therapy**	agree: 69.2% (9/13)<br>disagree: 7.7% (1/13)<br>unsure: 23.1% (3/13)<br>**Entering the second round**	agree: 93.3% (14/15)<br>disagree: 0<br>unsure: 6.7% (1/15)<br>**Reached consensus**
Reference: 医学名词审委员会. 放射医学与防护名词[J]. 科学出版社, 2014.		

**cognitive function assessment**	agree: 100% (13/13)<br>disagree: 0<br>unsure: 0<br>**Reached consensus**	
Reference: 中国痴呆与认知障碍诊治指南写作组,中国医师协会神经内科医师分会认知障碍疾病专业委员会. 2018 中国痴呆与认知障碍诊治指南(三)：痴呆的认知和功能评估[J]. 中华医学杂志,2018,98 (15): 1125-1129.		
**Bruton tyrosine kinase inhibitor，Btki**	agree: 76.9% (10/13)<br>disagree: 0<br>unsure: 23.1% (3/13)<br>**Reached consensus**	
Reference: Mohamed AJ, Yu L, Bäckesjö CM, et al. Bruton’s tyrosine kinase (Btk): function, regulation, and transformation with special emphasis on the PH domain[J]. Immunological reviews, 2009, 228(1): 58-73.		
